# Unveiled reactivity of masked diformylmethane with enamines forming resonance-assisted hydrogen bonding leads to di-*meta-*substituted pyridines

**DOI:** 10.1038/s42004-024-01228-w

**Published:** 2024-06-28

**Authors:** Sihyeong Yi, Ji Hyae Lee, Hana Cho, Kannan Vaithegi, Dawon Yi, Sijun Noh, Seung Bum Park

**Affiliations:** 1 Department of Chemistry, Seoul National University, Seoul, Korea; 2 Department of Biophysics and Chemical Biology, Seoul National University, Seoul, Korea

**Keywords:** Synthetic chemistry methodology, Chemical libraries, Synthetic chemistry methodology

## Abstract

Pyridine, an essential structure in drug development, shows a wide array of bioactivities according to its substitution patterns. Among the bioactive pyridines, *meta*-substituted pyridines suffer from limited synthetic approaches despite their significance. In this study, we present a condensation-based synthetic method enabling the facile incorporation of biologically relevant functional groups at the *meta* position of pyridine. This methodology unveiled the concealed reactivity of 3-formyl(aza)indoles as diformylmethane analogs for synthesizing dissymmetric di-*meta*-substituted pyridines without *ortho* and *para* substitutions. Furthermore, we uncovered resonance-assisted hydrogen bonding (RAHB) as the requirement for the in situ generation of enamines, the key intermediates of this transformation. Successful development of the designed methodology linked to wide applications—core remodeling of natural products, drug–natural product conjugation, late-stage functionalization of drug molecules, and synthesis of the regioisomeric CZC24832. Furthermore, we discovered anti-inflammatory agents through the functional evaluation of synthesized bi-heteroaryl analogs, signifying the utility of this methodology.

## Abstract (web summary)

Pyridine is an essential structural motif in medicinal chemistry and shows a wide range of bioactivities based on its substitution patterns, however, the meta-substitution of pyridine remains challenging. Here, the authors develop the synthesis of dissymmetric di-meta-substituted pyridines from 3-formyl(aza)indoles through the in situ generation of enamines via resonance-assisted hydrogen bonding, showcasing various synthetic applications in medicinal chemistry.

## Introduction

**Fig. 1:**
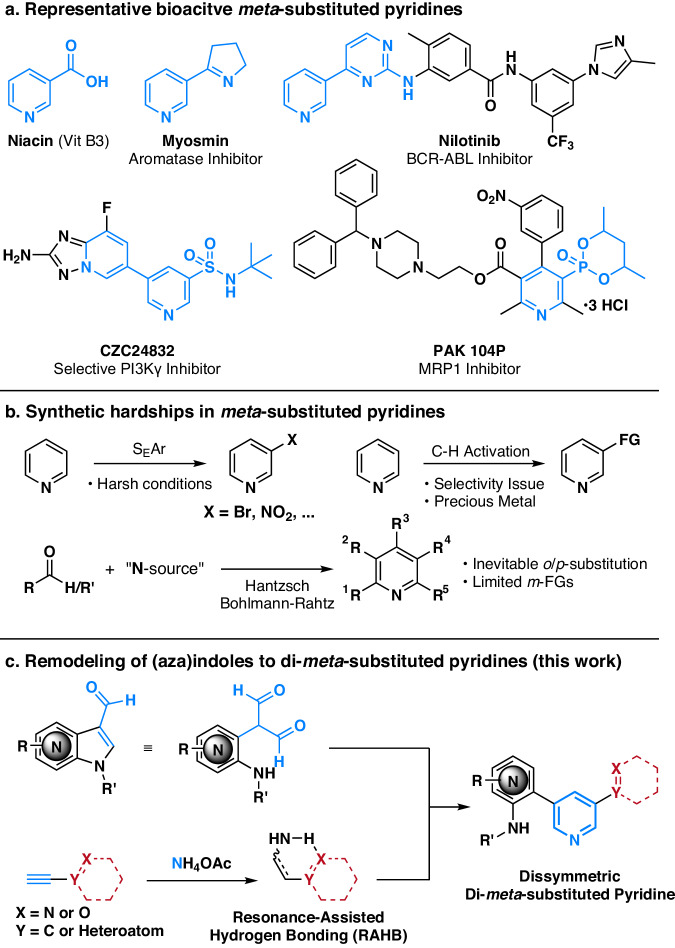
Impetus for construction of dissymmetric di-*meta*-substituted pyridines via (aza)indole core remodeling. **a** Representative bioactive *meta*-substituted pyridines. **b** Conventional synthetic routes for *meta*-substituted pyridines. **c** Metal-free (aza)indole remodeling with terminal acetylenes for the construction of dissymmetric di-*meta*-substituted pyridines (this work).

Pyridine, a nitrogen-containing heteroarene, is a pervasive privileged substructure found in various pharmaceuticals and natural products^1,2^. The intricate interplay between pyridine’s biological relevance and substitution patterns has led to the emergence of numerous bioactive compounds^3–8^. Consequently, developing efficient synthetic methods for selectively functionalized pyridines has become crucial in synthetic chemistry. To note, numerous bioactive pyridines possess substituents at *meta* positions, and their functionalities at the *meta* position extend from simple carboxylate in niacin (vitamin B3) to diverse heterocycles, sulfonamide, and phosphonate (Fig. 1a)^9–12^. Despite their significance and high demand, synthetic approaches toward *meta*-substituted pyridines are constrained due to the inherent low reactivity at the *meta* position of pyridine^13^. In this study, our goal was to devise a synthetic method enabling the seamless incorporation of biologically relevant functional groups at the *meta* position of pyridine, aiming to explore the unique bioactivities of *meta*-substituted pyridines, exemplified by niacin.

Traditional pyridine syntheses can be broadly classified into two categories; direct functionalization of the pyridine ring and the cyclization of acyclic intermediates to the pyridine core (Fig. 1b)^14^. Electrophilic aromatic substitution (S_E_Ar) offers selective functionalization at the *meta* position of pyridine, but it necessitates harsh reaction conditions due to the electron-deficient nature of pyridine^15,16^. C–H functionalization is an alternative effective way for introducing various functional groups at the *meta* position^17^. Recently, two notable methodologies were reported in *Science*: the McNally group presented *meta*-selective halogenation of pyridines through Zincke imine intermediates^18^, and the Studer group reported radical and ionic *meta*-C–H functionalization of pyridines^19^. However, most C–H activation methods still rely on precious metal catalysts or directing groups to achieve *meta*-selective reactivity^13^. Redox-based synthetic methods^20^, arising trends in pyridine *meta*-functionalization, also encountered challenges in energetically unfavorable re-aromatization or highly unstable intermediates, and the introducible *meta*-functionalities were limited to non-oxidation-sensitive groups^19^.

Pyridine syntheses through the cyclization of carbonyl units and nitrogen source, exemplified by Hantzsch pyridine synthesis^21^ and Bohlmann-Rahtz synthesis^22^, have successfully generated numerous bioactive *meta*-substituted pyridines. Nevertheless, these methods often yield inevitable *ortho*- or *para-*substitutions. In the realm of cyclization methods for pyridine synthesis, various reactions, represented by Guareschi-Thorpe pyridine synthesis, can be viewed as condensations between di-carbonyl methane and enamines^23^. Several reports have suggested the utilization of diformylmethane (propanedial) analogs instead of di-ketomethane analogs to circumvent the conventionally inevitable *ortho* or *para* substituents^24–27^. However, prior enamine-based pyridine syntheses still encounter limitations in *meta*-substitution patterns and inevitable *ortho* substitution due to using *β*-keto esters or ketones as common enamine precursors.

In our study, grounded in a thorough analysis of these reaction mechanisms, we envisioned the use of 3-formyl(aza)indoles as a masked diformylmethane moiety that can intermolecularly condense with enamines in situ-generated from diverse terminal acetylenes containing various substituents (Fig. 1c). This masked diformylmethane moiety effectively suppresses potent side reactions and undergoes the desired condensation with in situ-generated enamines, leading to the selective synthesis of a pyridine skeleton with dissymmetrical di-*meta* substitutions and devoid of *ortho* or *para* substituents. Our extensive substrate scope study revealed that the formation of resonance-assisted hydrogen bonding (RAHB) in enamines is critical for the subsequent condensation with 3-formyl(aza)indoles. By diversifying enamines using various terminal acetylenes, we successfully introduced a broad range of functional groups at the *meta* position of pyridines. This process conventionally required harsh reaction conditions or precious metal catalysts. The developed chemical transformation of 3-formyl(aza)indoles found practical applications in the core remodeling of natural products, direct conjugation of drug–natural products, late-stage functionalization of drug molecules, and synthesis of the regioisomeric CZC24832. The resulting library of dissymmetric di-*meta*-substituted pyridine analogs underwent various high-throughput screenings and subsequent biological evaluations, leading to the discovery of anti-inflammatory agents, further validating this synthetic methodology’s significance.

## Results

### Methodology design

**Fig. 2:**
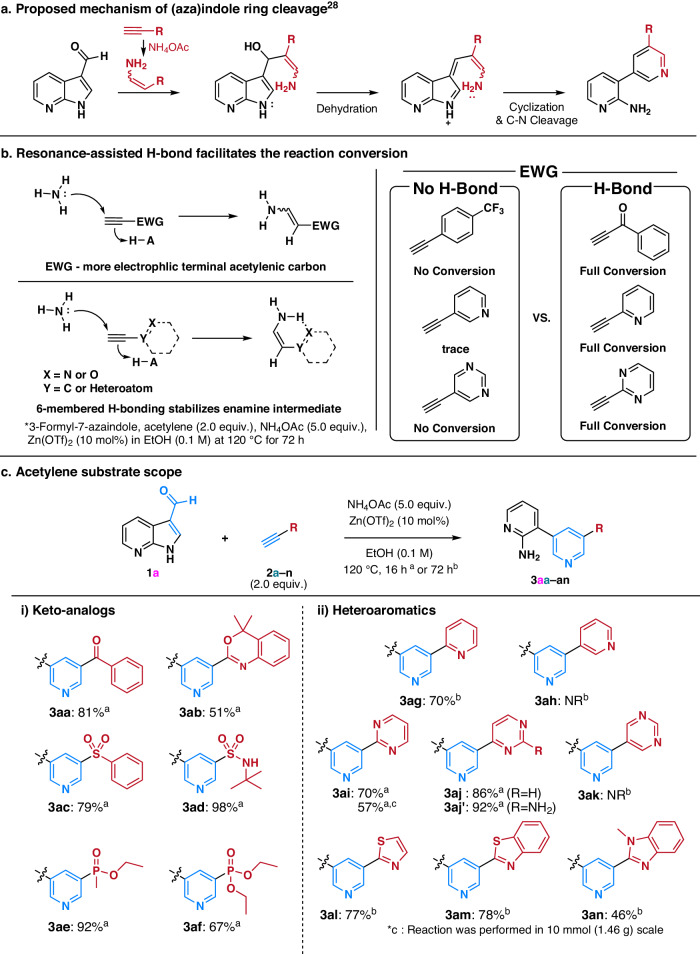
Mechanistic investigation and the methodology development. **a** Methodology design strategy. **b** Resonance-assisted hydrogen bonding (RAHB)/Intramolecular hydrogen bonding of enamine as key facilitator. **c** Terminal acetylene substrate scope.

To overcome the inherent limitation in traditional pyridine synthetic methods—direct functionalization of the pyridine ring and cyclization of acyclic intermediates, we propose a condensation-based approach involving 3-formyl(aza)indoles and various enamines. Enamines, critical intermediates generated in situ from terminal acetylenes using ammonium acetate, play a key role in this process (Fig. 2a)^28^. In generating enamine intermediates, electron-withdrawing groups (EWG) on terminal acetylenes serve as activators, promoting ammonia addition and enamine formation by stabilizing the partial negative charge at the *α*-carbon^29^. Meanwhile, our previous study indicates that EWG does not solely determine enamine formation^28^. NMR studies demonstrated that in situ-generated enamine preferred the *cis-*conformer over the *trans-*conformer, possibly rooted in the formation of resonance-assisted hydrogen bonding (RAHB) within the *β*-amino acrylate structure. RAHB, proposed by Gilli et al., involves intramolecular hydrogen bonding between proton donor and acceptor groups connected through a conjugated π-bonds system, allowing for additional stabilization of the entire structure^30–32^. Various synthetic methodologies have harnessed RAHB for controlling reactivity and facilitating synthon formation^33^. Our hypothesis posits that RAHB can expedite the formation of crucial enamine intermediates from terminal acetylenes with substituents possessing hydrogen bonding acceptors within the resonance system, a pivotal step in the synthesis of di-*meta*-substituted pyridines (Fig. 2b left).

To validate this hypothesis, we subjected 4 classes of terminal acetylenes to the model reaction condition utilizing 3-formyl-7-azaindole (**1a**) as the representative substrate (Fig. S1). Extended reaction time (72 h) was adopted to seize any minimal reaction conversion. Acetylenes having EWGs without RAHB potency (phenyl, 4-trifluoromethylphenyl, 4-nitrophenyl, 3-pyridyl, and 5-pyrimidyl; Fig. S1a) mostly showed no or marginal reaction conversion. Even strong EWG, 4-nitrophenyl acetylene, showed only a limited conversion of 37%. On the other hand, acetylenes having EWGs with RAHB potency (2-pyridyl, 2-pyrimidyl, benzoyl, and diethyl phosphoryl; Fig. S2b) fully converted **1a** to the desired di-*meta*-substituted pyridines. In a comparison, acetylenes having substituents possessing hydrogen bonding acceptors without resonance (dimethyl aminomethyl, methoxymethyl, diethoxylmethyl, and 1-benzotriazolyl methyl; Fig. S1c) did not undergo the desired transformation. It was not a big surprise to find acetylenes having electron-donating/neutral groups did not show any reactivity (4-methoxylphenyl, 4-dimethylaminophenyl, cyclohexen-1-yl, trimethylsilyl; Fig. S1d). Notably, as shown in Fig. 2b (right), acetylenes with structurally analogous substituents exhibited divergent outcomes according to their RAHB potency; 3-pyridyl acetylene showed minimal conversion, whereas RAHB-forming 2-pyridyl acetylene exhibited complete conversion to the desired *meta*-pyridyl pyridine. Similar outcomes were observed in the case of 5-pyrimidyl vs. 2-pyrimidyl acetylenes. Our findings confirmed the necessity of RAHB in the designed methodology.

### Acetylene substrate scope

In the pursuit of optimal reaction conditions, we employed 3-formyl-7-azaindole (**1a**) and diethyl ethynyl phosphonate (**2f**) as model substrates, exploring various parameters such as catalyst, substrate stoichiometry, and reaction time. The effectiveness of different catalysts was evaluated, focusing on Lewis acids, which could potentially enhance enamine formation by coordinating two Lewis bases within a 6-membered H-bonding network of enamine intermediates instead of the proton (Figure S2)^34^. A range of Lewis acids (entries A–E, Table S1), acetylene-activating metal catalysts (entries F–H)^35^, and Brønsted acids (entries I, J) were assessed, and zinc triflate emerged as the most efficient catalyst. Further exploration involving the equivalence of acetylene, catalyst loading, and reaction time determined that optimal conversion required two equivalents of acetylene at an elevated temperature for 16 h (Table S2). Interestingly, increased zinc triflate loading did not improve the reaction conversion (entries I–K). The finalized optimized reaction conditions involved a 0.1 M ethanolic solution of 3-formyl(aza)indoles with 2.0 equivalents of terminal acetylene, 5.0 equivalents of ammonium acetate, and 10 mol% zinc triflate at 120 °C for the corresponding reaction time.

Under the optimized conditions, various acetylenes were employed in the designed transformation with 3-formyl-7-azaindole (**1a**) (Fig. 2c). Terminal acetylenes with keto analogs―ketone (**2a**), carboximidate (**2b**), sulfone (**2c**), sulfonamide (**2d**), phosphinate (**2e**), phosphonate (**2** **f**)―successfully underwent a transformation to yield the desired di-*meta*-substituted pyridines (**3aa**–**3af**). In the case of heteroaryl acetylenes, a complete reaction required 72 h, except for pyrimidine analogs (**2i,** **2j**, and **2j’**). The prolonged reaction time could be attributed to the weaker electron-withdrawing capacities of heteroaryls compared to keto analogs^36^. Terminal acetylenes with 2-pyridyl (**2** **g**), 2-pyrimidyl (**2i**), and 4-pyrimidyl (**2j**) moieties facilitated RAHB formation upon enamine generation, successfully undergoing the desired transformation to yield corresponding *meta-*substituted pyridines (**3ag,** **3ai**, and **3aj**). In contrast, analogs lacking RAHB capability (**2** **h** and **2k**) showed no conversion even after 72 h. Remarkably, the acetylene (**2j’**) containing 2-amino-4-pyrimidine, widely used in Imatinib analog syntheses^37^, produced the desired pyridine analog (**3aj’**) in an excellent yield despite its 2-amino moiety. Terminal acetylenes containing electron-rich 5-membered heteroarenes―thiazole (**2** **l**), benzothiazole (**2** **m**), and benzimidazole (**2n**)―also proved compatible with our methodology, yielding the desired pyridines (**3al**–**3an**). The scalability of our methodology was validated through the gram-scale synthesis of **3ai**, affirming its utility.

Comparatively, traditional methods involve complex steps for introducing functional groups at the *meta* position of pyridines. Our innovative methodology offers a streamlined approach, enabling the robust synthesis of diverse di-*meta-*substituted pyridines in a single step from corresponding terminal acetylenes—eliminating the need for precious transition metal catalysts or corrosive reagents^38^. While conventional approaches rely on heteroarene cyclization^37^ or palladium-mediated C–C bond formation^39^ for introducing heteroaromatics at the *meta* position of pyridines, our methodology provides a more efficient and practical alternative.

### (Aza)Indole substrate scope

**Fig. 3:**
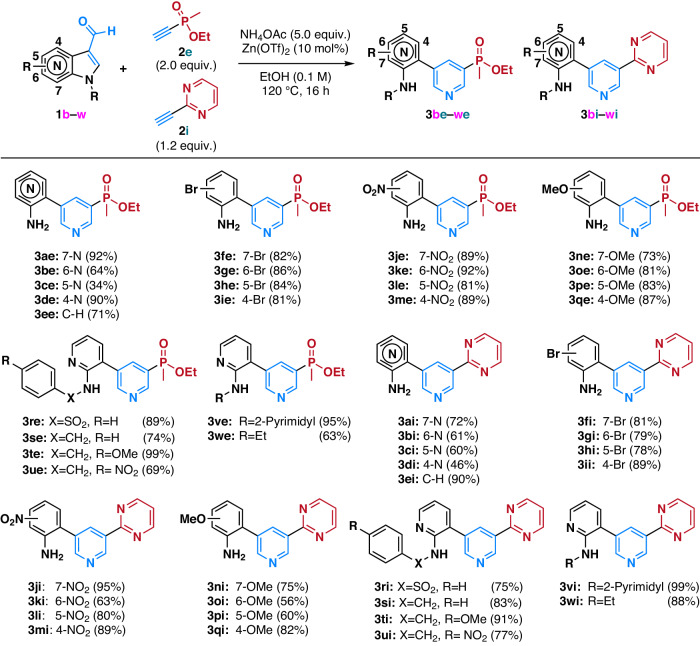
(Aza)indole substrate compatibility table. (Aza)indole substrate scope was explored using ethynyl phosphinate (**2e**) and 2-ethynyl pyrimidine (**2i**) as model acetylene substrates. Blue coloured structures show the compartments constructing newly generated pyridine core. Red coloured structures show the *meta*-substituent originated from acetylene.

Considering reagent scalability and reaction kinetics, we focused our substrate scope study on two model acetylenes―phosphinate (**2e**) and 2-pyrimidine (**2i**)―in the context of 3-formyl(aza)indoles (Fig. 3). We utilized 2.0 equivalents for **2e**, but 1.2 equivalent for **2i** since the 1.2 equivalent of **2i** was enough for the reaction completion within 16 h of reaction time (Table S3). The condensation reaction of all regioisomers of 3-formylazaindoles (**1a–1d**) and 3-formylindole (**1e**) with **2e** and **2i** resulted in the formation of **3ae**–**3ee** and **3ai–3ei**, respectively, in moderate to excellent yields. Notably, all indole isomers featuring bromo substituents (**1f**–**1i**) demonstrated efficient conversion to the desired products (**3fe**–**3ie** and **3fi**–**3ii**) in excellent yields. Emphasizing the utility of bromo substituents as sites for further modifications, the bromo group is challenging to keep intact in conventional metal-catalyzed coupling reactions, signifying the utility of this methodology.

Both electron-withdrawing nitro group (**1j**–**1m**) and electron-donating methoxy group (**1n**–**1q**) on various indole isomers proved amenable to this methodology, yielding the desired products (**3je**–**3qe** and **3ji–3qi**) in good to excellent yields. Subsequently, we explored the substrate scope of *N*-substituted-7-azaindoles, encompassing electron-deficient benzene sulfonyl group (**1r**) to benzyl groups with diverse substituents (**1s**–**1u**). All substrates exhibited clean conversion to the desired products (**3re–3ue** and **3ri–3ui**) in excellent yields. Remarkably, even direct *N*-(pyrimidin-2-yl) 3-formyl-7-azaindoles (**1v**) and *N*-ethyl 3-formyl-7-azaindole (**1w**) demonstrated excellent conversion to the desired products (**3ve,** **3vi,** **3we**, and **3wi**) under the optimized reaction conditions. While no specific pattern of yields based on substrate electronics emerged, a trend indicated that 5- or 6-substituted indoles generally yielded lower than their regioisomers.

To validate the electronic effect on substrate reactivity, we conducted a competitive kinetic study between 5-nitro-3-formylindole (**1l**) and 5-methoxy-3-formylindole (**1p**) using ethynylphosphinate (**2e**) (Figure S3). The competitive reaction resulted in a full conversion of **1l** to **3le**, while **1p** exhibited only a 10% conversion to **3pe**, suggesting that electron-deficient (aza)indoles exhibit faster reaction kinetics than their electron-rich counterparts.

### Synthetic applications

**Fig. 4:**
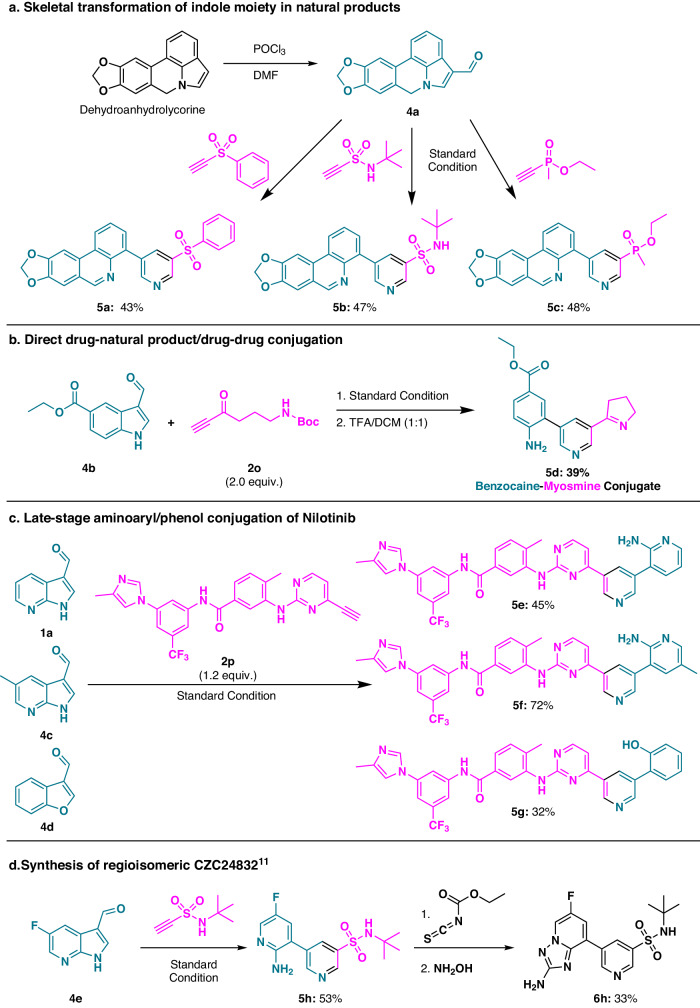
Synthetic application of the developed method. **a** Skeletal transformation of indole-containing natural product. **b** Direct drug–natural product conjugation. **c** Late-stage conjugation of aminoaryl/phenol to anticancer drug, *Nilotinib*. **d** Synthesis of the regioisomeric CZC24832^11^. Turquoise coloured structures originated from diformylmethane analogs. Pink coloured structures originated from acetylene substrates.

In our pursuit of broadening the applications of our developed methodology, we explored its potential in diverse synthetic scenarios, showcasing its versatility and utility (Fig. 4).

Late-Stage Core Remodeling of Natural Products: Utilizing our methodology’s late-stage core remodeling capability, we targeted the transformation of dehydroanhydrolycorine, an indole-fused natural product (Fig. 4a). Dehydroanhydrolycorine was converted to the 3-formylindole derivative (**4a**) via the Vilsmeier-Haack reaction. Treatment of **4a** with **2c,** **2d**, and **2e** under standard conditions led to successful transformations, yielding new skeletons (**5a,** **5b**, and **5c**) in moderate yields. This approach demonstrates the potential to generate distinctive core skeletons while preserving the inherent biological relevance of natural products^40^.

Drug–Natural Product Conjugation: The developed methodology enabled the introduction of diverse functional groups at the *meta* position of pyridine while retaining a biologically relevant aniline moiety and prompted its application in drug–natural product conjugation (Fig. 4b). We exemplified this by directly conjugating benzocaine^41^, a local anesthetic, with myosmine, a naturally occurring nicotine alkaloid^9^, yielding their direct conjugate (**5d**). This strategy allows the creation of compounds with dual bio-relevant features in a single structure.

Late-Stage Modification for Structure-Activity Relationship (SAR) Studies: Late-stage functionalization of bioactive molecules is pivotal for SAR studies. Our methodology’s high reactivity and excellent functional group tolerance make it suitable for late-stage modifications. Inspired by the reactivity of 4-pyrimidyl acetylene (**2j’**), we designed **2p** as a terminal acetylene for late-stage conjugation of Nilotinib, a medication for treating chronic myelogenous leukemia (Fig. 4c). The condensation of **2p** with **1a** yielded an Nilotinib analog (**5e**) with amino-pyridine on the *meta*-position of the pyridine ring. Furthermore, the methodology was applied to 5-methyl-3-formyl-7-azaindole (**4c**) and 3-formyl benzofuran (**4d**), resulting in compounds (**5f** and **5g**) with potential bioactive properties. In particular, 2-amino-5-methyl-pyridine moiety in **5** **f** is an important starting material for synthesizing a well-known insomnia treatment, Zolpidem^42^, which can be a promising example of drug-drug conjugation. To note, 3-formyl benzofuran, another possible masked diformylmethane analog, successfully underwent the desired transformation and introduced the 2-hydroxyphenyl group on the Nilotinib, which proves the expandability of the designed transformation from diverse masked diformylmethane analogs.

Generation of Regioisomeric Bioactive Molecule: To showcase the applicability of our methodology in regioisomer generation, we targeted CZC24832, a selective PI3Kγ inhibitor with a di-*meta-*substituted pyridine core^11^. Despite extensive SAR studies on CZC24832 and its analogs, the regioisomer **6h** had never been achieved due to challenges in coupling *ortho-*aniline at the *meta* position of pyridines. The condensation reaction of 5-fluoro-3-formyl-7-azaindole (**4e**) with sulfonamidyl acetylene (**2d**) enabled the synthesis of the functionalized *meta*-bipyridine **5h**, which underwent subsequent reactions to furnish the regioisomeric CZC24832 (**6h**) in moderate yields (Fig. 4d). This compound retains all the bio-relevant motifs of CZC24832, offering a potentially bioactive molecule with a distinct substituent orientation.

### Discovery of anti-inflammatory agents

Inflammation, a crucial innate defense mechanism, safeguards the body against infections triggered by pathogens, allergens, and toxins, thereby contributing to overall homeostasis^34^. However, when inflammation becomes chronically abnormal, it poses a significant threat, causing severe tissue damage and contributing to various diseases such as sepsis^43^, neurodegenerative disorders^44^, autoimmune conditions^45^, and even certain cancers^46^. Consequently, the urgent need for effective anti-inflammatory agents with innovative mechanisms of action is evident. Non-steroidal anti-inflammatory drugs (NSAIDs), encompassing small-molecule drugs exhibiting anti-inflammatory effects, often feature an aniline moiety in their structures^47^. Meanwhile, pyridines are found in several anti-inflammatory drugs like epibatidine, piroxicam, and niflumic acid, which further underscores the potential of these heterocyclic compounds in combating inflammation^48,49^.

**Fig. 5:**
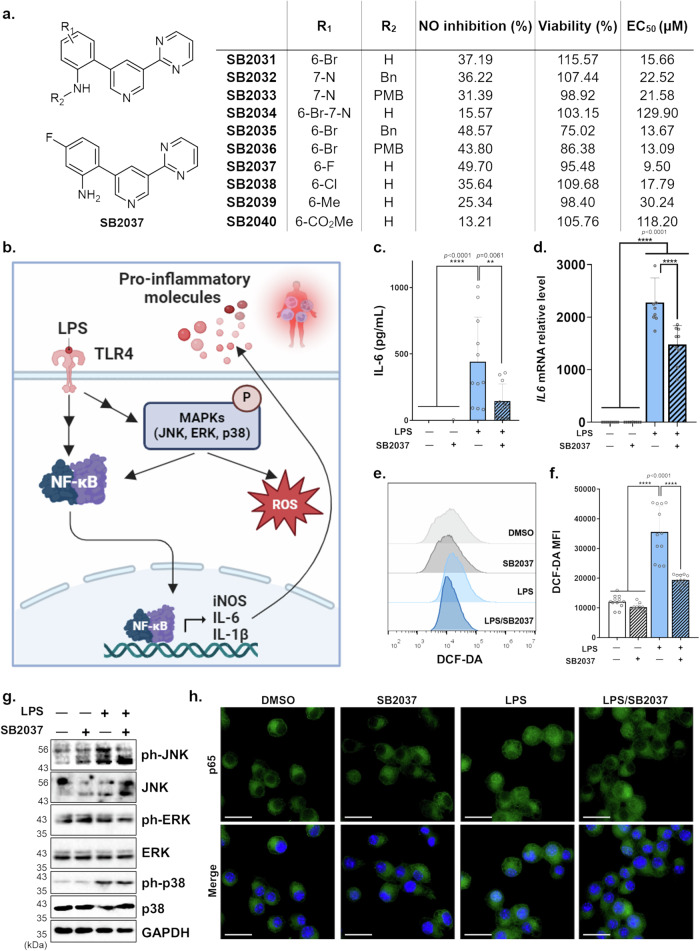
Investigation of an anti-inflammatory agent in macrophages through focused structure-activity relationship study. **a** Structure-activity relationship data for the inhibition of NO release and cellular toxicity in RAW264.7 cells, treated with compounds (10 μM) in the presence of LPS (100 ng/mL) for 24 h. **b** Schematic diagram of LPS-induced signaling pathways in macrophages. **c** IL-6 secretion in RAW264.7 cells treated with LPS (100 ng/mL) or **SB2037** (10 μM) for 24 h (*n* = 11). **d** Relative mRNA expression of *IL6* in RAW264.7 cells treated with LPS (100 ng/mL) or **SB2037** (10 μM) for 5 h (*n* = 9). **e** Flow cytometric results of ROS production in Raw264.7 cells treated with LPS (100 ng/ml) or **SB2037** (10 μM) for 24 h. **f** Quantitative data of **e** (*n* = 12). **g** Immunoblots of MAPK proteins and their phosphorylation levels in RAW264.7 cells treated with **SB2037** (10 μM) in the absence or presence of LPS (100 ng/ml) for 8 h. GAPDH was used as a loading control. **h** Representative immunofluorescence imaging of J774A.1 cells (p65, green) treated with **SB2037** (5 μM) for 1 h, followed by LPS (100 ng/mL) 0.5 h. scale bar = 25 μm. Data were analyzed using one-way ANOVA, followed by Tukey’s *post hoc* test. Experiments were performed more than three times, respectively. *P < 0.05, **P < 0.01, ***P < 0.001, ****P < 0.0001.

Motivated by the structural attributes of *meta*-substituted pyridines conjugated with anilines or aminopyridines, we conceived the exploration of anti-inflammatory agents with constructed bi-heteroaryl compounds (Fig. S4a–d). Employing the Griess assay, a method for assessing nitric oxide (NO) production in live cells^50^, we screened 66 synthesized compounds in mouse macrophage RAW264.7 cells. This screening identified initial hit compounds (**3gi,** **3si**, and **3ti**) exhibiting NO inhibition in response to lipopolysaccharide (LPS), a powerful inflammation inducer. These hit compounds were named **SB2031**, **SB2032**, and **SB2033**, respectively, initiating a subsequent SAR study. Notably, **SB2037**, derived from 6-fluoroindole, emerged as the most potent NO inhibitor with minimal cytotoxicity (Figs. 5a and S4e, f).

To gain insight into the molecular mechanism of **SB2037**, we investigated the cellular inflammatory signaling pathway. Toll-like receptors (TLRs), particularly TLR4, play a pivotal role in recognizing pathogen-associated molecular patterns (PAMPs)^51^. TLR4 responds to gram-negative bacterial infections by recognizing LPS, triggering acute inflammation via the activation of mitogen-activated protein kinases (MAPKs). This cascade results in the nuclear translocation of nuclear factor kappa B (NF-κB) and the production of pro-inflammatory molecules, including NO, IL-6, and IL-1β (Fig. 5b). Confirming the cellular anti-inflammatory effects of **SB2037**, our study demonstrated reductions in IL-6 and IL-1β at both translational and transcriptional levels (Figs. 5c, 5d, and S5a–c, S6). Additionally, **SB2037** treatment effectively diminished the production of reactive oxygen species (ROS), as quantified by flow cytometry using the cell-permeable non-fluorescent probe, dichlorodihydrofluorescein diacetate (DCFH-DA) (Figs. 5e, f and S7). Subsequent investigation into the molecular signaling pathway highlighted **SB2037**’s capability to suppress the activation of MAPKs, including Jun *N-*terminal kinase (JNK), extracellular signal-regulated kinase (ERK), and p38, as evidenced by their decreased phosphorylation levels (Figs. 5g and S8), followed by inhibiting NF-κB nuclear translocation (Figs. 5h, S5d, e, and S9). Our findings demonstrate **SB2037**’s potent ability to restore cellular homeostasis by modulating TLR4-mediated acute inflammatory signaling pathways in macrophages.

## Discussion

During this investigation, a pioneering methodology for the cleavage of (aza)indole rings was developed. This innovative approach involved the utilization of in situ-generated enamines with resonance-assisted hydrogen bonding (RAHB) or an internal hydrogen bonding network, leading to the creation of dissymmetric di-*meta*-substituted pyridines. The process involved leveraging 3-formyl(aza)indole as a concealed diformylmethane for intermolecular condensation with enamines generated in situ from diverse terminal acetylenes. This method provides a versatile platform for synthesizing di-*meta-*substituted pyridines featuring various substituents commonly found in bioactive pyridines. Importantly, the other *meta* position in these compounds is conjugated with highly bio-relevant anilines, phenols, or aminopyridines.

The scope of this methodology was extended to diverse applications, including core remodeling of natural products, direct conjugation of drugs with natural products, late-stage conjugation of bio-relevant aminoaryl/phenol moieties to Nilotinib, and synthesis of the regioisomeric CZC24832. Inspired by the structural features of the newly synthesized di-*meta-*substituted pyridine analogs, we ventured into the realm of immune-modulating agents. Utilizing a cell-based Griess assay and subsequent biological studies, **SB2037** emerged as a potent anti-inflammatory agent. Envisaging the broader impact of this methodology, we anticipate its expansion for the construction of diverse di-*meta-*substituted pyridine heterocycles. In this study, only (aza)indoles and benzofuran were utilized as diformylmethane starting materials, so one of the *meta*-position substituents was *ortho*-aminoaryl or *ortho*-hydroxyphenyl by default. Numerous diformylmethane analogs (e.g. benzothiophene or 3-formylchromone) are expected to be applicable in our methodology, and the expandability of diformylmethane substrate scope will be explored in the near future. Such compounds can serve as a unique and valuable source of molecular diversity in the drug discovery process, offering promising avenues for exploring novel therapeutic agents.

## Methods

### General synthetic procedure

To a mixture of 3-formyl(aza)indole (0.2 mmol), NH_4_OAc (1.0 mmol. 5.0 equiv.), Zn(OTf)_2_ (0.02 mmol, 10 mol%) in EtOH (2.0 ml, 0.1 M) was added acetylene (0.4 mmol, 2.0 equiv.). The reaction mixture was heated to 120 °C and stirred for the indicated time. The reaction progress was monitored by thin-layer chromatography and LC-MS. Upon completion, the reaction mixture was poured into brine, and the organic layer was extracted thrice with DCM. The collected organic layer was dried on anhydrous Na_2_SO_4_(s), filtered through cotton, and concentrated *in vacuo*. The crude mixture was purified by silica-gel flash column chromatography to afford the desired products.

### Synthetic procedure for indole substrate scope; 2-ethynyl pyrimidine (2i)

To a mixture of 3-formyl(aza)indole (0.2 mmol), NH_4_OAc (1.0 mmol. 5.0 equiv.), Zn(OTf)_2_ (0.02 mmol, 10 mol%) in EtOH (2.0 ml, 0.1 M) was added 2-ethynyl pyrimidine (**2i**, 0.24 mmol, 1.2 equiv.). The reaction mixture was heated to 120 °C and stirred for the indicated time. The reaction progress was monitored by thin-layer chromatography and LC-MS. Upon completion, the reaction mixture was poured into brine, and the organic layer was extracted thrice with DCM. The collected organic layer was dried on anhydrous Na_2_SO_4_(s), filtered through cotton, and concentrated *in vacuo*. The crude mixture was purified by silica-gel flash column chromatography to afford the desired products.

### Reporting summary

Further information on research design is available in the Nature Portfolio Reporting Summary linked to this article.

### Supplementary information

Peer Review File

Supplementary Information

Description of Additional Supplementary Files

Supplementary Data 1

Supplementary Data 2

Reporting Summary

## Data Availability

The data generated in this study are provided in the Supplementary Information file. The experimental procedures, NMR data, and LRMS or HRMS have been deposited in the Supplementary Information file. NMR Spectra of all newly synthesized products are provided as Supplementary Data 1. Source data of biological outcomes are provided as Supplementary Data 2. All data are available from the corresponding author upon request.
