# Supplementary material for: Unveiled reactivity of masked diformylmethane with enamines forming resonance-assisted hydrogen bonding leads to di-meta-substituted pyridines
Source: Commun Chem. 2024 Jun 28;7:146. doi: 10.1038/s42004-024-01228-w (PMC11213866; doi:10.1038/s42004-024-01228-w)
Supplement: Supplementary file 2 — Supplementary Information [file 42004_2024_1228_MOESM2_ESM.pdf]

< Supplementary Information >

Unveiled reactivity of masked diformylmethane with enamines forming resonance-assisted hydrogen bonding leads to di-meta-substituted pyridines

*Sihyeong Yi†, Ji Hyae Lee†, Hana Cho†, Kannan Vaithegi, Dawon Yi, Sijun Noh, and Seung Bum Park**

Table of Contents — Page No.

**1**	Supplementary Methods	S2–S5
**2**	Supplementary Figures and Tables	S6–S15
**3**	Synthetic Procedures & Characterization	S16–S50
**4**	Supplementary References	S51–S52

1. Supplementary Methods

Chemical synthesis

All commercially available reagents were used without further purification unless noted otherwise. Dried solvents and triethylamine were passed through solvent purification systems equipped with activated alumina columns (Glass Contour). NMR analyses were carried out using Agilent 400-MR DD2 Magnetic Resonance System [400 MHz, Agilent, USA], Bruker Ascend 500 [500 MHz, Bruker, Germany], or Bruker Avance 600 [600 MHz, Bruker, Germany] spectrometers. The chemical shift values of the proton (^{1}H) and carbon (^{13}C) NMR spectra were reported in parts per million (ppm) relative to the internal standard tetramethylsilane (TMS) (^{1}H: 0.00 ppm) or the residual solvent peak ($CDCl_3$, ^{1}H: 7.26 ppm, ^{13}C: 77.16 ppm; DMSO-d_6, ^{1}H: 2.50 ppm, ^{13}C: 39.52 ppm). The multiplicities of the ^{1}H NMR peaks are reported as follows: s (singlet), d (doublet), t (triplet), q (quartet), p (pentet), m (multiplet), dd (doublet of doublets), dt (doublet of triplets), td (triplet of doublets), qd (quartet of doublets), and br s (broad singlet). The coupling constants (*J*) were reported in Hz. Low-resolution mass spectrometry (LRMS) spectra were obtained on an LCMS-2020 spectrometer [Shimadzu, Japan] using the electrospray ionization (ESI) method. High-resolution mass spectra were analyzed using the ESI mode of an Orbitrap Exploris 120 mass spectrometer [Thermo Fisher Scientific, USA]. To monitor the progress of the reactions, analytical thin-layer chromatography (TLC) analysis was performed using glass plates precoated with silica gel [60 F_{254}, Merck, Germany], and the components were observed under UV light (254 and 365 nm) or by treating the plates with ninhydrin or $KMnO_4$ followed by thermal visualization. Flash column chromatography was performed on Merck Kieselgel 60 (230-400 mesh) using Biotage® Selekt [Biotage, Sweden] and Isolera One [Biotage, Sweden]. Microwave reactions were performed using the Biotage® Robot Eight [Biotage, Sweden]. HPLC purification was performed on LC-6 AD Pump and SPD-10A detector system with a YMC-Pack ODS-A C_{18} (AA12S05-2520WT, 250 × 20.0 mm I.D. S-5 μm, 12 nm) column at a flow rate of 8.0 mL/min of eluent solutions with the gradient of 10% B/A to 100% B. (Solution A: water with 0.1% (v/v) trifluoroacetic acid (TFA) and Solution B: acetonitrile (ACN) with 0.1% (v/v) TFA). All the chemical compounds submitted to biological assays were HPLC-purified prior to biological administration.

Programs

Biological graphic (Figures 5b) was drawn in Biorender.com. The provided graphs were analyzed with GraphPad Prism 8 software (GraphPad, San Diego, CA, USA).

Cell culture

RAW264.7 and J774A.1 macrophage cell lines were obtained from American Type Culture Collection and cultured in Dulbecco's Modified Eagle's Medium (DMEM) [Welgene] or in Roswell Park Memorial Institute (RPMI) 1640 medium [Gibco™] supplemented with heat-inactivated 10% (v/v) fetal bovine serum (FBS)

[Gibco^{TM}], 1% (v/v) penicillin (100 units/mL)/streptomycin (100 μg/mL) [Gibco^{TM}], and Fungizone (0.25 μg/mL) [Gibco^{TM}]. All cells were maintained at 5% CO_2 in a 37 °C incubator.

Griess assay

The Griess assay was used to quantify the cellular secretion of nitric oxide (NO). RAW264.7 cells were seeded on a clear flat bottom TC-treated 96-well plate [Corning]. After 24 h, cells were treated with compounds in the absence or presence of lipopolysaccharide (LPS) (100 ng/mL) (Sigma-Aldrich, L4391). After 24 h of incubation, 90 μL of the cell culture media were reacted with the same volume of Griess reagent (0.1% naphthyl ethylenediamine dihydrochloride and 1% sulfanilamide in 2% phosphoric acid). Absorbance was measured at 550 nm using a microplate reader [Molecular Devices]. The level of NO was estimated by comparison with a sodium nitrite (Alfa Aesar, A18668) standard curve.

Cell viability assay

Cell viability was measured with WST (water-soluble tetrazolium salt; EZ-Cytox (Dogen, EZ-BULK150)) assay kits, and the experimental procedure was based on the manufacturer's manual.

Flow cytometry analysis

RAW264.7 cells were treated with each compound for 24 h as described in the figure legends. For the reactive oxygen species (ROS) measurement, 10 μM of dichlorodihydrofluorescein diacetate (DCFH-DA, Sigma-Aldrich, 35845) was added to the cells for an additional 0.5 h. The resulting cells were subjected to flow cytometry analysis using CytoFLEX LX II [Beckman Coulter], installed at Danaher-SNU Discovery Center at Seoul National University. Data were analyzed to measure the fluorescence intensity of ROS using FlowJo V10 software. Mean values of FITC fluorescence intensity per cell were used for the analysis.

ELISA

The secretion of IL-6 was measured using ELISA kit (R&D Systems, DY406) according to the manufacturer's instructions. RAW264.7 cells were treated as described in the figure legends. After incubation, the levels of cytokine molecules in the culture medium were measured with each cytokine-specific antibody as the capture antibody and detection antibody. The biotinylated antibodies were detected by sequential incubation with streptavidin–horseradish peroxidase (HRP) conjugate and tetramethylbenzidine (TMB) substrates (R&D Systems, DY999). After 20-min incubation, the color development was stopped by adding 2 N H_2SO_4. The absorbance was then read at 450 and 540 nm using a microplate reader [Molecular Devices].

RNA extraction and quantitative real-time PCR (qRT-PCR)

To analyze the levels of cytokine genes, RAW264.7 cells were treated with 100 ng/mL LPS in the presence or absence of 10 µM SB2037. Total RNA was extracted using the RNeasy kit [Qiagen] according to the manufacturer's instructions. RNA was quantified using Nanodrop [ThermoFisher Scientific], and cDNAs were prepared with AccuPower CycleScript RT PreMix dT20 [Bioneer] according to the manufacturer's instructions. Quantitative RT-polymerase chain reaction (qRT-PCR) experiments were conducted with KAPA SYBR FAST ABI Prism qPCR Master Mix [KAPA Biosystems]. The comparative Ct method analyzed the data and normalized them against housekeeping genes.

Primer sequences used for qRT-PCR

IL6 Forward : 5'- TCC AGT TGC CTT CTT GGG AC -3'

IL6 Reverse : 5'- GTA CTC CAG AAG ACC AGA GG -3'

IL1B Foward : 5'- AAG TTG ACG GAC CCC AAA AGA T -3'

IL1B Reverse : 5'- TGT TGA TGT GCT GCT GCG A -3'

Gapdh Forward : 5'- TGG GCT ACA CTG AGC ACC AG -3'

Gapdh Reverse : 5'- GGG TGT CGC TGT TGA AGT CA -3'

Immunoblotting (IB)

Cells were collected and lysed in modified radioimmunoprecipitation assay (RIPA) buffer (50 mM Tris–HCl, pH 7.8, 150 mM NaCl, 1% NP-40, 0.5% deoxycholate, 5 mM NaF, 2 mM Na_3VO_4, 1× Protease Inhibitor Cocktail [Roche]), followed by centrifugation at 20,000× g for 15 min at 4 °C for the whole-cell analysis and the determination of total protein concentration in each lysate using Pierce BCA Protein Assay Kit [ThermoFisher Scientific]. Equal amounts of each lysate were fractionated by PAGE, transferred to PVDF membrane [Bio-Rad], and blocked with 2% BSA [MP Biomedicals] in Tris buffered saline with Tween20 (TBST) [Sigma-Aldrich] for 1 h at room temperature (r.t.). Membranes were probed with protein-specific antibodies overnight at 4 °C. The next day, membranes were washed three times in TBST and incubated for 1 h at r.t. with anti-rabbit or anti-mouse HRP secondary antibody [Cell Signaling Technology] in TBST containing 2% BSA. After washing, membranes were exposed to the detection reagent [GE Healthcare] and quantified using chemiluminescence [Bio-Rad].

The following antibodies were purchased as primary antibodies for immunoblot studies: rabbit anti-SAPK/JNK polyclonal antibody (Cell Signaling Technology, 9252); rabbit anti-phospho-SAPK/JNK (Thr183/Tyr185) polyclonal antibody (Cell Signaling Technology, 9251); rabbit anti-p44/42 MAPK (Erk1/2) monoclonal antibody (Cell Signaling Technology, 4695); rabbit anti-phospho-P44/42 MAPK (Erk1/2) (Thr202/Tyr204) polyclonal antibody (Cell Signaling Technology, 9101); rabbit anti-p38 MAPK polyclonal antibody (Cell Signaling Technology, 9212); rabbit anti-phospho-p38 MAPK (Thr180/Tyr182) polyclonal antibody (Cell Signaling Technology, 9211); rabbit anti-IL-1β monoclonal antibody (Cell Signaling Technology, 31202); rabbit anti-Cleaved-IL-1β monoclonal antibody (Cell Signaling Technology, 63124); rabbit anti-NF-κB p65 polyclonal antibody (Abcam, ab16502); rabbit anti-c-Jun polyclonal antibody (Abcam, ab31419); rabbit anti-Lamin B1 polyclonal antibody (Abcam, ab16048); rabbit anti-β-tubulin polyclonal antibody (Cell Signaling Technology, 2146); rabbit anti-GAPDH monoclonal antibody (Cell Signaling Technology, 2118).

Immunofluorescence (IF) staining

J774A.1 cells were treated with LPS and SB2037 as described in the figure legends, then fixed with 4% paraformaldehyde for 20 min at r.t. After permeabilization with 0.1% Triton X-100-containing PBS for 20 min, the fixed cells were blocked with 1% bovine serum albumin (BSA)-containing PBS for 1 h and incubated with protein-specific antibodies at 4 °C overnight. After washing with PBS, a fluorescent-labeled antibody was added to the sample, incubated for 1 h at r.t., and washed with PBS. Nuclei were visualized by medium-diluted Hoechst 33342 (2 μg/mL) [ThermoFisher Scientific] for 20 min. Samples were analyzed by DeltaVision Elite imaging system [Cytiva]. Images were obtained with a 60× scale using GFP/GFP (excitation: 475/28 nm, emission: 525/48 nm) and DAPI (excitation: 390/18 nm, emission: 435/48 nm) filter sets. Images were analyzed and merged with SoftWorks deconvolution software [Cytiva]. The following antibodies were purchased for immunofluorescence studies: mouse anti-p65 antibody (Santa Cruz Biotechnology, sc-8008,); goat anti-mouse IgG (FITC) (Abcam, ab6785).

Subcellular fractionation assay

After SB2037 treatment in the absence or presence of LPS as described in the figure legends, RAW264.7 cells underwent subcellular fractionation with NE-PER nuclear and cytoplasmic extraction reagents [ThermoFisher Scientific, 78833] according to the manufacturer's protocol. The resulting samples were analyzed by SDS-PAGE and immunoblotting. The purity of the nuclear and cytosolic fractions was confirmed by the cytosolic marker protein GAPDH and the nuclear marker protein LMNB1, respectively.

2. Supplementary Figures and Tables

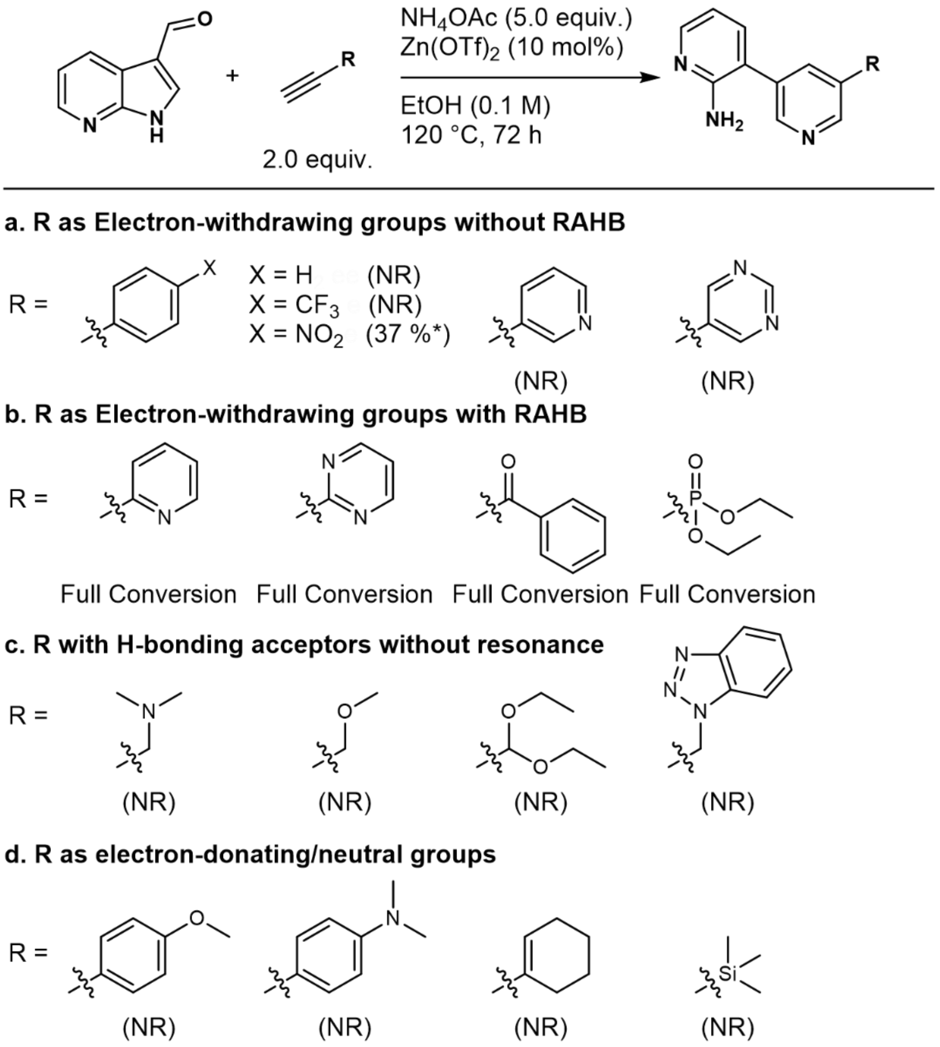

Fig. S1 | The role of resonance-assisted hydrogen bonding (RAHB) in the reaction conversion. Acetylenes in four categories were screened. **a** Acetylenes having R groups as electron-withdrawing groups without RAHB. **b** Acetylenes having R groups as electron-withdrawing groups with RAHB. **c** Acetylenes having R groups as with hydrogen bonding acceptors without resonance. **d** Acetylenes having R group as electron-donating/neutral groups. **NR** stands for no reaction. Conversion rates were determined based on the PDA area by LC-MS analysis. *: Percentage was calculated as (Desired Product Area/(Desired Product Area + Starting Material Area)×100 (%)

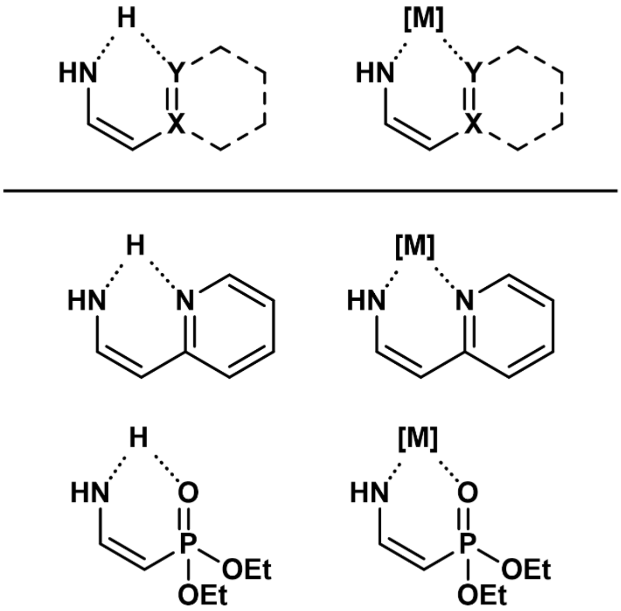

Fig. S2 | The role of Lewis acid as a catalyst.

Lewis acid can stabilize the enamine intermediate to form an RAHB-like structure.

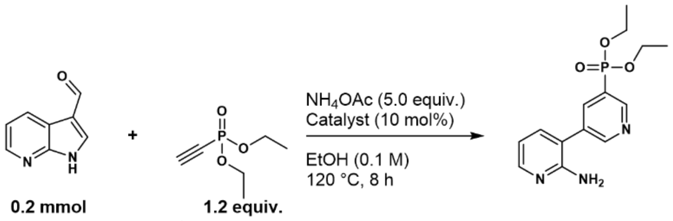

Entry	Catalyst (10 mol%)	Conversion
A	$Zn(OTf)_2$	78 %
B	$Yb(OTf)_3$	65 %
C	$Ni(OTf)_3$	74 %
D	$Fe(OTf)_3$	76 %
E	$AlCl_3$	52 %
F	AuCl	53 %
G	$AuCl_3$	68 %
H	Ag(OTf)	50 %
I	TFA	46 %
J	PTSA	52 %

Table S1 | Acid catalyst screening. The conversion rate was determined based on the PDA area by LC-MS analysis. The blue box is the optimal reaction condition.

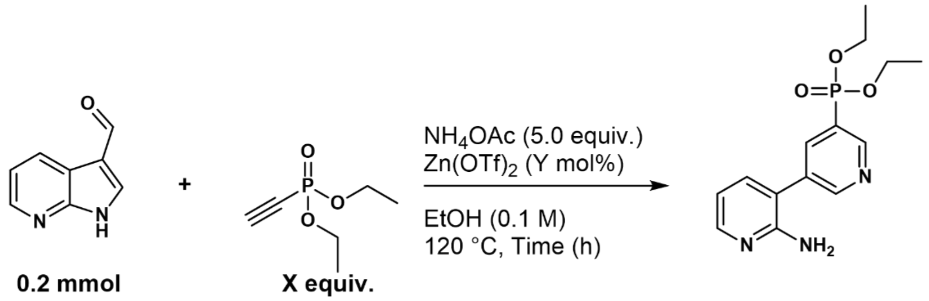

Entry	X (equiv.)	Y (mol%)	Time (h)	Conversion
A	1.2	10	8	59 %
B	1.2	10	12	60 %
C	1.2	10	16	65 %
D	1.5	10	8	68 %
E	1.5	10	12	84 %
F	1.5	10	16	79 %
G	2.0	10	8	83 %
H	2.0	10	12	88 %
I	2.0	10	16	91 %
J	2.0	20	16	92 %
K	2.0	40	16	90 %

Table S2 | Condition screening using ethynylphosphonate as model substrate for keto analogs. The conversion rate was determined based on the PDA area by LC-MS analysis. The blue box is the optimal reaction condition.

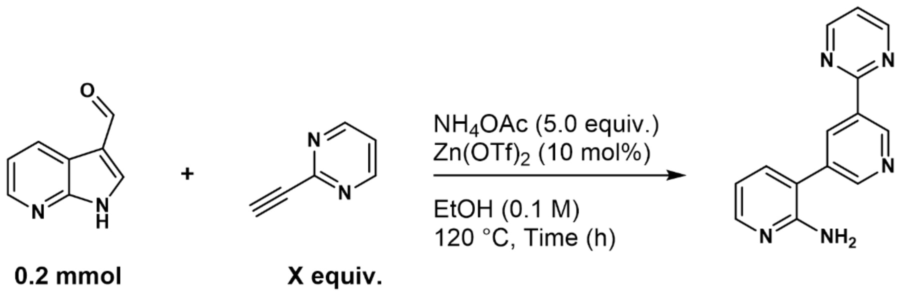

Entry	X (equiv.)	Time (h)	Conversion
A	1.2	8	89 %
B	1.2	12	88 %
C	1.5	8	Full Conv.
D	1.5	12	Full Conv.
E	2.0	8	Full Conv.
F	2.0	12	Full Conv.
G	1.2	16	Full Conv.

Table S3 | Condition screening using 2-ethynylpyrimidine as model substrate for heteroarene analogs. The conversion rate was determined based on the PDA area by LC-MS analysis. The blue box is the optimal reaction condition.

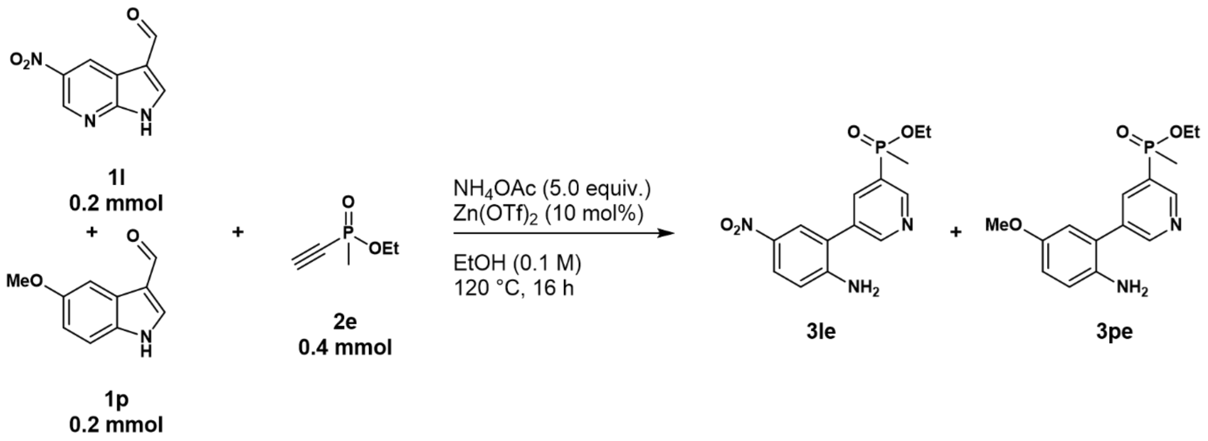

Area Percentage			
**1l**	**3le**	**1p**	**3pe**
0 %	50 %	45 %	5 %
**Conversion Rate**			
**1l**	100 %	**1p**	10 %

Fig. S3 | Competitive kinetic study. The conversion rate was determined based on the PDA area by LC-MS analysis.

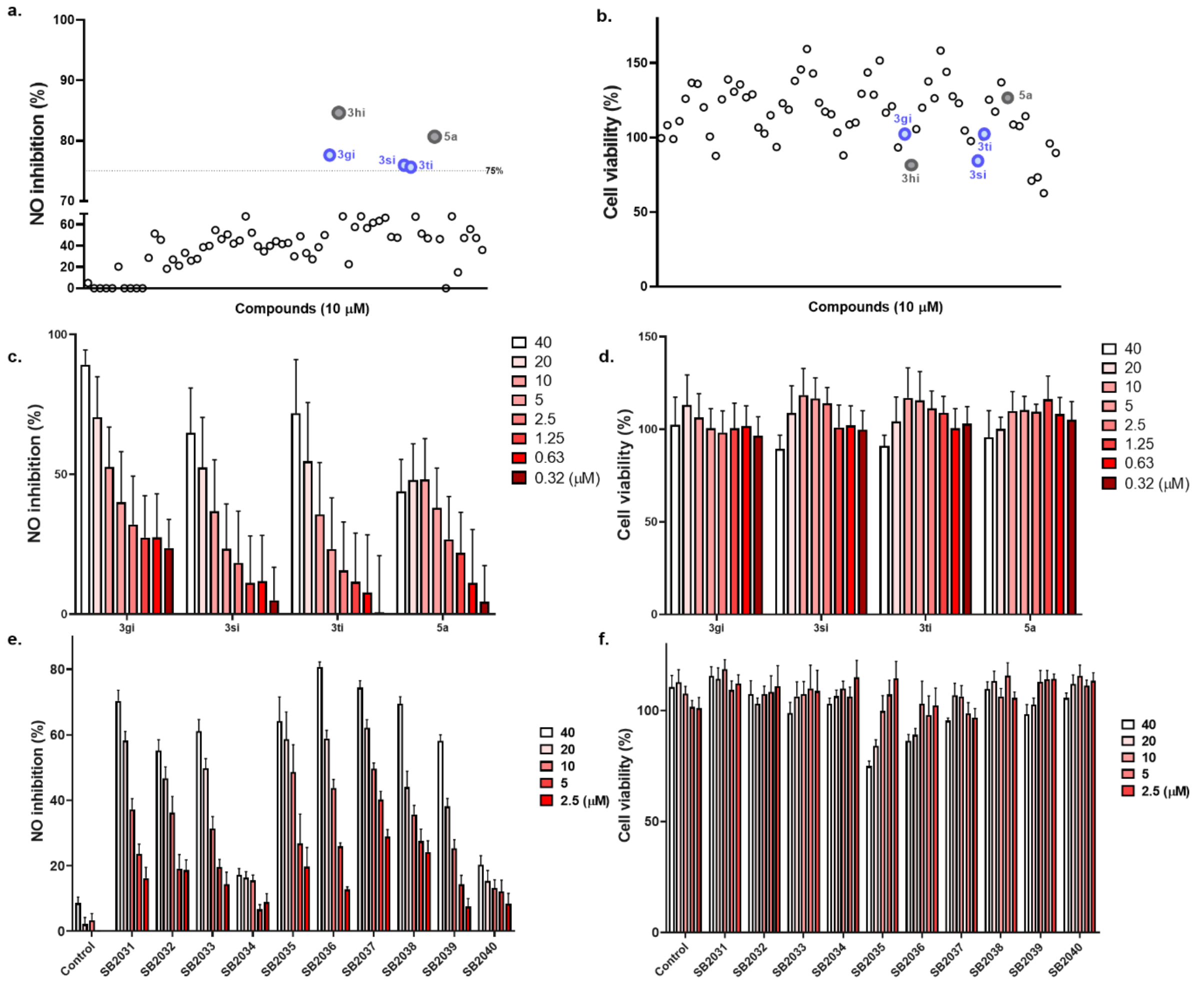

Fig S4 | Results of phenotypic screening assay targeting NO release in acute inflammation-induced macrophages. a Initial screening results for the inhibition of NO release in RAW264.7 cells, treated with compounds (10 μM) in the presence of LPS (100 ng/mL) for 24 h. **b** Cellular toxicity results in RAW264.7 cells in the presence of LPS (100 ng/mL) for 24 h treatment. **c** Dose-dependent effects of initial hit compounds on RAW264.7 cells in the presence of LPS (100 ng/mL) stimulation. The release levels of nitrite were measured by Griess assay. **d** Cellular toxicity was detected on RAW264.7 cells in dose-dependent effects of the initial hit compounds in the presence of LPS (100 ng/mL) for 24 h treatment. **e** Dose-dependent effects of focused library compounds on RAW264.7 cells in the presence of LPS (100 ng/mL) stimulation. The release levels of nitrite were measured by Griess assay. **3ei** compound was used as a control compound. **f** Cellular toxicity was detected on RAW264.7 cells in dose-dependent effects of the focused library compounds in the presence of LPS (100 ng/mL) for 24 h treatment.

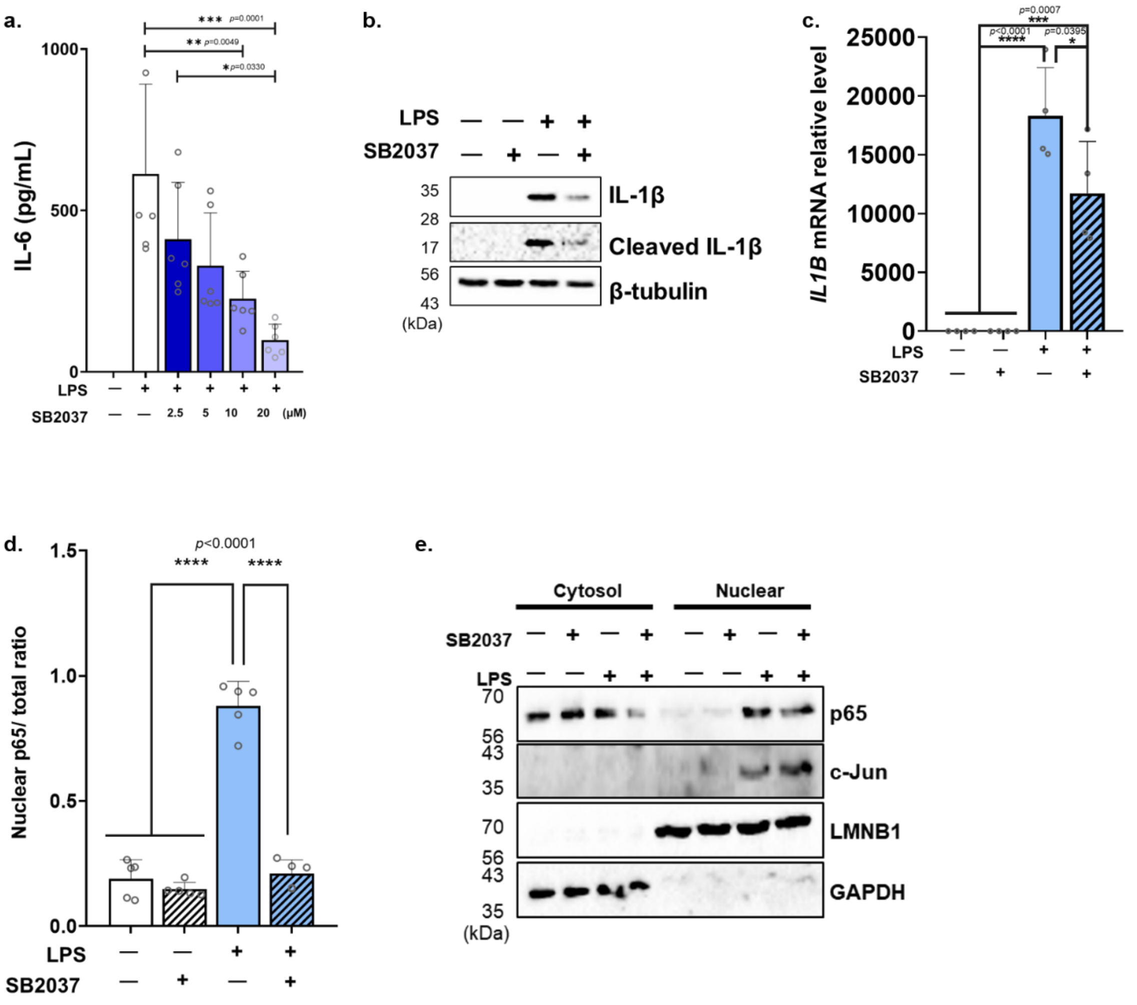

Fig. S5 | Exploration of anti-inflammatory activity of SB2037 in LPS-induced macrophages. a Quantification of IL-6 cytokine secretion in RAW264.7 cells treated with LPS (100 ng/mL) or SB2037 (10 µM) for 24 h (n=6). **b** Immunoblot analysis of IL-1β in RAW264.7 cells treated with LPS (100 ng/mL) or SB2037 (10 µM) for 8 h. **c** Relative mRNA expression of *IL1B* in RAW264.7 cells treated with LPS (100 ng/mL) or SB2037 (10 µM) for 5 h (n=4). **d** Quantification data of **Figure 5h** (n=5). **e** Immunoblots of nuclear/cytosol fractionation assay for transcription factors in RAW264.7 cells treated with SB2037 (10 µM) for 30 m, followed by LPS (100 ng/mL) for 20 m. Data were analyzed using one-way ANOVA, followed by Tukey's *post hoc* test. Experiments were performed more than three times, respectively. *$P < 0.05$, **$P < 0.01$, ***$P < 0.001$, ****$P < 0.0001$.

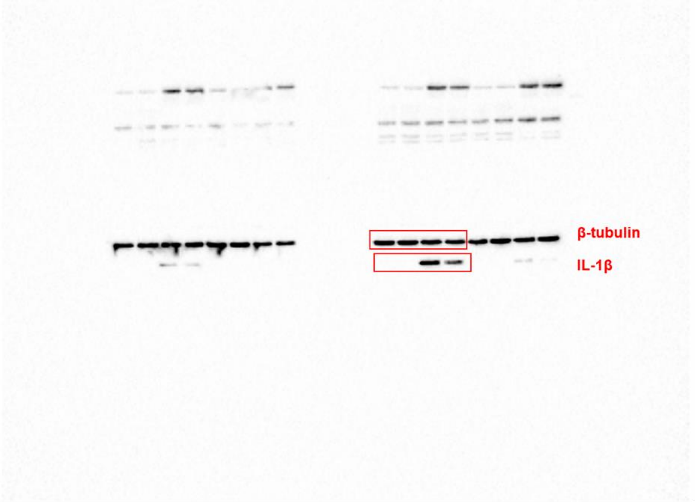

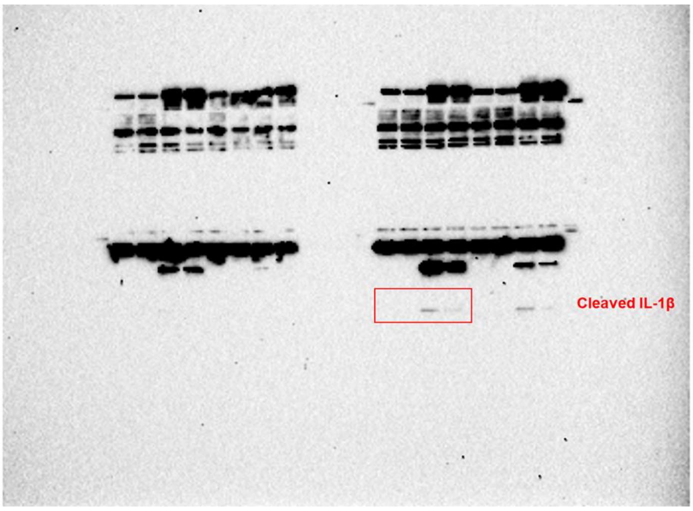

Fig. S6 | Sourceblot data of Figure S5b.

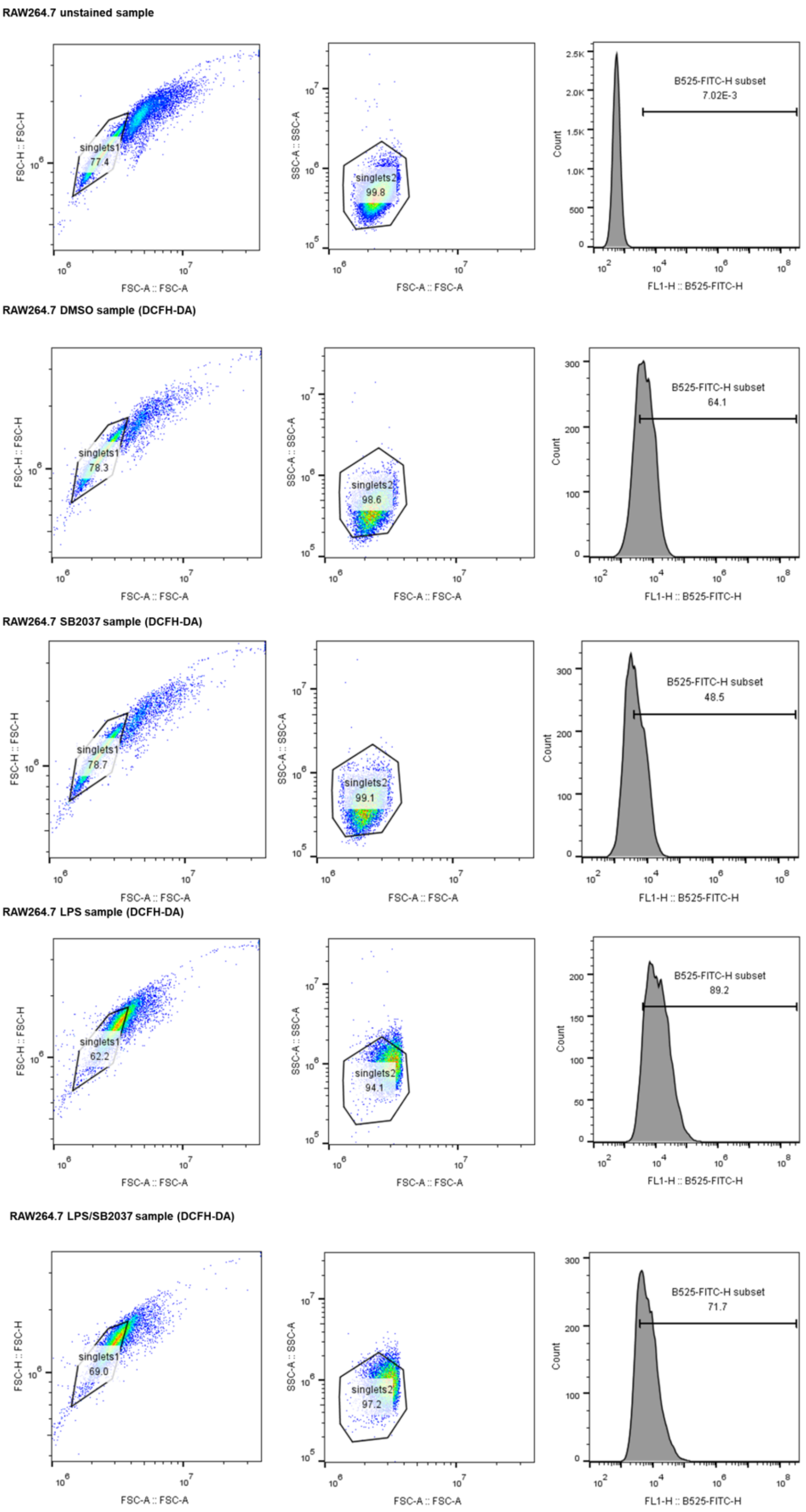

Fig. S7 | Gating strategy of flow cytometry analysis in Figure 5e and f.

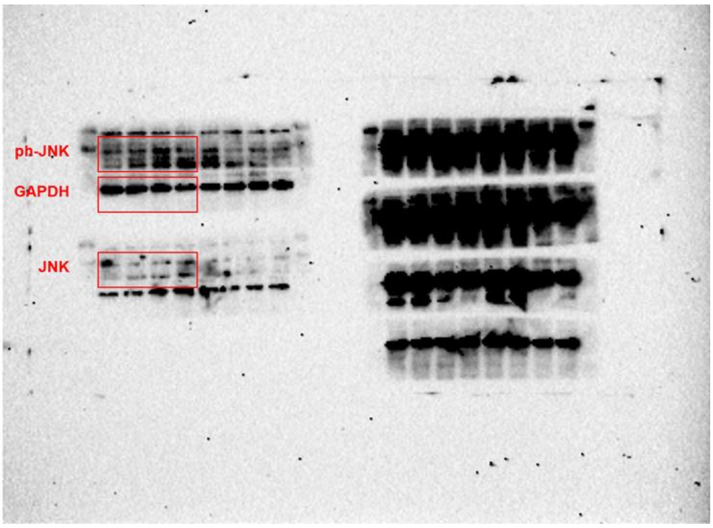

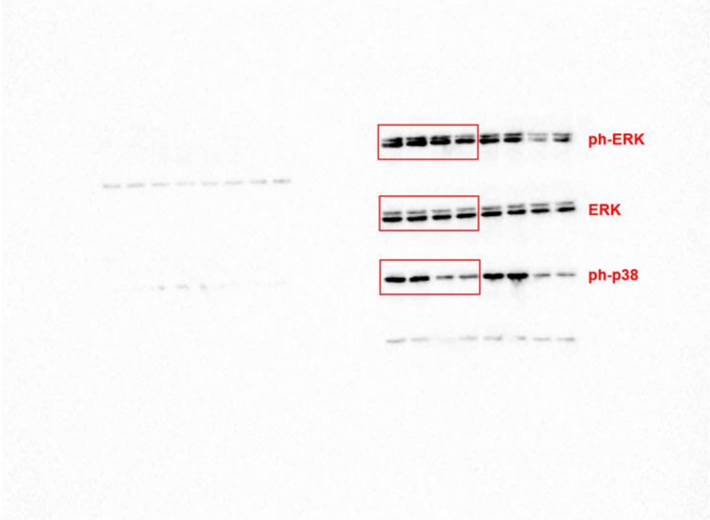

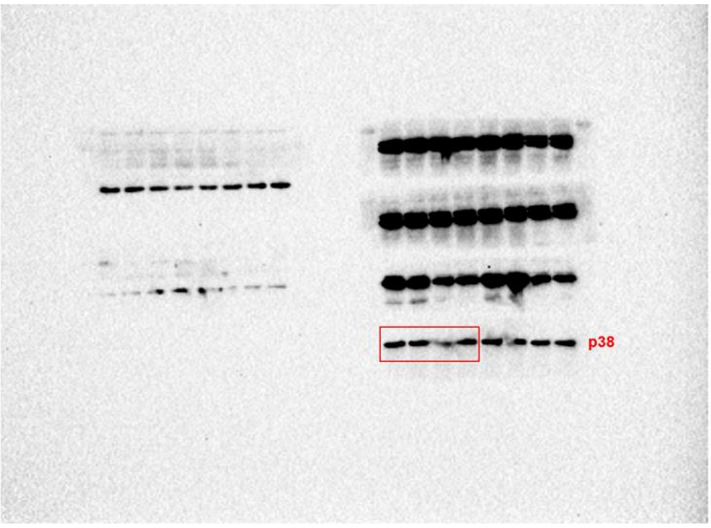

Fig. S8 | Sourceblot data of Figure 5g.

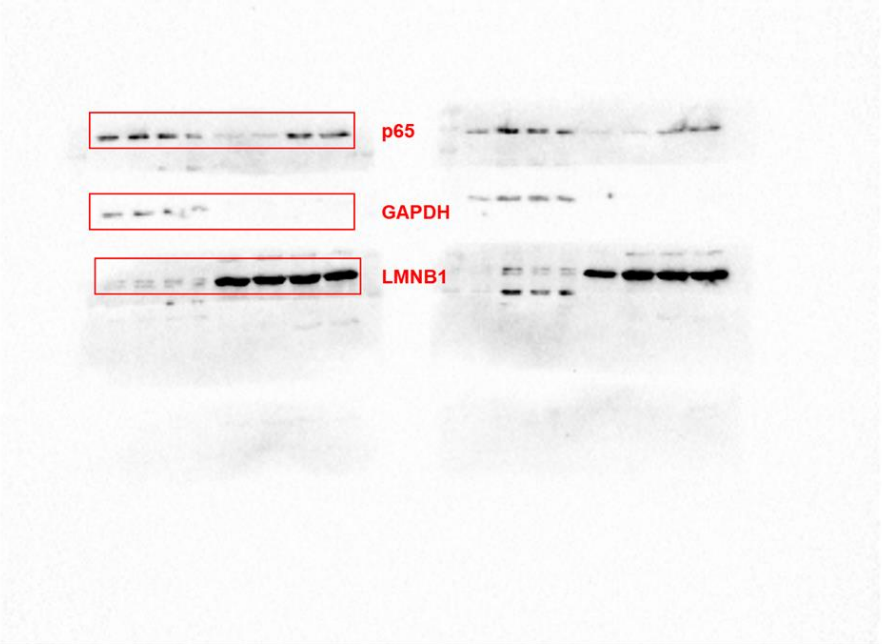

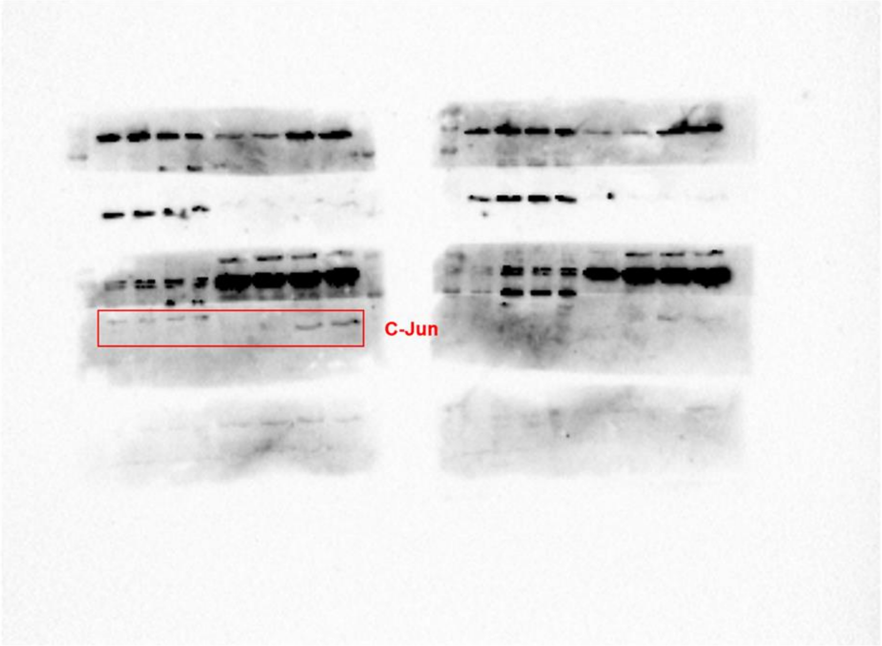

Fig. S9 | Sourceblot data of Figure S5e.

3. Synthetic Procedures & Characterization

Fig. S10 | Starting materials (1a–1w) used in the substrate scope study

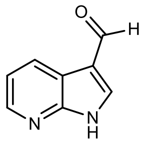

1a
Commercially Available
Combi-Blocks

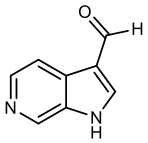

1b

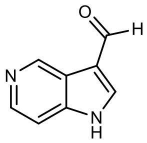

1c

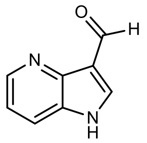

1d

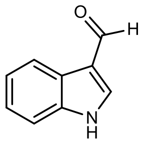

1e
Commercially Available
Sigma-Aldrich

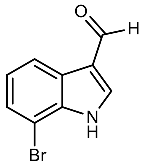

1f

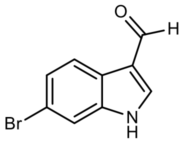

1g
Commercially Available
TCI

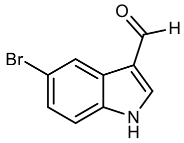

1h
Commercially Available
Sigma-Aldrich

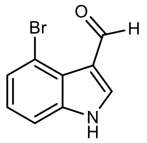

1i
Commercially Available
Alfa Aesar

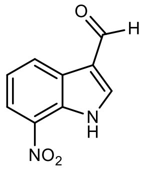

1j

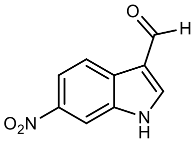

1k

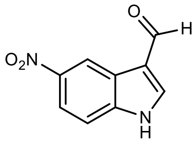

1l
Commercially Available
TCI

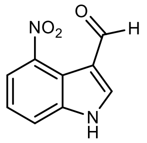

1m
Commercially Available
Alfa Aesar

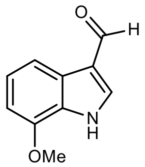

1n

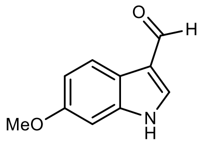

1o
Commercially Available
Alfa Aesar

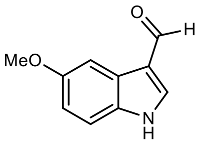

1p
Commercially Available
Sigma-Aldrich

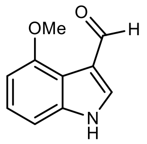

1q
Commercially Available
Sigma-Aldrich

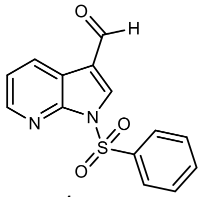

1r

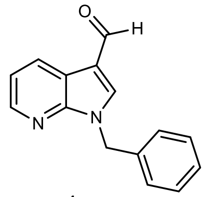

1s

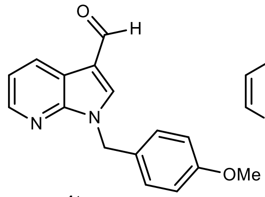

1t

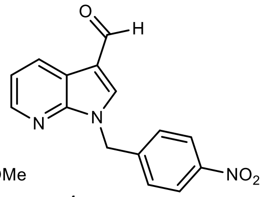

1u

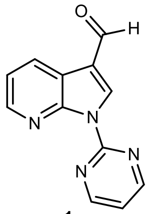

1v

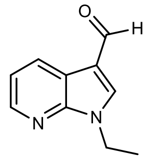

1w

Fig. S11 | Starting materials (4a–4l) used in applications & SAR study

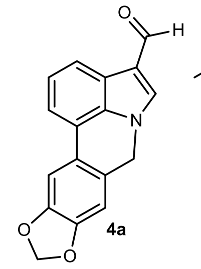

4a

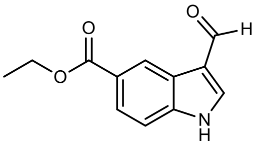

4b

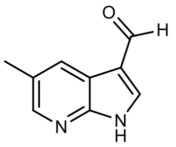

4c

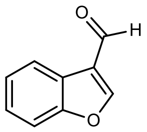

4d

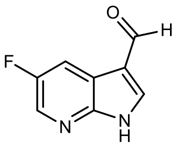

4e

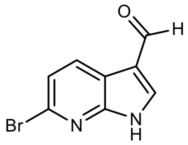

4f

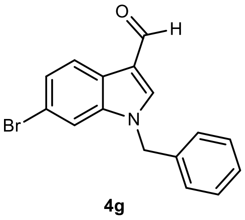

4g

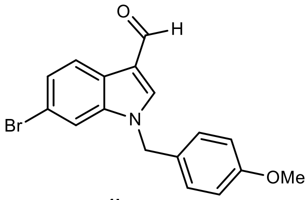

4h

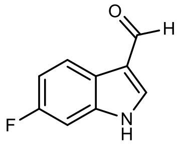

4i
Commercially Available
Alfa Aesar

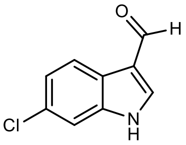

4j

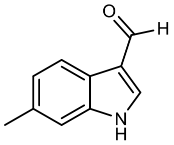

4k
Commercially Available
Alfa Aesar

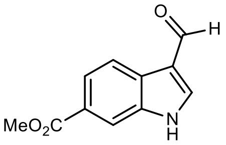

4l

Fig. S12 | Acetylenes used in this methodology (2a–2p)

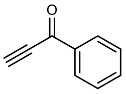

2a

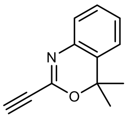

2b

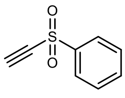

2c

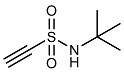

2d

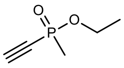

2e

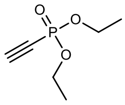

2f

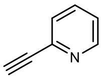

2g
Commercially Available
Acros

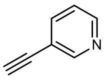

2h
Commercially Available
Sigma-Aldrich

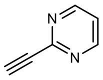

2i

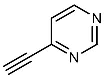

2j

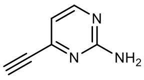

2j'

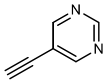

2k

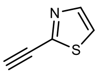

2l

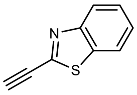

2m

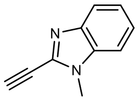

2n

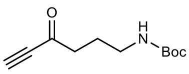

2o

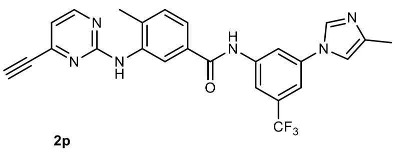

2p

3.1. Synthetic procedure of starting materials (1a–1w & 4a–4l)

Most of 3-formyl-(aza)indoles (**1a**–**1w**, **4a**–**4c**, **4e**–**4g** & **4i**–**4l**) and 3-formyl benzofuran (**4d**) are known or commercially available compounds, and commercially unavailable compounds were synthesized according to the literature reports.[1–4]

Synthetic procedure of starting materials for substrate scope(1a–1w)

Compound 1b: 1*H*-Pyrrolo[2,3-*c*]pyridine-3-carbaldehyde

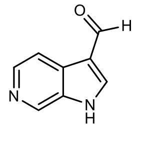

Compound **1b** was prepared from 1*H*-pyrrolo[2,3-*c*]pyridine following the general azaindole formylation procedure from the literature.[1] NMR matched with the data reported in the literature.[1]

Compound 1c: 1*H*-Pyrrolo[3,2-*c*]pyridine-3-carbaldehyde

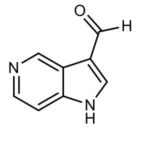

Compound **1c** was prepared from 1*H*-pyrrolo[3,2-*c*]pyridine following the general azaindole formylation procedure from the literature.[1] NMR matched with the data reported in the literature.[1]

Compound 1d: 1*H*-Pyrrolo[3,2-*b*]pyridine-3-carbaldehyde

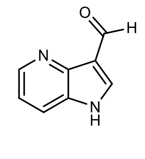

Compound **1d** was prepared from 1*H*-pyrrolo[3,2-*b*]pyridine following the general azaindole formylation procedure from the literature.[1] NMR matched with the data reported in the literature.[1]

Compound 1f: 7-Bromo-1*H*-indole-3-carbaldehyde

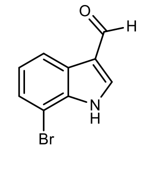

Compound **1f** was prepared from 7-bromo-1*H*-indole following the general indole formylation procedure from the literature.[1] NMR matched with the data reported in the literature.[1]

Compound 1j: 7-Nitro-1*H*-indole-3-carbaldehyde

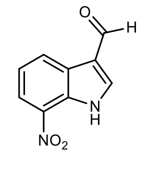

Compound **1j** was prepared from 7-nitro-1*H*-indole following the general indole formylation procedure from the literature.[1] NMR matched with the data reported in the literature.[1]

Compound 1k: 6-Nitro-1*H*-indole-3-carbaldehyde

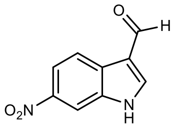

Compound **1k** was prepared from 6-nitro-1*H*-indole following the general indole formylation procedure from the literature.[1] NMR matched with the data reported in the literature.[1]

Compound 1l: 5-Nitro-1*H*-indole-3-carbaldehyde

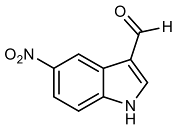

Compound **1l** was prepared from 5-nitro-1*H*-indole following the general indole formylation procedure from the literature.[1] NMR matched with the data reported in the literature.[1]

Compound 1n: 7-Methoxy-1*H*-indole-3-carbaldehyde

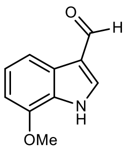

Compound **1n** was prepared from 7-methoxy-1*H*-indole following the general indole formylation procedure from the literature.[1] NMR matched with the data reported in the literature.[1]

Compound 1p: 5-Methoxy-1*H*-indole-3-carbaldehyde

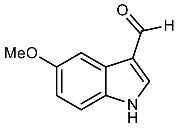

Compound **1p** was prepared from 5-methoxy-1*H*-indole following the general indole formylation procedure from the literature. NMR matched with the data reported in the literature.[1]

Compound 1q: 4-Methoxy-1*H*-indole-3-carbaldehyde

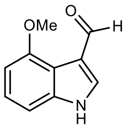

Compound **1q** was prepared from 4-methoxy-1*H*-indole following the general indole formylation procedure from the literature. NMR matched with the data reported in the literature.[1]

Compound 1r: 1-(Phenylsulfonyl)-1*H*-pyrrolo[2,3-*b*]pyridine-3-carbaldehyde

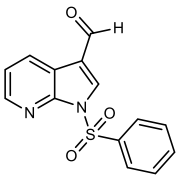

Compound **1r** was prepared from **1a** following the procedure from the literature.[1] NMR matched with the data reported in the literature.[1]

Compound 1s: 1-Benzyl-1*H*-pyrrolo[2,3-*b*]pyridine-3-carbaldehyde

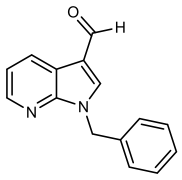

Compound **1s** was prepared from **1a** following the procedure from the literature.[1] NMR matched with the data reported in the literature.[1]

Compound 1t: 1-(4-Methoxybenzyl)-1*H*-pyrrolo[2,3-*b*]pyridine-3-carbaldehyde

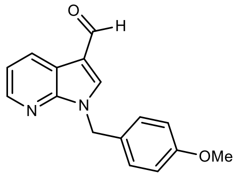

Compound **1t** was prepared from **1a** following the procedure from the literature.[1] NMR matched with the data reported in the literature.[1]

Compound 1u: 1-(4-Nitrobenzyl)-1*H*-pyrrolo[2,3-*b*]pyridine-3-carbaldehyde

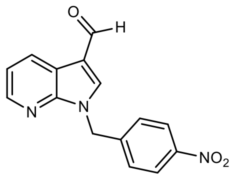

To the mixture of compound **1a** (731 mg, 5.0 mmol) in 5 mL of dry *N*,*N*-dimethylformamide (DMF) was added sodium hydride (NaH, 60% dispersion in mineral oil, 240 mg, 6.0 mmol) at 0 °C, and the mixture was stirred for 30 min. To the reaction mixture, 4-nitrobenzyl bromide (1.9 g, 5.5 mmol) was added portion-wise, and the temperature was slowly warmed to r.t. The reaction mixture was stirred for 1 h, and the reaction completion was monitored with analytical thin-layer chromatography (TLC) and LC-MS. The reaction mixture was poured into iced water to form fine precipitates. Precipitates were collected through sintered funnel, washed with distilled water and hot hexane, and dried *in vacuo* to provide 1-(4-nitrobenzyl)-1*H*-pyrrolo[2,3-*b*]pyridine-3-carbaldehyde (**1u**, 757 mg, 54%) as beige powder.

^{1}H NMR (400 MHz, DMSO-d_6): δ 9.97 (s, 1H), 8.71 (s, 1H), 8.47 (dd, J = 7.80, 1.62 Hz, 1H), 8.42 (dd, J = 4.71, 1.64 Hz, 1H), 8.25–8.16 (m, 2H), 7.58–7.51 (m, 2H), 7.36 (dd, J = 7.83, 4.73 Hz, 1H), 5.76 (s, 2H); ^{13}C NMR (101 MHz, DMSO-d_6): δ 185.36, 185.26, 147.89, 146.99, 145.07, 144.98, 144.71, 140.80, 140.63, 129.84, 129.73, 128.64, 128.44, 123.87, 119.24, 119.02, 116.95, 115.95, 47.42; LRMS (ESI): *m/z* calcd for $C_{15}H_{12}N_3O_3^+$ $[M+H]^+$: 282.09; Found: 282.1.

Compound 1v: 1-(Pyrimidin-2-yl)-1*H*-pyrrolo[2,3-*b*]pyridine-3-carbaldehyde

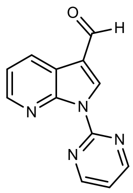

Compound **1v** was prepared from **1a** following the procedure from the literature.[1] NMR matched with the data reported in the literature.[1]

Compound 1w: 1-Ethyl-1*H*-pyrrolo[2,3-*b*]pyridine-3-carbaldehyde

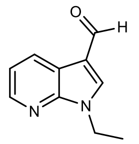

To the mixture of compound **1a** (731 mg, 5.0 mmol) in 5 mL of dry DMF was added sodium hydride (NaH, 60% dispersion in mineral oil, 240 mg, 6.0 mmol) at 0 °C, and the mixture was stirred for 30 min. Ethyl iodide (0.44 mL, 5.5 mmol) was dropwisely added to the reaction mixture, and the temperature was slowly warmed to r.t. The reaction mixture was stirred for 1 h, and the reaction completion was monitored with analytical thin-layer chromatography (TLC) and LC-MS. The reaction mixture was poured into iced water to form fine precipitates. Precipitates were collected through sintered funnel, washed with distilled water and hot hexane, and dried *in vacuo* to provide 1-ethyl-1*H*-pyrrolo[2,3-*b*]pyridine-3-carbaldehyde (**1w**, 712 mg, 82%) as orange solid.

^{1}H NMR (400 MHz, DMSO-d_6) δ 9.94 (d, J = 1.31 Hz, 1H), 8.60 (d, J = 1.31 Hz, 1H), 8.47 – 8.41 (m, 2H), 7.38 – 7.30 (m, 1H), 4.40 (qd, J = 7.28, 1.37 Hz, 2H), 1.47 (td, J = 7.25, 1.30 Hz, 3H); ^{13}C NMR (101 MHz, DMSO-d_6) δ 184.90, 147.84, 144.57, 140.23, 129.49, 118.75, 117.01, 115.30, 39.73, 15.09; LRMS (ESI): *m/z* calcd for C10H11N2O+ $[M+H]^+$: 175.09; Found: 175.1.

Synthetic procedure of starting materials for applications and SAR study (4a–4l)

Compound 4a: 7*H*-[1,3]Dioxolo[4,5-*j*]pyrrolo[3,2,1-*de*]phenanthridine-4-carbaldehyde

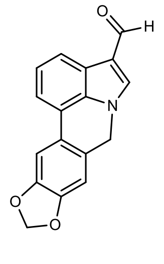

Compound **4a** was prepared following the procedure from the literature.[1] NMR matched with the data reported in the literature.[1]

Compound 4b: Ethyl 3-formyl-1*H*-indole-5-carboxylate

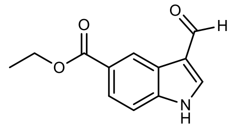

Compound **4b** was prepared following the procedure from the literature.[1] NMR matched with the data reported in the literature.[1]

Compound 4c: 5-Methyl-1*H*-pyrrolo[2,3-*b*]pyridine-3-carbaldehyde

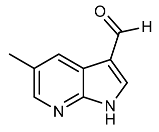

Compound **4c** was prepared from 5-methyl-1*H*-pyrrolo[2,3-*b*]pyridine following the azaindole formylation procedure from the literature.[1]

^{1}H NMR (500 MHz, DMSO-d_6): δ 12.57 (s, 1H), 9.90 (s, 1H), 8.41 (s, 1H), 8.30–8.18 (m, 2H), 2.42 (s, 3H); ^{13}C NMR (126 MHz, DMSO-d_6): δ 185.27, 147.96, 145.54, 138.74, 129.01, 127.33, 116.31, 116.20, 18.04; LRMS (ESI): *m/z* calcd for $C_9H_9N_2O^+$ $[M+H]^+$: 161.07; Found: 161.0.

Compound 4d: Benzofuran-3-carbaldehyde

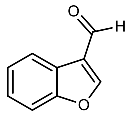

Compound **4a** was prepared following the procedure from the literature.[2] NMR matched with the data reported in the literature.[2]

Compound 4e: 5-Fluoro-1*H*-pyrrolo[2,3-*b*]pyridine-3-carbaldehyde

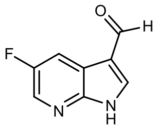

Compound **4e** was prepared from 5-fluoro-1*H*-pyrrolo[2,3-*b*]pyridine following the azaindole formylation procedure from the literature.[1] NMR matched with the data reported in the literature.

^{1}H NMR (500 MHz, DMSO-d_6): δ 12.88 (s, 1H), 9.94 (s, 1H), 8.57 (s, 1H), 8.39 (dd, J = 2.9, 1.8 Hz, 1H), 8.17 (dd, J = 8.9, 2.8 Hz, 1H); ^{13}C NMR (126 MHz, DMSO-d_6): δ 185.32, 156.41 (d, J = 243.2 Hz), 146.02, 140.27, 133.21 (d, J = 29.0 Hz), 116.76 (d, J = 7.8 Hz), 116.49 (d, J = 3.8 Hz), 114.70 (d, J = 21.3 Hz); LRMS (ESI): *m/z* calcd for $C_8H_6FN_2O^+$ [M+H]$^+$: 165.05; Found: 165.1.

Compound 4f: 6-Bromo-1*H*-pyrrolo[2,3-*b*]pyridine-3-carbaldehyde

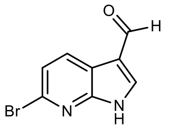

Compound **4f** was prepared from 6-bromo-1*H*-pyrrolo[2,3-*b*]pyridine following the azaindole formylation procedure from the literature.[1]

^{1}H NMR (500 MHz, DMSO-d_6): δ 12.93 (s, 1H), 9.95 (s, 1H), 8.52 (s, 1H), 8.34 (d, J = 8.1 Hz, 1H), 7.48 (d, J = 8.2 Hz, 1H); ^{13}C NMR (126 MHz, DMSO-d_6): δ 185.55, 148.68, 138.84, 135.48, 132.00, 121.89, 116.63, 115.54; LRMS (ESI): *m/z* calcd for $C_8H_6BrN_2O^+$ [M+H]$^+$: 224.97; Found: 224.9.

Compound 4g: 1-Benzyl-6-bromo-1*H*-indole-3-carbaldehyde

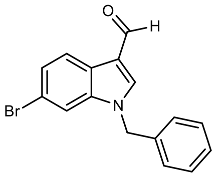

To the mixture of compound **1g** (448 mg, 2.0 mmol) in 4 mL of dry DMF was added sodium hydride (NaH, 60% dispersion in mineral oil, 96 mg, 2.4 mmol) at 0 °C, and the mixture was stirred for 30 min. Benzyl bromide (0.26 mL, 2.2 mmol) was dropwisely added to the reaction mixture, and the temperature was slowly warmed to r.t. The reaction mixture was stirred for 1 h, and the reaction completion was monitored with analytical thin-layer chromatography (TLC) and LC-MS. The reaction mixture was poured into iced water to form fine precipitates. Precipitates were collected through sintered funnel, washed with distilled water and hot hexane, and dried *in vacuo* to provide 1-benzyl-6-bromo-1*H*-indole-3-carbaldehyde (**4g**, 525 mg, 84%) as beige powder.

^{1}H NMR (500 MHz, DMSO-d_6): δ 9.96 (s, 1H), 8.50 (s, 1H), 8.06 (d, J = 8.4 Hz, 1H), 7.91 (d, J = 1.7 Hz, 1H), 7.40 (dd, J = 8.4, 1.7 Hz, 1H), 7.3–7.28 (m, 5H), 5.57 (s, 2H); ^{13}C NMR (126 MHz, DMSO-d_6): δ 184.86, 141.53, 137.86, 136.52, 128.79, 127.89, 127.33, 125.53, 123.75, 122.70, 117.32, 116.33, 114.25, 49.73; LRMS (ESI): *m/z* calcd for $C_{16}H_{13}BrNO^+$ [M+H]$^+$: 314.02; Found: 314.0.

Compound 4h: 6-Bromo-1-(4-methoxybenzyl)-1*H*-indole-3-carbaldehyde

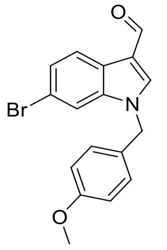

To the mixture of compound **1g** (448 mg, 2.0 mmol) in 4 mL of dry DMF was added sodium hydride (NaH, 60% dispersion in mineral oil, 96 mg, 2.4 mmol) at 0 °C, and the mixture was stirred for 30 min. 4-Methoxybenzyl chloride (1.16 mL, 2.2 mmol) was dropwisely added to the reaction mixture, and the temperature was slowly warmed to r.t. The reaction mixture was stirred for 1 h, and the reaction completion was monitored with analytical thin-layer chromatography (TLC) and LC-MS. The reaction mixture was poured into iced water to form fine precipitates. Precipitates were collected through sintered funnel, washed with distilled water and hot hexane, and dried *in vacuo* to provide 6-bromo-1-(4-methoxybenzyl)-1*H*-indole-3-carbaldehyde (**4h**, 610 mg, 89%) as beige powder.

^{1}H NMR (500 MHz, DMSO-d_6): δ 9.93 (s, 1H), 8.46 (s, 1H), 8.04 (d, J = 8.4 Hz, 1H), 7.93 (d, J = 1.7 Hz, 1H), 7.39 (dd, J = 8.4, 1.7 Hz, 1H), 7.35–7.24 (m, 2H), 7.00–6.86 (m, 2H), 5.47 (s, 2H), 3.72 (s, 3H); ^{13}C NMR (126 MHz, DMSO-d_6): δ 184.81, 158.94, 141.30, 137.75, 129.02, 128.29, 125.48, 123.80, 122.66, 117.20, 116.25, 114.31, 114.17, 55.07, 49.27; LRMS (ESI): *m/z* calcd for $C_{16}H_{13}BrNO^+$ $[M+H]^+$: 344.03; Found: 344.0.

Compound 4j: 6-Chloro-1*H*-indole-3-carbaldehyde

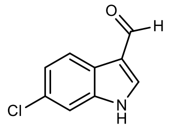

Compound **4j** was prepared from 6-chloro-1*H*-indole following the indole formylation procedure from the literature.[1] NMR matched with the data reported in the literature.[3]

Compound 4l: Methyl 3-formyl-1*H*-indole-6-carboxylate

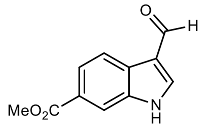

Compound **4l** was prepared from methyl 1*H*-indole-6-carboxylate following the indole formylation procedure from the literature.[1] NMR matched with the data reported in the literature.[4]

3.2. Synthetic procedure of acetylenes (2a–2p)

Most of the acetylene substrates are known or commercially available compounds. Commercially unavailable compounds were synthesized according to the literature reports.[5–14]

Compound 2a: 1-Phenylprop-2-yn-1-one

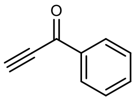

Compound **2a** was prepared following the procedure from the literature.[5]

Compound 2b: 2-Ethynyl-4,4-dimethyl-4*H*-benzo[*d*][1,3]oxazine

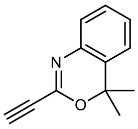

Compound **2b** was prepared following the procedure from the literature.[6]

Compound 2c: (Ethynylsulfonyl)benzene

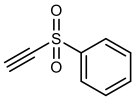

Compound **2c** was prepared following the procedure from the literature.[7]

Compound 2d: *N*-(*tert*-Butyl)ethynesulfonamide

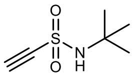

Compound **2d** was prepared following the procedure from the literature.[8]

Compound 2e: Ethyl ethynyl(methyl)phosphinate

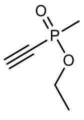

Compound **2e** was prepared from ethyl methylphosphonochloridate following the procedure from the literature.[9]

^{1}H NMR (400 MHz, $CDCl_3$): δ 4.30–4.06 (m, 2H), 2.95 (d, J = 10.3 Hz, 1H), 1.69 (d, J = 16.8 Hz, 3H), 1.38 (t, J = 7.1 Hz, 3H); ^{13}C NMR (101 MHz, $CDCl_3$): δ 88.32 (d, J = 35.1 Hz), 62.31 (d, J = 7.3 Hz), 17.58 (d, J = 122.9 Hz), 16.31 (d, J = 7.3 Hz).

Compound 2f: Diethyl ethynylphosphonate

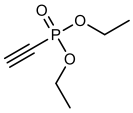

Compound **2f** was prepared following the procedure from the literature.[9]

Compound 2i: 2-Ethynylpyrimidine

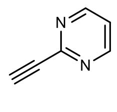

Compound **2i** was prepared following the procedure from the literature.[10]

Compound TIPS-2j: 4-((triisopropylsilyl)ethynyl)pyrimidine

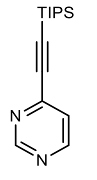

Compound **TIPS-2j** was prepared from 4-bromopyrimidine following the procedure from the literature.[10]

^{1}H NMR (400 MHz, $CDCl_3$): δ 9.19 (d, *J* = 1.6 Hz, 1H), 8.70 (d, *J* = 5.0 Hz, 1H), 7.40 (dd, *J* = 5.1, 1.6 Hz, 1H), 1.15 (d, *J* = 5.0 Hz, 21H); ^{13}C NMR (101 MHz, $CDCl_3$): δ 159.13, 157.05, 150.56, 124.11, 103.57, 98.42, 18.69, 11.24; LRMS (ESI): *m/z* calcd for $C_{15}H_{25}N_2Si^+$ $[M+H]^+$: 261.18; Found: 261.1.

Compound 2j: 4-Ethynylpyrimidine

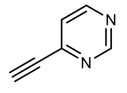

Compound **2j** was prepared from **TIPS-2j** following the procedure from the literature.[10]

^{1}H NMR (400 MHz, $CDCl_3$): δ 9.22 (d, *J* = 1.5 Hz, 1H), 8.76 (dd, *J* = 5.1, 1.3 Hz, 1H), 7.45 (dd, *J* = 5.1, 1.5 Hz, 1H), 3.43 (d, *J* = 1.4 Hz, 1H); ^{13}C NMR (101 MHz, $CDCl_3$): δ 159.10, 157.30, 149.83, 124.03, 82.09, 80.70; LRMS (ESI): *m/z* calcd for $C_6H_5N_2^+$ $[M+H]^+$: 105.04; Found: 105.1.

Compound TIPS-2j': 4-((triisopropylsilyl)ethynyl)pyrimidin-2-amine

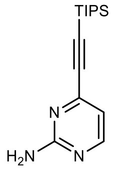

Compound **TIPS-2j'** was prepared from 2-amino-4-bromopyrimidine following the procedure from the literature.[10]

^{1}H NMR (400 MHz, $CDCl_3$): δ 8.25 (d, *J* = 4.9 Hz, 1H), 6.73 (d, *J* = 5.0 Hz, 1H), 5.25 (s, 2H), 1.13 (d, *J* = 4.4 Hz, 21H); ^{13}C NMR (101 MHz, $CDCl_3$): δ 163.07, 158.57, 151.45, 114.73, 104.19, 96.08, 18.74, 11.30; LRMS (ESI): *m/z* calcd for $[M+H]^+$: 276.19; Found: 276.1.

Compound 2j': 4-Ethynylpyrimidin-2-amine

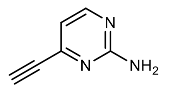

Compound **2j'** was prepared from **TIPS-2j'** following the procedure from the literature.[10]

^{1}H NMR (400 MHz, $CDCl_3$): δ 8.30 (dd, *J* = 4.9, 1.5 Hz, 1H), 6.75 (dd, *J* = 5.0, 1.5 Hz, 1H), 5.59 – 5.26 (m, 2H), 3.24 (s, 1H); ^{13}C NMR (101 MHz, $CDCl_3$): δ 163.01, 158.99, 150.59, 114.28, 81.21, 80.14; LRMS (ESI): *m/z* calcd for $C_6H_6N_3^+$ $[M+H]^+$: 120.06; Found: 120.1.

Compound 2k: 5-Ethynylpyrimidine

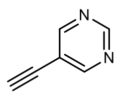

Compound **2k** was prepared from 5-bromopyrimidine following the procedure from the literature.[10]

Compound 2l: 2-Ethynylthiazole

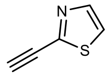

Compound **2l** was prepared from 2-bromothiazole following the procedure from the literature.[10]

Compound 2m: 2-Ethynylbenzo[*d*]thiazole

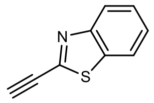

Compound **2m** was prepared from 2-mercaptobenzothiazole following the procedure from the literature.[11]

Compound 2n: 2-Ethynyl-1-methyl-1*H*-benzo[*d*]imidazole

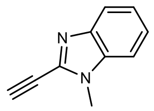

Compound **2n** prepared following the procedure from the literature.[12]

Compound 2o: *tert*-Butyl (4-oxohex-5-yn-1-yl)carbamate

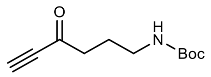

Compound **2o** was prepared following the procedure from the literature. NMR matched with the data reported in the literature.[13]

Synthesis of 3-((4-ethynylpyrimidin-2-yl)amino)-4-methyl-*N*-(3-(4-methyl-1*H*-imidazol-1-yl)-5-(trifluoromethyl)phenyl)benzamide (2p).

Compound 2p'': 3-Bromo-4-methyl-*N*-(3-(4-methyl-1*H*-imidazol-1-yl)-5-(trifluoromethyl)phenyl)benzamide

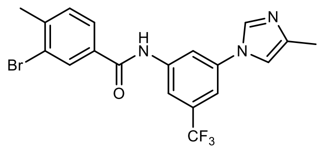

Compound **2p''** was prepared following the procedure from the literature.[14]

Compound 2p': 4-Methyl-*N*-(3-(4-methyl-1*H*-imidazol-1-yl)-5-(trifluoromethyl)phenyl)-3-((4-((triisopropylsilyl)ethynyl)pyrimidin-2-yl)amino)benzamide

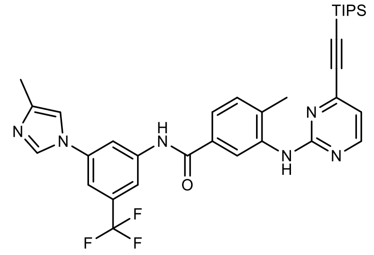

Compound **2p'** was prepared from **2p''** and **TIPS-2j'** following the Buchwald coupling procedure from the literature.[14]

^{1}H NMR (500 MHz, $CDCl_3$): δ 9.33 (s, 1H), 8.51 (d, *J* = 1.9 Hz, 1H), 8.36 (d, *J* = 5.0 Hz, 1H), 8.18 (d, *J* = 2.1 Hz, 1H), 7.84 (s, 1H), 7.76 (s, 1H), 7.57–7.52 (m, 1H), 7.28 (s, 1H), 7.22 (d, *J* = 7.8 Hz, 1H), 7.07 (s, 1H), 7.03 (s, 1H), 6.84 (d, *J* = 5.0 Hz, 1H), 2.30 (s, 3H), 2.24 (s, 3H), 1.09 (d, *J* = 5.0 Hz, 21H); ^{13}C NMR (126 MHz, $CDCl_3$): δ 166.64, 160.17, 158.23, 151.53, 141.02, 140.10, 138.38, 137.47, 134.59, 133.29, 132.96 (q, *J* = 33.1 Hz), 132.85, 131.06, 123.39 (q, *J* = 273.0 Hz), 122.96, 120.40, 115.99, 115.42, 115.12, 114.55, 112.52, 103.89, 97.48, 18.63, 18.33, 13.66, 11.22; LRMS (ESI): *m/z* calcd for $[M+H]^+$: 633.30; Found: 633.3.

Compound 2p: 3-((4-Ethynylpyrimidin-2-yl)amino)-4-methyl-*N*-(3-(4-methyl-1*H*-imidazol-1-yl)-5-(trifluoromethyl)phenyl)benzamide

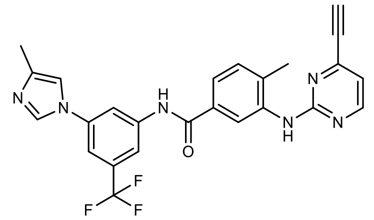

20 mL vial was charged with **2p'** (704 mg, 1.11 mmol) solution in dichloromethane (DCM, 9 mL). Tetrabutylammonium fluoride (1 M solution in THF, 1.22 mL, 1.1 equiv.) was added to the reaction mixture, and the reaction mixture was stirred at r.t. for 10 min. Reaction completion was monitored with TLC. Crude reaction mixture was directly purified through silica-gel flash column chromatography to provide 3-((4-ethynylpyrimidin-2-yl)amino)-4-methyl-*N*-(3-(4-methyl-1*H*-imidazol-1-yl)-5-(trifluoromethyl)phenyl)benzamide (**2p**, 296 mg, 56%) as off-white solid.

^{1}H NMR (400 MHz, $CDCl_3$): δ 9.51 (s, 1H), 8.56 (s, 1H), 8.35 (d, *J* = 4.9 Hz, 1H), 8.24 (s, 1H), 7.83 (s, 1H), 7.76 (s, 1H), 7.53 (d, *J* = 7.9 Hz, 1H), 7.32 (s, 1H), 7.27 (s, 1H), 7.18 (d, *J* = 7.9 Hz, 1H), 7.01 (s, 1H), 6.80 (d, *J* = 4.9 Hz, 1H), 3.25 (s, 1H), 2.27 (s, 3H), 2.21 (s, 3H); ^{13}C NMR (101 MHz, $CDCl_3$): δ 166.61, 160.10, 158.67, 150.40, 141.00, 139.98, 138.28, 137.30, 134.56, 133.32, 132.62 (q, *J* = 33.4 Hz), 132.51, 130.94, 123.35 (q, *J* = 272.8 Hz), 123.05, 120.34, 115.49, 115.19, 114.96 (d, *J* = 4.0 Hz), 114.48, 112.34, 81.02, 80.97, 18.20, 13.58; LRMS (ESI): *m/z* calcd for $[M+H]^+$: 477.16; Found: 477.2.

3.3. General synthetic procedure 1 for acetylene substrate scope (3aa–3an)

To a mixture of 3-formyl-7-azaindole (**1a**, 0.2 mmol), NH_4OAc (1.0 mmol. 5.0 equiv.), $Zn(OTf)_2$ (0.02 mmol, 10mol%) in EtOH (2.0 mL, 0.1 M) was added acetylene (**2a**–**2n**, 0.4 mmol, 2.0 equiv.). The reaction mixture was heated to 120 °C, and stirred for the indicated time. The reaction progress was monitored by thin-layer chromatography and LC-MS. Upon completion, the reaction mixture was poured into brine, and the organic layer was extracted thrice with DCM. The collected organic layer was dried on anhydrous Na_2SO_4(s), filtered through cotton, and concentrated *in vacuo*. The crude mixture was purified by silica-gel flash column chromatography to afford the desired products. Yields were obtained as an average of yields from 3 batches of each substrate scope.

Compound 3aa: (2'-Amino-[3,3'-bipyridin]-5-yl)(phenyl)methanone

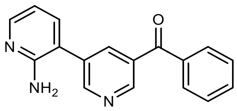

Following the general synthetic procedure 1, **1a** and **2a** were reacted for 16 h to provide **3aa** in 81% yield. ^{1}H NMR (500 MHz, DMSO-*d*$_6$): δ 8.85 (t, *J* = 1.9 Hz, 2H), 8.10 (t, *J* = 2.1 Hz, 1H), 8.01 (dd, *J* = 4.9, 1.8 Hz, 1H), 7.91–7.84 (m, 2H), 7.76–7.69 (m, 1H), 7.60 (t, *J* = 7.8 Hz, 2H), 7.46 (dd, *J* = 7.3, 1.8 Hz, 1H), 6.69 (dd, *J* = 7.3, 4.9 Hz, 1H), 5.91 (s, 2H); ^{13}C NMR (126 MHz, DMSO-*d*$_6$): δ 194.43, 156.95, 152.44, 148.52, 148.23, 138.14, 136.83, 136.39, 133.73, 133.34, 132.66, 129.89, 128.78, 116.05, 113.03. HRMS (ESI+): *m/z* calcd for $C_{17}H_{14}N_3O^+$ [M+H]$^+$: 276.11314; Found: 276.1129, Δ = 0.7243 ppm.

Compound 3ab: 5'-(4,4-Dimethyl-4*H*-benzo[*d*][1,3]oxazin-2-yl)-[3,3'-bipyridin]-2-amine

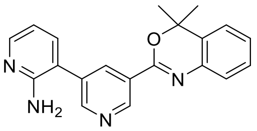

Following the general synthetic procedure 1, **1a** and **2b** were reacted for 16 h to provide **3ab** in 51% yield. ^{1}H NMR (500 MHz, DMSO-*d*$_6$): δ 9.19 (d, *J* = 2.0 Hz, 1H), 8.75 (d, *J* = 2.2 Hz, 1H), 8.39 (t, *J* = 2.1 Hz, 1H), 8.03 (dd, *J* = 4.9, 1.8 Hz, 1H), 7.43 (dd, *J* = 7.3, 1.9 Hz, 1H), 7.35 (ddd, *J* = 14.9, 7.4, 1.6 Hz, 2H), 7.27 (ddd, *J* = 14.3, 7.5, 1.4 Hz, 2H), 6.70 (dd, *J* = 7.3, 4.9 Hz, 1H), 5.87 (s, 2H), 1.70 (s, 6H); ^{13}C NMR (126 MHz, DMSO-*d*$_6$): δ 156.92, 154.36, 151.77, 148.08, 147.15, 138.15, 137.52, 134.76, 133.88, 131.34, 128.62, 128.17, 127.32, 124.66, 123.02, 116.41, 112.96, 78.93, 28.10. HRMS (ESI+): *m/z* calcd for $C_{20}H_{19}N_4O^+$ [M+H]$^+$: 331.15534; Found: 331.1553, Δ = 0 ppm.

Compound 3ac: 5'-(Phenylsulfonyl)-[3,3'-bipyridin]-2-amine

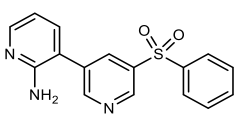

Following the general synthetic procedure 1, **1a** and **2c** were reacted for 16 h to provide **3ac** in 79% yield. ^{1}H NMR (500 MHz, DMSO-*d*$_6$): δ 9.09 (d, *J* = 2.2 Hz, 1H), 8.88 (d, *J* = 2.1 Hz, 1H), 8.37 (t, *J* = 2.2 Hz, 1H), 8.15–8.05 (m, 2H), 8.02 (dd, *J* = 4.9, 1.8 Hz, 1H), 7.78–7.70 (m, 1H), 7.66 (dd, *J* = 8.5, 7.1 Hz, 2H), 7.42 (dd, *J* = 7.4, 1.8 Hz, 1H), 6.68 (dd, *J* = 7.4, 4.9 Hz, 1H), 5.95 (s, 2H); ^{13}C NMR (126 MHz, DMSO-*d*$_6$): δ 156.91, 153.74, 148.58, 146.56, 140.52, 138.56, 137.64, 135.04, 134.83, 134.20, 129.87, 127.79, 115.31, 113.05; HRMS (ESI+): *m/z* calcd for $C_{16}H_{14}N_3O_2S^+$ [M+H]$^+$: 312.08013;

Found: 312.0802, Δ = 0.3204 ppm.

Compound 3ad: 2'-Amino-*N*-(*tert*-butyl)-[3,3'-bipyridine]-5-sulfonamide

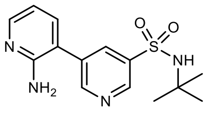

Following the general synthetic procedure 1, **1a** and **2d** were reacted for 16 h to provide **3ad** in 98% yield. ^{1}H NMR (500 MHz, DMSO-d_6): δ 8.92 (d, *J* = 2.2 Hz, 1H), 8.83 (d, *J* = 2.1 Hz, 1H), 8.20 (t, *J* = 2.2 Hz, 1H), 8.04 (dd, *J* = 4.9, 1.8 Hz, 1H), 7.78 (s, 1H), 7.40 (dd, *J* = 7.3, 1.8 Hz, 1H), 6.71 (dd, *J* = 7.3, 4.9 Hz, 1H), 5.88 (s, 2H), 1.15 (s, 9H); ^{13}C NMR (126 MHz, DMSO-d_6): δ 156.86, 152.09, 148.47, 145.33, 140.34, 138.33, 134.45, 133.69, 115.79, 113.20, 53.82, 29.84; HRMS (ESI+): *m/z* calcd for $C_{14}H_{19}N_4O_2S^+$ [M+H]$^+$: 307.12233; Found: 307.1225, Δ = 0.6512 ppm.

Compound 3ae: Ethyl (2'-amino-[3,3'-bipyridin]-5-yl)(methyl)phosphinate

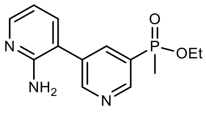

Following the general synthetic procedure 1, **1a** and **2e** were reacted for 16 h to provide **3ae** in 92% yield. ^{1}H NMR (500 MHz, CDCl$_3$): δ 8.95 (dd, *J* = 5.7, 1.9 Hz, 1H), 8.90 (t, *J* = 2.2 Hz, 1H), 8.24 (dt, *J* = 12.1, 2.1 Hz, 1H), 8.15 (dd, *J* = 5.0, 1.8 Hz, 1H), 7.39 (dd, *J* = 7.4, 1.8 Hz, 1H), 6.82 (dd, *J* = 7.4, 5.0 Hz, 1H), 4.58 (s, 2H), 4.24–3.91 (m, 2H), 1.76 (d, *J* = 14.7 Hz, 3H), 1.36 (t, *J* = 7.1 Hz, 3H); ^{13}C NMR (126 MHz, CDCl$_3$): δ 155.80, 152.95 (d, *J* = 1.9 Hz), 150.93 (d, *J* = 12.0 Hz), 148.93, 139.31 (d, *J* = 8.6 Hz), 138.62, 134.28 (d, *J* = 9.6 Hz), 128.71 (d, *J* = 123.3 Hz), 117.14, 115.08, 61.35 (d, *J* = 6.3 Hz), 16.63 (d, *J* = 6.5 Hz), 16.43 (d, *J* = 104.2 Hz); HRMS (ESI+): *m/z* calcd for $C_{13}H_{17}N_3O_2P^+$ [M+H]$^+$: 278.10529; Found: 278.1052, Δ = 0.3596 ppm.

Compound 3af: Diethyl (2'-amino-[3,3'-bipyridin]-5-yl)phosphonate

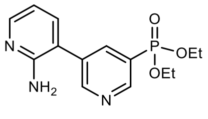

Following the general synthetic procedure 1, **1a** and **2f** were reacted for 16 h to provide **3af** in 67% yield. ^{1}H NMR (500 MHz, CDCl$_3$): δ 8.97 (dd, *J* = 6.3, 1.9 Hz, 1H), 8.88 (t, *J* = 2.3 Hz, 1H), 8.21 (dt, *J* = 13.7, 2.1 Hz, 1H), 8.18–8.11 (m, 1H), 7.39 (dd, *J* = 7.4, 1.8 Hz, 1H), 6.81 (dd, *J* = 7.4, 5.0 Hz, 1H), 4.57 (s, 2H), 4.32–4.11 (m, 4H), 1.38 (t, *J* = 7.1 Hz, 6H); ^{13}C NMR (126 MHz, CDCl$_3$): δ 155.83, 153.00 (d, *J* = 2.1 Hz), 151.33 (d, *J* = 12.1 Hz), 148.89, 139.49 (d, *J* = 8.4 Hz), 138.61, 134.00 (d, *J* = 12.0 Hz), 125.66 (d, *J* = 188.6 Hz), 117.17, 115.04, 62.92 (d, *J* = 5.7 Hz), 16.54 (d, *J* = 6.3 Hz); HRMS (ESI+): *m/z* calcd for $C_{14}H_{19}N_3O_3P^+$ [M+H]$^+$: 308.11586; Found: 308.1160, Δ = 0.3246 ppm.

Compound 3ag: [2,3':5',3''-Terpyridin]-2''-amine

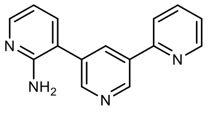

Following the general synthetic procedure 1, **1a** and **2g** were reacted for 72 h to provide **3ag** in 70% yield. ^{1}H NMR (500 MHz, DMSO-d_6): δ 9.25 (d, *J* = 2.1 Hz, 1H), 8.77–8.70 (m, 1H), 8.67 (d, *J* = 2.1 Hz, 1H), 8.47 (t, *J* = 2.2 Hz, 1H), 8.14 (dd, *J* = 7.9, 1.1 Hz, 1H), 8.02 (dd, *J* = 4.9, 1.8 Hz, 1H), 7.95 (td, *J* = 7.7, 1.8 Hz, 1H), 7.50–7.39 (m, 2H), 6.70 (dd, *J* = 7.3, 4.9 Hz, 1H), 5.84 (s, 2H); ^{13}C NMR (126 MHz, DMSO-d_6): δ 156.92, 153.72, 149.83, 149.47, 147.93, 146.47, 138.11, 137.46,

134.05, 133.94, 133.85, 123.35, 120.98, 116.85, 113.02; HRMS (ESI+): *m/z* calcd for $C_{15}H_{13}N_4^+$ $[M+H]^+$: 249.11348; Found: 249.1134, Δ = 0.4014 ppm.

Compound 3ai: 5'-(Pyrimidin-2-yl)-[3,3'-bipyridin]-2-amine

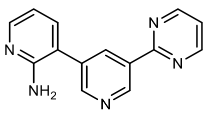

Following the general synthetic procedure 1, **1a** and **2i** were reacted for 16 h to provide **3ai** in 70% yield (57% when the scale is 1.46 g, 10 mmol). ^{1}H NMR (500 MHz, DMSO-d_6): δ 9.49–9.47 (m, 1H), 8.98 (dd, *J* = 4.8, 1.3 Hz, 2H), 8.74 (dd, *J* = 9.2, 2.2 Hz, 2H), 8.03 (dd, *J* = 4.9, 1.8 Hz, 1H), 7.55 (td, *J* = 4.9, 1.4 Hz, 1H), 7.46 (dd, *J* = 7.4, 1.8 Hz, 1H), 6.71 (dd, *J* = 7.3, 4.9 Hz, 1H), 5.86 (s, 2H); ^{13}C NMR (126 MHz, DMSO-d_6): δ 161.76, 157.98, 156.94, 151.09, 148.06, 147.46, 138.01, 134.88, 134.08, 132.53, 120.66, 116.59, 113.07; HRMS (ESI+): *m/z* calcd for $C_{14}H_{12}N_5^+$ $[M+H]^+$: 250.10873; Found: 250.1087, Δ = 0 ppm.

Compound 3aj: 5'-(Pyrimidin-4-yl)-[3,3'-bipyridin]-2-amine

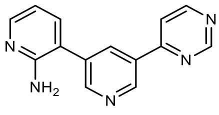

Following the general synthetic procedure 1, **1a** and **2j** were reacted for 16 h to provide **3aj** in 86% yield. ^{1}H NMR (500 MHz, DMSO-d_6): δ 9.35 (d, *J* = 2.2 Hz, 1H), 9.32 (d, *J* = 1.4 Hz, 1H), 8.94 (d, *J* = 5.4 Hz, 1H), 8.77 (d, *J* = 2.1 Hz, 1H), 8.60 (t, *J* = 2.2 Hz, 1H), 8.28 (dd, *J* = 5.3, 1.5 Hz, 1H), 8.03 (dd, *J* = 5.0, 1.8 Hz, 1H), 7.47 (dd, *J* = 7.3, 1.8 Hz, 1H), 6.71 (dd, *J* = 7.3, 5.0 Hz, 1H), 5.88 (s, 2H); ^{13}C NMR (126 MHz, DMSO-d_6): δ 160.75, 158.87, 158.32, 156.93, 151.42, 148.12, 146.82, 138.17, 134.53, 134.29, 131.55, 117.91, 116.42, 112.99; HRMS (ESI+): *m/z* calcd for $C_{14}H_{12}N_5^+$ $[M+H]^+$: 250.10873; Found: 250.1087, Δ = 0 ppm.

Compound 3aj': 5'-(2-Aminopyrimidin-4-yl)-[3,3'-bipyridin]-2-amine

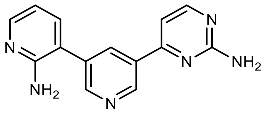

Following the general synthetic procedure 1, **1a** and **2j'** were reacted for 16 h to provide **3aj'** in 92% yield. ^{1}H NMR (500 MHz, DMSO-d_6): δ 9.22 (d, *J* = 2.1 Hz, 1H), 8.70 (d, *J* = 2.2 Hz, 1H), 8.47 (t, *J* = 2.2 Hz, 1H), 8.37 (d, *J* = 5.2 Hz, 1H), 8.02 (dd, *J* = 5.0, 1.8 Hz, 1H), 7.43 (dd, *J* = 7.3, 1.8 Hz, 1H), 7.30 (d, *J* = 5.1 Hz, 1H), 6.78 (s, 2H), 6.69 (dd, *J* = 7.3, 4.9 Hz, 1H), 5.82 (s, 2H); ^{13}C NMR (126 MHz, DMSO-d_6): δ 163.79, 161.37, 159.39, 156.90, 150.73, 148.00, 146.64, 138.07, 134.16, 134.04, 132.38, 116.66, 112.92, 106.12; HRMS (ESI+): *m/z* calcd for $C_{14}H_{13}N_6^+$ $[M+H]^+$: 265.11962; Found: 265.1194, Δ = 0.7544 ppm.

Compound 3al: 5'-(Thiazol-2-yl)-[3,3'-bipyridin]-2-amine

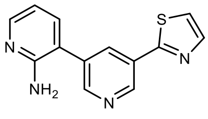

Following the general synthetic procedure 1, **1a** and **2l** were reacted for 72 h to provide **3al** in 77% yield. ^{1}H NMR (500 MHz, DMSO-d_6): δ 9.12 (d, *J* = 2.1 Hz, 1H), 8.68 (d, *J* = 2.1 Hz, 1H), 8.31 (t, *J* = 2.2 Hz, 1H), 8.02 (dd, *J* = 5.0, 2.4 Hz, 2H), 7.92 (d, *J* = 3.2 Hz, 1H),

7.46 (dd, J = 7.3, 1.9 Hz, 1H), 6.70 (dd, J = 7.3, 4.9 Hz, 1H), 5.90 (s, 2H); ^{13}C NMR (126 MHz, DMSO-d_6): δ 164.06, 156.93, 150.44, 148.17, 145.50, 144.14, 138.13, 134.47, 133.45, 128.95, 121.60, 116.20, 113.00; HRMS (ESI+): *m/z* calcd for $C_{13}H_{11}N_4S^+$ $[M+H]^+$: 255.06990; Found: 255.0699, Δ = 0 ppm.

Compound 3am: 5'-(Benzo[*d*]thiazol-2-yl)-[3,3'-bipyridin]-2-amine

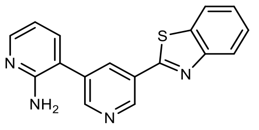

Following the general synthetic procedure 1, **1a** and **2m** were reacted for 72 h to provide **3am** in 78% yield. ^{1}H NMR (500 MHz, DMSO-d_6): δ 9.24 (d, J = 2.2 Hz, 1H), 8.78 (d, J = 2.1 Hz, 1H), 8.46 (t, J = 2.2 Hz, 1H), 8.22 (dd, J = 8.0, 1.2 Hz, 1H), 8.14 (d, J = 7.8 Hz, 1H), 8.04 (dd, J = 4.9, 1.9 Hz, 1H), 7.60 (ddd, J = 8.2, 7.1, 1.3 Hz, 1H), 7.56–7.47 (m, 2H), 6.72 (dd, J = 7.3, 4.9 Hz, 1H), 5.96 (s, 2H); ^{13}C NMR (126 MHz, DMSO-d_6): δ 164.54, 156.96, 153.35, 151.56, 148.28, 146.34, 138.19, 134.62, 134.61, 134.39, 128.84, 126.91, 125.96, 123.14, 122.56, 116.02, 113.04; HRMS (ESI+): *m/z* calcd for $C_{17}H_{13}N_4S^+$ $[M+H]^+$: 305.08555; Found: 305.0855, Δ = 0.3278 ppm.

Compound 3an: 5'-(1-Methyl-1*H*-benzo[*d*]imidazol-2-yl)-[3,3'-bipyridin]-2-amine

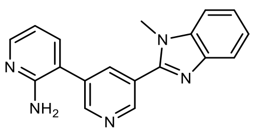

Following the general synthetic procedure 1, **1a** and **2n** were reacted for 72 h to provide **3an** in 46% yield. ^{1}H NMR (500 MHz, DMSO-d_6): δ 9.04 (d, J = 2.1 Hz, 1H), 8.76 (d, J = 2.2 Hz, 1H), 8.31 (t, J = 2.2 Hz, 1H), 8.03 (dd, J = 4.9, 1.8 Hz, 1H), 7.73 (d, J = 7.9 Hz, 1H), 7.67 (d, J = 8.0 Hz, 1H), 7.49 (dd, J = 7.3, 1.8 Hz, 1H), 7.36 – 7.31 (m, 1H), 7.30 – 7.26 (m, 1H), 6.72 (dd, J = 7.3, 4.9 Hz, 1H), 5.91 (s, 2H), 3.98 (s, 3H); ^{13}C NMR (126 MHz, DMSO-d_6): δ 157.04, 150.36, 149.94, 148.31, 148.12, 142.55, 138.19, 136.69, 136.36, 133.88, 126.27, 122.73, 122.17, 119.21, 116.50, 113.07, 110.74, 31.62; HRMS (ESI+): *m/z* calcd for $C_{18}H_{16}N_5^+$ $[M+H]^+$: 302.14003; Found: 302.1399, Δ = 0.3310 ppm.

3.4. General synthetic procedure 2 for (aza)indole substrate scope with 2e (3ae–3we)

To a mixture of 3-formyl-(aza)indole (**1a**–**1w**, 0.2 mmol), NH_4OAc (1.0 mmol. 5.0 equiv.), $Zn(OTf)_2$ (0.02 mmol, 10mol%) in EtOH (2.0 mL, 0.1 M) was added ethyl ethynyl(methyl)phosphinate (**2e**, 0.4 mmol, 2.0 equiv.). The reaction mixture was heated to 120 °C and stirred for the indicated time. The reaction progress was monitored by thin-layer chromatography and LC-MS. Upon completion, the reaction mixture was poured into brine, and the organic layer was extracted thrice with DCM. The collected organic layer was dried on anhydrous Na_2SO_4(s), filtered through cotton, and concentrated *in vacuo*. The crude mixture was purified by silica-gel flash column chromatography to afford the desired products (**3ae**–**3we**). Yields were obtained as an average of yields from 3 batches of each substrate scope.

Compound 3be: Ethyl (3'-amino-[3,4'-bipyridin]-5-yl)(methyl)phosphinate

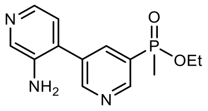

Following the general synthetic procedure 2, **1b** and **2e** were reacted for 16 h to provide **3be** in 64% yield. ^{1}H NMR (500 MHz, $CDCl_3$): δ 8.98 (dd, *J* = 5.7, 1.9 Hz, 1H), 8.92 (t, *J* = 2.2 Hz, 1H), 8.27 (dt, *J* = 12.0, 2.1 Hz, 1H), 8.23 (s, 1H), 8.11 (d, *J* = 4.6 Hz, 1H), 7.03 (d, *J* = 4.9 Hz, 1H), 4.23–4.11 (m, 1H), 4.04–3.90 (m, 3H), 1.77 (d, *J* = 14.8 Hz, 3H), 1.36 (t, *J* = 7.0 Hz, 3H); ^{13}C NMR (126 MHz, $CDCl_3$): δ 152.56 (d, *J* = 2.1 Hz), 151.29 (d, *J* = 12.0 Hz), 140.40, 139.98, 139.04 (d, *J* = 8.7 Hz), 138.81, 133.10 (d, *J* = 9.7 Hz), 128.67 (d, *J* = 123.5 Hz), 128.64, 124.11, 61.31 (d, *J* = 6.3 Hz), 16.56 (d, *J* = 6.5 Hz), 16.35 (d, *J* = 104.2 Hz); LRMS (ESI+): *m/z* calcd for $C_{13}H_{17}N_3O_2P^+$ $[M+H]^+$: 278.11; Found: 278.0.

Compound 3ce: Ethyl (4'-amino-[3,3'-bipyridin]-5-yl)(methyl)phosphinate

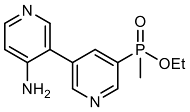

Following the general synthetic procedure 2, **1c** and **2e** were reacted for 16 h to provide **3ce** in 34% yield. ^{1}H NMR (500 MHz, $CDCl_3$): δ 8.95 (dd, *J* = 5.8, 1.9 Hz, 1H), 8.86 (t, *J* = 2.1 Hz, 1H), 8.26–8.18 (m, 2H), 8.13 (s, 1H), 6.70 (d, *J* = 5.7 Hz, 1H), 4.69 (s, 2H), 4.16 (dp, *J* = 10.1, 7.2 Hz, 1H), 4.02–3.93 (m, 1H), 1.77 (d, *J* = 14.7 Hz, 3H), 1.35 (t, *J* = 7.0 Hz, 3H); ^{13}C NMR (126 MHz, $CDCl_3$): δ 153.09 (d, *J* = 2.0 Hz), 150.97, 150.87, 150.70, 150.01 (d, *J* = 5.1 Hz), 139.48 (d, *J* = 8.6 Hz), 132.08 (d, *J* = 9.6 Hz), 128.59 (d, *J* = 123.6 Hz), 118.02, 110.11, 61.31 (d, *J* = 6.3 Hz), 16.53 (d, *J* = 6.5 Hz), 16.27 (d, *J* = 103.9 Hz); HRMS (ESI+): *m/z* calcd for $C_{13}H_{17}N_3O_2P^+$ $[M+H]^+$: 278.10529; Found: 278.1053, Δ = 0 ppm.

Compound 3de: Ethyl (3-amino-[2,3'-bipyridin]-5'-yl)(methyl)phosphinate

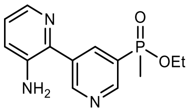

Following the general synthetic procedure 2, **1d** and **2e** were reacted for 16 h to provide **3de** in 90% yield. ^{1}H NMR (500 MHz, $CDCl_3$): δ 9.14 (t, *J* = 2.2 Hz, 1H), 8.95 (dd, *J* = 5.8, 2.0 Hz, 1H), 8.45 (dt, *J* = 12.1, 2.1 Hz, 1H), 8.14 (t, *J* = 3.0 Hz, 1H), 7.14 (d, *J* = 3.1 Hz, 2H), 4.20–4.10 (m, 1H), 4.07 (s, 2H), 3.94 (dp, *J* = 10.2, 7.2 Hz, 1H), 1.75 (d, *J* = 14.7 Hz, 3H), 1.34 (t, *J* = 7.0 Hz, 3H); ^{13}C NMR (126 MHz, $CDCl_3$): δ 152.82 (d, *J* = 1.9 Hz), 150.97 (d, *J* = 12.1 Hz), 140.89, 140.54, 140.24, 139.04 (d, *J* = 9.1 Hz), 134.71 (d, *J* = 9.6 Hz), 128.00 (d, *J* = 124.1 Hz), 124.29, 123.59, 61.17 (d, *J* = 6.1 Hz), 16.48 (d, *J* = 6.7 Hz), 16.22 (d, *J* = 104.1 Hz); LRMS (ESI+): *m/z* calcd for $C_{13}H_{17}N_3O_2P^+$ $[M+H]^+$: 278.11; Found: 278.1.

Compound 3ee: Ethyl (5-(2-aminophenyl)pyridin-3-yl)(methyl)phosphinate

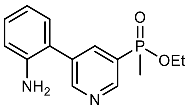

Following the general synthetic procedure 2, **1e** and **2e** were reacted for 16 h to provide **3ee** in 71% yield. ^{1}H NMR (500 MHz, $CDCl_3$): δ 8.93 (dd, *J* = 5.8, 2.0 Hz, 1H), 8.89 (t, *J* = 2.3 Hz, 1H), 8.22 (dt, *J* = 12.1, 2.0 Hz, 1H), 7.20 (td, *J* = 7.7, 1.7 Hz, 1H), 7.09 (dd, *J* = 7.6, 1.6 Hz, 1H), 6.85 (td, *J* = 7.4, 1.2 Hz, 1H), 6.81 (dd, *J* = 8.1, 1.2 Hz, 1H), 4.15 (dp, *J* = 10.2, 7.2 Hz, 1H), 4.00–3.79 (m, 3H), 1.75 (d, *J* = 14.7 Hz, 3H), 1.34 (t, *J* = 7.1 Hz, 3H); ^{13}C NMR (126 MHz, $CDCl_3$): δ 153.11 (d, *J* = 2.0 Hz), 150.07 (d, *J* = 12.1 Hz), 143.80, 139.16 (d, *J* = 8.7 Hz), 135.41 (d, *J* = 9.6 Hz), 130.45, 129.76, 127.95 (d, *J*

= 124.0 Hz), 122.25, 118.88, 116.06, 60.98 (d, *J* = 6.3 Hz), 16.37 (d, *J* = 6.6 Hz), 16.10 (d, *J* = 103.8 Hz); HRMS (ESI+): *m/z* calcd for $C_{14}H_{18}N_2O_2P^+$ $[M+H]^+$: 277.11004; Found: 277.1100, Δ = 0 ppm.

Compound 3fe: Ethyl (5-(2-amino-3-bromophenyl)pyridin-3-yl)(methyl)phosphinate

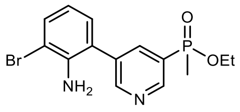

Following the general synthetic procedure 2, **1f** and **2e** were reacted for 16 h to provide **3fe** in 82% yield. ^{1}H NMR (500 MHz, $CDCl_3$): δ 8.94 (dd, *J* = 5.8, 2.0 Hz, 1H), 8.86 (t, *J* = 2.2 Hz, 1H), 8.20 (dt, *J* = 12.1, 2.1 Hz, 1H), 7.47 (dd, *J* = 8.1, 1.5 Hz, 1H), 7.03 (dd, *J* = 7.5, 1.5 Hz, 1H), 6.71 (t, *J* = 7.8 Hz, 1H), 4.25 (s, 2H), 4.21–4.11 (m, 1H), 3.97 (ddq, *J* = 10.1, 8.2, 7.0 Hz, 1H), 1.75 (d, *J* = 14.8 Hz, 3H), 1.35 (t, *J* = 7.1 Hz, 3H); ^{13}C NMR (126 MHz, $CDCl_3$): δ 152.96 (d, *J* = 2.1 Hz), 150.63 (d, *J* = 12.0 Hz), 141.60, 139.22 (d, *J* = 8.7 Hz), 134.93 (d, *J* = 9.6 Hz), 133.07, 129.72, 128.38 (d, *J* = 123.7 Hz), 123.52, 119.22, 110.23, 61.04 (d, *J* = 6.1 Hz), 16.42 (d, *J* = 6.4 Hz), 16.13 (d, *J* = 103.8 Hz); HRMS (ESI+): *m/z* calcd for $C_{14}H_{17}BrN_2O_2P$ $[M+H]^+$: 355.02056; Found: 355.0208, Δ = 0.5633 ppm.

Compound 3ge: Ethyl (5-(2-amino-4-bromophenyl)pyridin-3-yl)(methyl)phosphinate

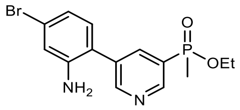

Following the general synthetic procedure 2, **1g** and **2e** were reacted for 16 h to provide **3ge** in 86% yield. ^{1}H NMR (500 MHz, $CDCl_3$): δ 8.92 (dd, *J* = 5.8, 1.9 Hz, 1H), 8.84 (t, *J* = 2.2 Hz, 1H), 8.19 (dt, *J* = 12.1, 2.1 Hz, 1H), 6.99–6.92 (m, 3H), 4.16 (dp, *J* = 10.1, 7.1 Hz, 1H), 4.06–3.88 (m, 3H), 1.75 (d, *J* = 14.7 Hz, 3H), 1.35 (t, *J* = 7.0 Hz, 3H); ^{13}C NMR (126 MHz, $CDCl_3$): δ 153.02 (d, *J* = 1.9 Hz), 150.46 (d, *J* = 12.1 Hz), 145.27, 139.22 (d, *J* = 8.7 Hz), 134.59 (d, *J* = 9.7 Hz), 131.82, 128.41 (d, *J* = 123.7 Hz), 123.58, 121.84, 121.27, 118.67, 61.19 (d, *J* = 6.3 Hz), 16.50 (d, *J* = 6.5 Hz), 16.27 (d, *J* = 103.6 Hz); LRMS (ESI+): *m/z* calcd for $C_{14}H_{17}BrN_2O_2P^+$ $[M+H]^+$: 355.02; Found: 355.0.

Compound 3he: Ethyl (5-(2-amino-5-bromophenyl)pyridin-3-yl)(methyl)phosphinate

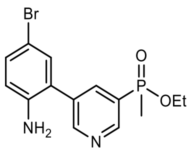

Following the general synthetic procedure 2, **1h** and **2e** were reacted for 16 h to provide **3he** in 84% yield. ^{1}H NMR (500 MHz, $CDCl_3$): δ 8.93 (dd, *J* = 5.7, 1.9 Hz, 1H), 8.86 (t, *J* = 2.1 Hz, 1H), 8.19 (dt, *J* = 12.0, 2.1 Hz, 1H), 7.28 (dd, *J* = 8.6, 2.3 Hz, 1H), 7.21 (d, *J* = 2.3 Hz, 1H), 6.70 (d, *J* = 8.6 Hz, 1H), 4.16 (dp, *J* = 10.0, 7.2 Hz, 1H), 4.01–3.93 (m, 1H), 3.89 (d, *J* = 34.3 Hz, 2H), 1.75 (d, *J* = 14.7 Hz, 3H), 1.35 (t, *J* = 7.0 Hz, 3H); ^{13}C NMR (126 MHz, $CDCl_3$): δ 153.01 (d, *J* = 2.0 Hz), 150.66 (d, *J* = 12.0 Hz), 143.04, 139.27 (d, *J* = 8.5 Hz), 134.25 (d, *J* = 9.6 Hz), 132.82, 132.51, 128.47 (d, *J* = 123.6 Hz), 124.20, 117.72, 110.48, 61.20 (d, *J* = 6.4 Hz), 16.50 (d, *J* = 6.4 Hz), 16.26 (d, *J* = 103.8 Hz); LRMS (ESI+): *m/z* calcd for $C_{14}H_{17}BrN_2O_2P^+$ $[M+H]^+$: 355.02; Found: 355.0.

Compound 3ie: Ethyl (5-(2-amino-6-bromophenyl)pyridin-3-yl)(methyl)phosphinate

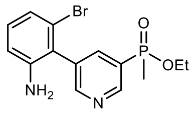

Following the general synthetic procedure 2, **1i** and **2e** were reacted for 16 h to provide **3ie** in 81% yield. ^{1}H NMR (500 MHz, $CDCl_3$): δ 8.99 (ddd, *J* = 15.0, 5.8, 1.9 Hz, 1H), 8.70 (t, *J* = 2.1 Hz, 1H), 8.04 (ddt, *J* = 21.8, 12.0, 2.1 Hz, 1H), 7.09–7.03 (m, 2H), 6.75 (dd, *J* = 7.0, 2.1 Hz, 1H), 4.21–4.07 (m, 1H), 4.07–3.90 (m, 1H), 3.72 (s, 2H), 1.75 (dd, *J* = 14.8, 11.1 Hz, 3H), 1.33 (dt, *J* = 12.3, 7.0 Hz, 3H); ^{13}C NMR (126 MHz, $CDCl_3$): δ 154.28 (d, *J* = 2.0 Hz), 151.19 (dd, *J* = 26.1, 12.0 Hz), 145.88 (d, *J* = 2.3 Hz), 141.05 (dd, *J* = 8.6, 3.3 Hz), 134.06 (dd, *J* = 10.0, 7.9 Hz), 130.68 (d, *J* = 2.0 Hz), 128.35 (dd, *J* = 123.5, 4.7 Hz), 124.44, 122.81 (d, *J* = 2.8 Hz), 122.39 (d, *J* = 2.3 Hz), 114.57, 61.15 (dd, *J* = 10.1, 6.3 Hz), 16.47 (d, *J* = 6.3 Hz), 16.93 – 15.38 (m); LRMS (ESI+): *m/z* calcd for $C_{14}H_{17}BrN_2O_2P^+$ $[M+H]^+$: 355.02; Found: 355.0.

Compound 3je: Ethyl (5-(2-amino-3-nitrophenyl)pyridin-3-yl)(methyl)phosphinate

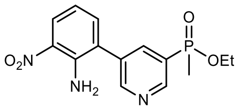

Following the general synthetic procedure 2, **1j** and **2e** were reacted for 16 h to provide **3je** in 89% yield. ^{1}H NMR (500 MHz, $CDCl_3$): δ 9.00 (dd, *J* = 5.8, 1.9 Hz, 1H), 8.85 (t, *J* = 2.2 Hz, 1H), 8.23 (dd, *J* = 8.7, 1.6 Hz, 1H), 8.20 (dt, *J* = 11.9, 2.1 Hz, 1H), 7.31 (dd, *J* = 7.2, 1.6 Hz, 1H), 6.83 (dd, *J* = 8.6, 7.2 Hz, 1H), 6.17 (s, 2H), 4.23 – 4.13 (m, 1H), 4.05–3.93 (m, 1H), 1.77 (d, *J* = 14.8 Hz, 3H), 1.36 (t, *J* = 7.1 Hz, 3H); ^{13}C NMR (126 MHz, $CDCl_3$): δ 153.25 (d, *J* = 2.0 Hz), 151.52 (d, *J* = 12.0 Hz), 142.51, 139.97 (d, *J* = 8.6 Hz), 137.03, 133.21 (d, *J* = 9.6 Hz), 133.08, 129.01 (d, *J* = 123.3 Hz), 127.13, 126.19, 116.65, 61.31 (d, *J* = 6.3 Hz), 16.56 (d, *J* = 6.5 Hz), 16.30 (d, *J* = 104.0 Hz); LRMS (ESI+): *m/z* calcd for $C_{14}H_{17}N_3O_4P^+$ $[M+H]^+$: 322.10; Found: 322.1.

Compound 3ke: Ethyl (5-(2-amino-4-nitrophenyl)pyridin-3-yl)(methyl)phosphinate

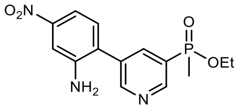

Following the general synthetic procedure 2, **1k** and **2e** were reacted for 16 h to provide **3ke** in 92% yield. ^{1}H NMR (500 MHz, $CDCl_3$): δ 8.98 (dd, *J* = 5.7, 1.9 Hz, 1H), 8.89 (t, *J* = 2.2 Hz, 1H), 8.24 (dt, *J* = 11.9, 2.1 Hz, 1H), 7.67 (dd, *J* = 8.2, 2.2 Hz, 1H), 7.65 (d, *J* = 2.1 Hz, 1H), 7.23 (d, *J* = 8.2 Hz, 1H), 4.18 (dp, *J* = 10.2, 7.2 Hz, 1H), 4.11 (s, 2H), 4.05–3.94 (m, 1H), 1.77 (d, *J* = 14.8 Hz, 3H), 1.36 (t, *J* = 7.0 Hz, 3H); ^{13}C NMR (126 MHz, $CDCl_3$): δ 152.82 (d, *J* = 2.1 Hz), 151.44 (d, *J* = 12.0 Hz), 149.28, 145.05, 139.37 (d, *J* = 8.7 Hz), 133.68 (d, *J* = 9.7 Hz), 131.54, 129.00 (d, *J* = 123.4 Hz), 128.24, 113.56, 110.38, 61.42 (d, *J* = 6.2 Hz), 16.64 (d, *J* = 6.4 Hz), 16.44 (d, *J* = 103.9 Hz); HRMS (ESI+): *m/z* calcd for $C_{14}H_{17}N_3O_4P^+$ $[M+H]^+$: 322.09512; Found: 322.0948, Δ = 0.9314 ppm.

Compound 3le: Ethyl (5-(2-amino-5-nitrophenyl)pyridin-3-yl)(methyl)phosphinate

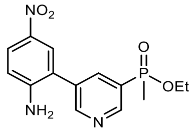

Following the general synthetic procedure 2, **1l** and **2e** were reacted for 16 h to provide **3le** in 81% yield. ^{1}H NMR (500 MHz, $CDCl_3$): δ 8.97 (d, *J* = 5.3 Hz, 1H), 8.88 (s, 1H), 8.23 (dt, *J* = 12.0, 2.0 Hz, 1H), 8.09 (dd, *J* = 9.0, 2.6 Hz, 1H), 8.02 (d, *J* = 2.6 Hz, 1H), 6.85 (d, *J* = 9.0 Hz, 1H), 4.90 (s, 2H), 4.18 (dp, *J* = 10.1, 7.1 Hz, 1H), 3.99 (ddq, *J* = 10.1, 8.1, 7.0 Hz, 1H), 1.79 (d, *J* = 14.7 Hz,

3H), 1.37 (t, *J* = 7.0 Hz, 3H); ^{13}C NMR (126 MHz, $CDCl_3$): δ 153.02 (d, *J* = 1.8 Hz), 151.18 (d, *J* = 12.0 Hz), 150.39, 139.52 (d, *J* = 8.7 Hz), 139.06, 133.26 (d), 128.78 (d, *J* = 124.1 Hz), 127.18, 126.30, 121.06, 114.77, 61.36 (d, *J* = 6.2 Hz), 16.53 (d, *J* = 6.6 Hz), 16.25 (d, *J* = 103.9 Hz); LRMS (ESI+): *m/z* calcd for $C_{14}H_{17}N_3O_4P^+$ $[M+H]^+$: 322.10; Found: 322.1.

Compound 3me: Ethyl (5-(2-amino-5-nitrophenyl)pyridin-3-yl)(methyl)phosphinate

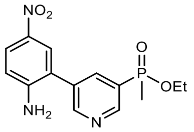

Following the general synthetic procedure 2, **1m** and **2e** were reacted for 16 h to provide **3me** in 89% yield. ^{1}H NMR (500 MHz, $CDCl_3$): δ 8.97 (d, *J* = 5.3 Hz, 1H), 8.88 (s, 1H), 8.23 (dt, *J* = 12.0, 2.0 Hz, 1H), 8.09 (dd, *J* = 9.0, 2.6 Hz, 1H), 8.02 (d, *J* = 2.6 Hz, 1H), 6.85 (d, *J* = 9.0 Hz, 1H), 4.90 (s, 2H), 4.18 (dp, *J* = 10.1, 7.1 Hz, 1H), 3.99 (ddq, *J* = 10.1, 8.1, 7.0 Hz, 1H), 1.79 (d, *J* = 14.7 Hz, 3H), 1.37 (t, *J* = 7.0 Hz, 3H); ^{13}C NMR (126 MHz, $CDCl_3$): δ 153.02 (d, *J* = 1.8 Hz), 151.18 (d, *J* = 12.0 Hz), 150.39, 139.52 (d, *J* = 8.7 Hz), 139.06, 133.26 (d), 128.78 (d, *J* = 124.1 Hz), 127.18, 126.30, 121.06, 114.77, 61.36 (d, *J* = 6.2 Hz), 16.53 (d, *J* = 6.6 Hz), 16.25 (d, *J* = 103.9 Hz); LRMS (ESI+): *m/z* calcd for $C_{14}H_{17}N_3O_4P^+$ $[M+H]^+$: 322.10; Found: 322.1.

Compound 3ne: Ethyl (5-(2-amino-3-methoxyphenyl)pyridin-3-yl)(methyl)phosphinate

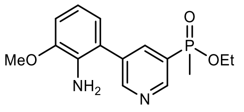

Following the general synthetic procedure 2, **1n** and **2e** were reacted for 16 h to provide **3ne** in 73% yield. ^{1}H NMR (500 MHz, $CDCl_3$): δ 8.93 (tt, *J* = 4.6, 2.2 Hz, 2H), 8.23 (dq, *J* = 12.1, 2.1 Hz, 1H), 6.84 (dtd, *J* = 18.1, 8.1, 2.1 Hz, 2H), 6.76 (dt, *J* = 7.9, 1.9 Hz, 1H), 4.22–4.10 (m, 1H), 4.01–3.87 (m, 6H), 1.74 (dd, *J* = 14.7, 1.9 Hz, 3H), 1.34 (td, *J* = 7.0, 1.9 Hz, 3H); ^{13}C NMR (126 MHz, $CDCl_3$): δ 153.19 (d, *J* = 2.1 Hz), 150.24 (d, *J* = 12.1 Hz), 147.41, 139.20 (d, *J* = 8.7 Hz), 135.34 (d, *J* = 9.6 Hz), 133.88, 128.04 (d, *J* = 123.9 Hz), 122.34, 122.30, 118.23, 110.35, 61.03 (d, *J* = 6.2 Hz), 55.74, 16.46 (d, *J* = 6.5 Hz), 16.21 (d, *J* = 103.9 Hz); LRMS (ESI+): *m/z* calcd for $C_{15}H_{20}N_2O_3P^+$ $[M+H]^+$: 307.12; Found: 307.1.

Compound 3oe: Ethyl (5-(2-amino-4-methoxyphenyl)pyridin-3-yl)(methyl)phosphinate

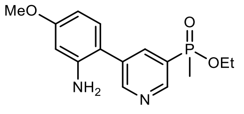

Following the general synthetic procedure 2, **1o** and **2e** were reacted for 16 h to provide **3oe** in 81% yield. ^{1}H NMR (500 MHz, DMSO-d_6): δ 8.80 – 8.73 (m, 2H), 8.07 (dt, *J* = 12.2, 2.1 Hz, 1H), 6.98 (d, *J* = 8.4 Hz, 1H), 6.39 (d, *J* = 2.5 Hz, 1H), 6.29 (dd, *J* = 8.4, 2.5 Hz, 1H), 5.08 (s, 2H), 3.99 (dp, *J* = 10.3, 7.2 Hz, 1H), 3.91–3.80 (m, 1H), 3.71 (s, 3H), 1.74 (d, *J* = 14.9 Hz, 3H), 1.22 (t, *J* = 7.0 Hz, 3H); ^{13}C NMR (126 MHz, $CDCl_3$): δ 161.22, 153.42 (d, *J* = 2.2 Hz), 149.90 (d, *J* = 12.0 Hz), 145.04, 139.37 (d, *J* = 8.7 Hz), 135.44 (d, *J* = 9.7 Hz), 131.77, 128.13 (d, *J* = 123.8 Hz), 115.65, 105.18, 101.56, 61.16 (d, *J* = 6.3 Hz), 55.35, 16.58 (d, *J* = 6.6 Hz), 16.37 (d, *J* = 103.8 Hz); LRMS (ESI+): *m/z* calcd for $C_{15}H_{20}N_2O_3P^+$ $[M+H]^+$: 307.12; Found: 307.1.

Compound 3pe: Ethyl (5-(2-amino-5-methoxyphenyl)pyridin-3-yl)(methyl)phosphinate

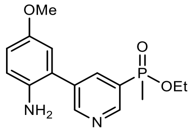

Following the general synthetic procedure 2, **1p** and **2e** were reacted for 16 h to provide **3pe** in 83% yield. ^{1}H NMR (500 MHz, DMSO-*d$_6$*) ; δ 8.91–8.76 (m, 2H), 8.16 (dt, *J* = 12.1, 2.1 Hz, 1H), 6.86–6.74 (m, 2H), 6.70 (d, *J* = 2.4 Hz, 1H), 4.60 (s, 2H), 4.00 (dp, *J* = 10.3, 7.2 Hz, 1H), 3.93–3.80 (m, 1H), 3.69 (s, 3H), 1.75 (d, *J* = 14.8 Hz, 3H), 1.22 (t, *J* = 7.0 Hz, 3H); ^{13}C NMR (126 MHz, DMSO-*d$_6$*); δ 152.50, 151.46, 149.49 (d, *J* = 12.0 Hz), 139.48, 138.62 (d, *J* = 8.9 Hz), 135.29 (d, *J* = 9.6 Hz), 127.91 (d, *J* = 122.6 Hz), 122.22, 117.19, 115.69, 115.25, 60.33 (d, *J* = 6.0 Hz), 55.43, 16.36 (d, *J* = 6.3 Hz), 15.24 (d, *J* = 101.5 Hz); LRMS (ESI+): *m/z* calcd for $C_{15}H_{20}N_2O_3P^+$ $[M+H]^+$: 307.12; Found: 307.1.

Compound 3qe: Ethyl (5-(2-amino-6-methoxyphenyl)pyridin-3-yl)(methyl)phosphinate

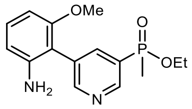

Following the general synthetic procedure 2, **1q** and **2e** were reacted for 16 h to provide **3qe** in 87% yield. ^{1}H NMR (500 MHz, $CDCl_3$): δ 8.93 (dd, *J* = 5.8, 2.0 Hz, 1H), 8.76 (t, *J* = 2.1 Hz, 1H), 8.09 (dt, *J* = 12.1, 2.0 Hz, 1H), 7.16 (t, *J* = 8.2 Hz, 1H), 6.44 (dd, *J* = 8.1, 0.9 Hz, 1H), 6.41 (dd, *J* = 8.2, 1.0 Hz, 1H), 4.20–4.10 (m, 1H), 3.95 (dddd, *J* = 15.1, 10.1, 7.5, 6.4 Hz, 1H), 3.68 (s, 3H), 3.58 (s, 2H), 1.73 (d, *J* = 14.7 Hz, 3H), 1.33 (t, *J* = 7.1 Hz, 3H); ^{13}C NMR (126 MHz, $CDCl_3$): δ 157.71, 155.06 (d, *J* = 2.1 Hz), 150.45 (d, *J* = 12.0 Hz), 145.28, 141.46 (d, *J* = 8.6 Hz), 131.34 (d, *J* = 10.0 Hz), 130.23, 127.96 (d, *J* = 124.1 Hz), 111.32, 108.97, 101.08, 61.06 (d, *J* = 6.3 Hz), 55.63, 16.54 (d, *J* = 6.5 Hz), 16.35 (d, *J* = 103.9 Hz); HRMS (ESI+): *m/z* calcd for $C_{15}H_{20}N_2O_3P^+$ $[M+H]^+$: 307.12061; Found: 307.1204, Δ = 0.6512 ppm.

Compound 3re: Ethyl methyl(2'-(phenylsulfonamido)-[3,3'-bipyridin]-5-yl)phosphinate

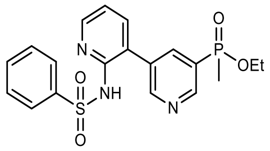

Following the general synthetic procedure 2, **1r** and **2e** were reacted for 16 h to provide **3re** in 89% yield. ^{1}H NMR (500 MHz, $CDCl_3$): δ 12.80 (s, 1H), 8.92 (d, *J* = 5.7 Hz, 1H), 8.83 (t, *J* = 2.1 Hz, 1H), 8.26 (dt, *J* = 12.3, 2.1 Hz, 1H), 7.95 (d, *J* = 7.5 Hz, 2H), 7.70 (d, *J* = 7.4 Hz, 2H), 7.58–7.47 (m, 3H), 6.78 (s, 1H), 4.12 (dp, *J* = 9.9, 7.3 Hz, 1H), 3.90 (dp, *J* = 9.7, 7.2 Hz, 1H), 1.68 (d, *J* = 14.8 Hz, 3H), 1.32 (t, *J* = 7.1 Hz, 3H); ^{13}C NMR (151 MHz, $CDCl_3$): δ 152.85, 152.25, 151.44 (d, *J* = 11.6 Hz), 142.97, 141.63, 139.88 (d, *J* = 9.4 Hz), 134.24, 132.33, 131.71, 129.04, 127.53 (d, *J* = 124.9 Hz), 126.13, 111.42, 61.32 (d, *J* = 6.4 Hz), 16.64, 16.27 (d, *J* = 98.4 Hz); HRMS (ESI+): *m/z* calcd for $C_{19}H_{21}N_3O_4PS^+$ $[M+H]^+$: 418.09849; Found: 418.0988, Δ = 0.7175 ppm.

Compound 3se: Ethyl (2'-(benzylamino)-[3,3'-bipyridin]-5-yl)(methyl)phosphinate

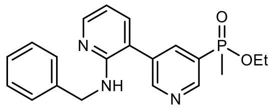

Following the general synthetic procedure 2, **1s** and **2e** were reacted for 16 h to provide **3se** in 74% yield. ^{1}H NMR (500 MHz, $CDCl_3$): δ 8.87 (dd, *J* = 5.8, 1.9 Hz, 1H), 8.83 (t, *J* = 2.1 Hz, 1H), 8.21 (dd, *J* = 5.0, 1.8 Hz, 1H), 8.18 (dt, *J* = 12.0, 2.1 Hz, 1H), 7.33–7.26 (m, 5H), 7.24–7.19 (m, 1H), 6.73 (dd, *J* = 7.3, 5.0 Hz, 1H), 4.83 (t, *J* = 5.5 Hz, 1H), 4.64 (d, *J* = 5.5 Hz, 2H), 4.14–4.00 (m, 1H), 3.86 (ddq, *J* = 10.1, 8.2, 7.0 Hz, 1H), 1.68 (d, *J* = 14.8 Hz, 3H), 1.28 (t, *J* = 7.1

Hz, 3H); ^{13}C NMR (126 MHz, $CDCl_3$): δ 155.02, 152.77 (d, J = 2.0 Hz), 150.59 (d, J = 12.0 Hz), 148.36, 139.30, 139.12 (d, J = 8.6 Hz), 137.64, 133.79 (d, J = 9.6 Hz), 128.36 (d, J = 123.2 Hz), 128.36, 127.43, 126.98, 117.12, 113.13, 60.85 (d, J = 6.3 Hz), 45.42, 16.32 (d, J = 2.0 Hz), 15.88 (d, J = 95.4 Hz); HRMS (ESI+): *m/z* calcd for $C_{20}H_{23}N_3O_2P^+$ $[M+H]^+$: 368.15224; Found: 368.1523, Δ = 0.2716 ppm.

Compound 3te: Ethyl (2'-((4-methoxybenzyl)amino)-[3,3'-bipyridin]-5-yl)(methyl)phosphinate

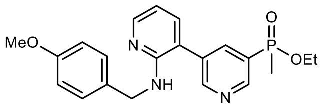

Following the general synthetic procedure 2, **1t** and **2e** were reacted for 16 h to provide **3te** in 99% yield. ^{1}H NMR (500 MHz, $CDCl_3$): δ 8.91 (dd, J = 5.8, 1.9 Hz, 1H), 8.85 (d, J = 2.3 Hz, 1H), 8.24 (dd, J = 5.1, 1.8 Hz, 1H), 8.17 (dt, J = 12.0, 2.1 Hz, 1H), 7.32 (dd, J = 7.3, 1.8 Hz, 1H), 7.24 (d, J = 8.4 Hz, 2H), 6.86–6.82 (m, 2H), 6.74 (dd, J = 7.3, 5.0 Hz, 1H), 4.56 (d, J = 5.1 Hz, 2H), 4.51 (t, J = 5.3 Hz, 1H), 4.11 (dp, J = 10.2, 7.2 Hz, 1H), 3.87 (dp, J = 10.2, 7.3 Hz, 1H), 3.77 (s, 3H), 1.70 (d, J = 14.8 Hz, 3H), 1.30 (t, J = 7.0 Hz, 3H); ^{13}C NMR (126 MHz, $CDCl_3$): δ 158.91, 155.20, 152.99 (d, J = 2.0 Hz), 150.86 (d, J = 12.0 Hz), 148.64, 139.43 (d, J = 8.7 Hz), 137.85, 134.03 (d, J = 9.5 Hz), 131.33, 129.07, 128.63 (d, J = 123.2 Hz), 117.32, 114.04, 113.34, 61.16 (d, J = 6.2 Hz), 55.29, 45.32, 16.47 (d, J = 6.4 Hz), 16.23 (d, J = 104.0 Hz); HRMS (ESI+): *m/z* calcd for $C_{21}H_{25}N_3O_3P^+$ $[M+H]^+$: 398.16281; Found: 398.1629, Δ = 0.2512 ppm.

Compound 3ue: Ethyl methyl(2'-((4-nitrobenzyl)amino)-[3,3'-bipyridin]-5-yl)phosphinate

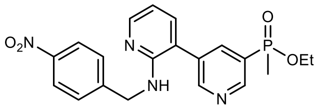

Following the general synthetic procedure 2, **1u** and **2e** were reacted for 16 h to provide **3ue** in 69% yield. ^{1}H NMR (500 MHz, $CDCl_3$): δ 8.89–8.86 (m, 2H), 8.22 (dt, J = 12.0, 2.1 Hz, 1H), 8.15 (dd, J = 5.0, 1.8 Hz, 1H), 8.09–8.05 (m, 2H), 7.50–7.45 (m, 2H), 7.36 (dd, J = 7.3, 1.8 Hz, 1H), 6.76 (dd, J = 7.3, 5.0 Hz, 1H), 5.33 (t, J = 6.0 Hz, 1H), 4.77 (d, J = 6.0 Hz, 2H), 4.13 (dp, J = 10.1, 7.2 Hz, 1H), 3.94 (ddq, J = 10.1, 8.2, 7.1 Hz, 1H), 1.74 (d, J = 14.7 Hz, 3H), 1.33 (t, J = 7.1 Hz, 3H); ^{13}C NMR (126 MHz, $CDCl_3$): δ 154.61, 152.77 (d, J = 2.0 Hz), 150.57 (d, J = 12.0 Hz), 148.32, 147.95, 146.60, 139.33 (d, J = 8.6 Hz), 137.95, 133.71 (d, J = 9.6 Hz), 128.55 (d, J = 123.7 Hz), 127.75, 123.41, 117.32, 113.70, 60.99 (d, J = 6.1 Hz), 44.53, 16.32 (d, J = 6.0 Hz), 15.93 (d, J = 103.9 Hz); HRMS (ESI+): *m/z* calcd for $C_{20}H_{22}N_4O_4P^+$ $[M+H]^+$: 413.13732; Found: 413.1375, Δ = 0.4841 ppm.

Compound 3ve: Ethyl methyl(2'-(pyrimidin-2-ylamino)-[3,3'-bipyridin]-5-yl)phosphinate

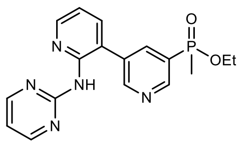

Following the general synthetic procedure 2, **1v** and **2e** were reacted for 16 h to provide **3ve** in 95% yield. ^{1}H NMR (500 MHz, $CDCl_3$): δ 9.90 (s, 1H), 8.93 (t, J = 2.2 Hz, 1H), 8.81 (dd, J = 5.8, 1.9 Hz, 1H), 8.59 (dd, J = 4.9, 1.8 Hz, 1H), 8.22–8.15 (m, 3H), 7.76 (dd, J = 7.6, 1.8 Hz, 1H), 7.29 (dd, J = 7.5, 4.9 Hz, 1H), 6.57 (t, J = 4.8 Hz, 1H), 4.06–3.98 (m, 1H), 3.80–3.65 (m, 1H), 1.61 (d, J = 14.7 Hz, 3H), 1.26 (t, J = 7.1 Hz, 3H); ^{13}C NMR (126 MHz, $CDCl_3$): δ 158.74, 157.44, 152.27 (d, J = 1.9 Hz), 150.15, 150.13 (d, J = 12.0 Hz), 148.46, 139.13, 137.35 (d, J = 9.1 Hz), 135.17 (d, J = 10.0 Hz), 127.01 (d, J = 123.6 Hz), 126.07, 120.24, 112.95, 60.71 (d, J = 6.2 Hz), 16.22 (d, J = 6.7

Hz), 15.92 (d, J = 104.4 Hz); HRMS (ESI+): *m/z* calcd for $C_{17}H_{19}N_5O_2P^+$ $[M+H]^+$: 356.12709; Found: 356.1271, Δ = 0 ppm.

Compound 3we: Ethyl (2'-(ethylamino)-[3,3'-bipyridin]-5-yl)(methyl)phosphinate

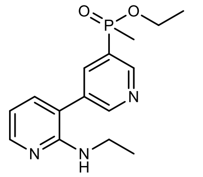

Following the general synthetic procedure 2, **1w** and **2e** were reacted for 16 h to provide **3we** in 63% yield. ^{1}H NMR (400 MHz, Chloroform-*d*): δ 8.96 (d, J = 5.46 Hz, 1H), 8.86 (d, J = 2.26 Hz, 1H), 8.21 (d, J = 4.96 Hz, 1H), 8.17 (dd, J = 12.09, 2.31 Hz, 1H), 7.31–7.24 (m, 1H), 6.69 (t, J = 6.04 Hz, 1H), 4.27 (t, J = 5.29 Hz, 1H), 4.21–4.14 (m, 1H), 3.97 (d, J = 8.51 Hz, 1H), 3.49–3.42 (m, 2H), 1.76 (dd, J = 14.65, 1.57 Hz, 3H), 1.36 (td, J = 7.07, 1.53 Hz, 3H), 1.23–1.14 (m, 3H); ^{13}C NMR (101 MHz, Chloroform-*d*): δ 155.55, 153.18, 150.94 (d, J = 12.24 Hz), 148.73, 139.59 (d, J = 8.56 Hz), 137.86, 134.35 (d, J = 9.75 Hz), 129.41, 128.19, 117.33, 112.93, 61.30, 36.59, 16.79 (d, J = 25.19 Hz), 16.24 (d, J = 71.71 Hz), 15.10; HRMS (ESI+): *m/z* calcd for C15H21N3O2P+ $[M+H]^+$: 306.13659; Found: 306.1365, Δ = 0.3267 ppm.

3.5. General synthetic procedure 3 for (aza)indole substrate scope with 2i (3ai–3wi)

To a mixture of 3-formyl-(aza)indole (**1a–1w**, 0.2 mmol), NH_4OAc (1.0 mmol. 5.0 equiv.), $Zn(OTf)_2$ (0.02 mmol, 10mol%) in EtOH (2.0 mL, 0.1 M) was added 2-ethynylpyrimidine (**2i**, 0.24 mmol, 1.2 equiv.). The reaction mixture was heated to 120 °C, and stirred for the indicated time. The reaction progress was monitored by thin-layer chromatography and LC-MS. Upon completion, the reaction mixture was poured into brine, and the organic layer was extracted thrice with DCM. The collected organic layer was dried on anhydrous Na_2SO_4(s), filtered through cotton, and concentrated *in vacuo*. The crude mixture was purified by silica-gel flash column chromatography to afford the desired products (**3ai**–**3wi**). Yields were obtained as an average of yields from 3 batches of each substrate scope.

Compound 3bi: 5-(Pyrimidin-2-yl)-[3,4'-bipyridin]-3'-amine

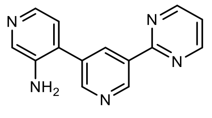

Following the general synthetic procedure 3, **1b** and **2i** were reacted for 16 h to provide **3bi** in 61% yield. ^{1}H NMR (500 MHz, DMSO-d_6): δ 9.52 (d, J = 2.0 Hz, 1H), 8.99 (d, J = 4.8 Hz, 2H), 8.86–8.74 (m, 2H), 8.15 (s, 1H), 7.88 (d, J = 4.9 Hz, 1H), 7.56 (t, J = 4.8 Hz, 1H), 7.12 (d, J = 4.8 Hz, 1H), 5.34 (s, 2H); ^{13}C NMR (126 MHz, DMSO-d_6): δ 161.65, 158.01, 150.75, 147.91, 141.97, 138.27, 137.85, 134.68, 133.11, 132.59, 127.24, 123.97, 120.71; LRMS (ESI+): *m/z* calcd for $C_{14}H_{12}N_5^+$ $[M+H]^+$: 250.11; Found: 250.0.

Compound 3ci: 5'-(Pyrimidin-2-yl)-[3,3'-bipyridin]-4-amine

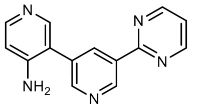

Following the general synthetic procedure 3, **1c** and **2i** were reacted for 16 h to provide **3ci** in 60% yield. ^{1}H NMR (500 MHz, DMSO-d_6): δ 9.50 (d, *J* = 2.0 Hz, 1H), 8.99 (d, *J* = 4.8 Hz, 2H), 8.73 (d, *J* = 2.2 Hz, 1H), 8.68 (t, *J* = 2.1 Hz, 1H), 8.05 (d, *J* = 5.6 Hz, 1H), 8.03 (s, 1H), 7.55 (t, *J* = 4.9 Hz, 1H), 6.69 (d, *J* = 5.6 Hz, 1H), 6.01 (s, 2H); ^{13}C NMR (126 MHz, DMSO-d_6): δ 161.75, 157.99, 151.71, 151.19, 149.81, 149.20, 147.57, 135.28, 132.61, 132.22, 120.66, 117.59, 109.62; LRMS (ESI+): *m/z* calcd for $C_{14}H_{12}N_5^+$ [M+H]$^+$: 250.11; Found: 250.1.

Compound 3di: 5'-(Pyrimidin-2-yl)-[2,3'-bipyridin]-3-amine

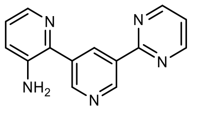

Following the general synthetic procedure 3, **1d** and **2i** were reacted for 16 h to provide **3di** in 46% yield. ^{1}H NMR (500 MHz, DMSO-d_6): δ 9.49 (d, *J* = 2.0 Hz, 1H), 9.01 (dd, *J* = 5.9, 2.2 Hz, 2H), 8.99 (d, *J* = 4.9 Hz, 2H), 7.98 (dd, *J* = 4.4, 1.5 Hz, 1H), 7.55 (t, *J* = 4.9 Hz, 1H), 7.20 (dd, *J* = 8.2, 1.5 Hz, 1H), 7.14 (dd, *J* = 8.2, 4.4 Hz, 1H), 5.36 (s, 2H); ^{13}C NMR (126 MHz, DMSO-d_6): δ 161.87, 157.98, 150.85, 147.53, 142.69, 139.42, 138.30, 134.94, 134.58, 132.29, 123.87, 122.92, 120.61; HRMS (ESI+): *m/z* calcd for $C_{14}H_{12}N_5^+$ [M+H]$^+$: 250.10873; Found: 250.1085, Δ = 0.7997 ppm.

Compound 3ei: 2-(5-(Pyrimidin-2-yl)pyridin-3-yl)aniline

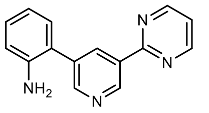

Following the general synthetic procedure 3, **1e** and **2i** were reacted for 16 h to provide **3ei** in 90% yield. ^{1}H NMR (500 MHz, DMSO-d_6): δ 9.46 (d, *J* = 2.0 Hz, 1H), 8.98 (d, *J* = 4.9 Hz, 2H), 8.74 (dd, *J* = 6.8, 2.2 Hz, 2H), 7.54 (t, *J* = 4.9 Hz, 1H), 7.12 (td, *J* = 7.6, 1.6 Hz, 1H), 7.09 (dd, *J* = 7.5, 1.6 Hz, 1H), 6.82 (dd, *J* = 8.1, 1.2 Hz, 1H), 6.69 (td, *J* = 7.4, 1.2 Hz, 1H), 5.02 (s, 2H); ^{13}C NMR (126 MHz, DMSO-d_6): δ 161.88, 157.96, 151.21, 146.98, 145.77, 135.41, 134.99, 132.45, 130.25, 129.15, 121.72, 120.59, 116.89, 115.61; HRMS (ESI+): *m/z* calcd for $C_{15}H_{13}N_4^+$ [M+H]$^+$: 249.11348; Found: 249.1132, Δ = 1.2043 ppm.

Compound 3fi: 2-Bromo-6-(5-(pyrimidin-2-yl)pyridin-3-yl)aniline

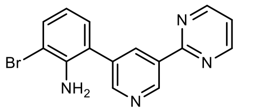

Following the general synthetic procedure 3, **1f** and **2i** were reacted for 16 h to provide **3fi** in 81% yield. ^{1}H NMR (500 MHz, DMSO-d_6): δ 9.50 (d, *J* = 2.1 Hz, 1H), 8.98 (d, *J* = 4.8 Hz, 2H), 8.76–8.67 (m, 2H), 7.55 (t, *J* = 4.9 Hz, 1H), 7.49 (dd, *J* = 8.0, 1.5 Hz, 1H), 7.13 (dd, *J* = 7.6, 1.4 Hz, 1H), 6.68 (t, *J* = 7.7 Hz, 1H), 5.07 (s, 2H); ^{13}C NMR (126 MHz, DMSO-d_6): δ 161.71, 157.99, 151.22, 147.60, 142.73, 135.20, 134.72, 132.56, 132.53, 130.02, 123.90, 120.67, 118.13, 109.11; LRMS (ESI+): *m/z* calcd for $C_{15}H_{12}BrN_4^+$ [M+H]$^+$: 327.02; Found: 327.0.

Compound 3gi (SB2031): 5-Bromo-2-(5-(pyrimidin-2-yl)pyridin-3-yl)aniline

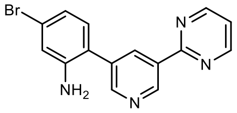

Following the general synthetic procedure 3, **1g** and **2i** were reacted for 16 h to provide **3gi** in 79% yield. ^{1}H NMR (500 MHz, DMSO-d_6): δ 9.47 (d, *J* = 2.0 Hz, 1H), 8.98 (d, *J* = 4.9 Hz, 2H), 8.71 (d, *J* = 2.2 Hz, 1H), 8.69 (t, *J* = 2.2 Hz, 1H), 7.55 (t, *J* = 4.9 Hz, 1H), 7.03 (d, *J* = 8.1 Hz, 1H), 7.00 (d, *J* = 2.0 Hz, 1H), 6.81 (dd, *J* = 8.1, 2.0 Hz, 1H), 5.35 (s, 2H); ^{13}C NMR (126 MHz, $CDCl_3$): δ 162.89, 157.61, 151.59, 148.95, 145.28, 135.95, 134.24, 133.37, 132.05, 123.25, 122.41, 121.94, 120.10, 118.52; HRMS (ESI+): *m/z* calcd for $C_{15}H_{12}BrN_4^+$ [M+H]$^+$: 327.02399; Found: 327.0243, Δ = 0.9174 ppm.

Compound 3hi: 4-Bromo-2-(5-(pyrimidin-2-yl)pyridin-3-yl)aniline

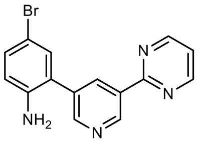

Following the general synthetic procedure 3, **1h** and **2i** were reacted for 16 h to provide **3hi** in 78% yield. ^{1}H NMR (500 MHz, DMSO-d_6): δ 9.48 (d, *J* = 2.1 Hz, 1H), 8.98 (d, *J* = 4.9 Hz, 2H), 8.74 (d, *J* = 2.2 Hz, 1H), 8.70 (t, *J* = 2.2 Hz, 1H), 7.55 (t, *J* = 4.9 Hz, 1H), 7.26 (dd, *J* = 8.6, 2.4 Hz, 1H), 7.23 (d, *J* = 2.4 Hz, 1H), 6.77 (d, *J* = 8.6 Hz, 1H), 5.24 (s, 2H); ^{13}C NMR (126 MHz, DMSO-d_6): δ 161.77, 157.99, 151.14, 147.44, 145.39, 135.09, 134.04, 132.52, 132.09, 131.59, 123.70, 120.66, 117.43, 107.16; LRMS (ESI+): *m/z* calcd for $C_{15}H_{12}BrN_4^+$ [M+H]$^+$: 327.02; Found: 327.0.

Compound 3ii: 3-Bromo-2-(5-(pyrimidin-2-yl)pyridin-3-yl)aniline

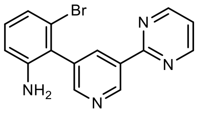

Following the general synthetic procedure 3, **1i** and **2i** were reacted for 16 h to provide **3ii** in 89% yield. ^{1}H NMR (500 MHz, DMSO-d_6): δ 9.53 (d, *J* = 2.0 Hz, 1H), 8.98 (d, *J* = 4.9 Hz, 2H), 8.53 (d, *J* = 2.2 Hz, 1H), 8.46 (t, *J* = 2.1 Hz, 1H), 7.54 (t, *J* = 4.8 Hz, 1H), 7.04 (t, *J* = 8.0 Hz, 1H), 6.91 (dd, *J* = 7.8, 1.1 Hz, 1H), 6.79 (dd, *J* = 8.2, 1.1 Hz, 1H), 5.06 (s, 2H); ^{13}C NMR (126 MHz, DMSO-d_6): δ 161.67, 158.01, 152.55, 148.19, 147.88, 136.79, 133.93, 132.71, 130.40, 123.71, 121.90, 120.65, 119.43, 114.08; LRMS (ESI+): *m/z* calcd for $C_{15}H_{12}BrN_4^+$ [M+H]$^+$: 327.02; Found: 327.0.

Compound 3ji: 2-Nitro-6-(5-(pyrimidin-2-yl)pyridin-3-yl)aniline

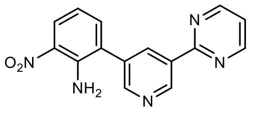

Following the general synthetic procedure 3, **1j** and **2i** were reacted for 16 h to provide **3ji** in 95% yield. ^{1}H NMR (500 MHz, DMSO-d_6): δ 9.56 (d, *J* = 2.0 Hz, 1H), 8.99 (d, *J* = 4.9 Hz, 2H), 8.74 (d, *J* = 2.2 Hz, 1H), 8.68 (t, *J* = 2.2 Hz, 1H), 8.13 (dd, *J* = 8.7, 1.6 Hz, 1H), 7.56 (t, *J* = 4.8 Hz, 1H), 7.47 (dd, *J* = 7.1, 1.6 Hz, 1H), 7.05 (s, 2H), 6.82 (dd, *J* = 8.7, 7.1 Hz, 1H); ^{13}C NMR (126 MHz, DMSO-d_6): δ 161.58, 158.03, 151.54, 148.25, 143.64, 137.36, 135.75, 133.07, 132.73, 131.78, 127.17, 126.12, 120.76, 115.77; LRMS (ESI+): *m/z* calcd for $C_{15}H_{12}N_5O_2^+$ [M+H]$^+$: 294.10; Found: 294.1.

Compound 3ki: 5-Nitro-2-(5-(pyrimidin-2-yl)pyridin-3-yl)aniline

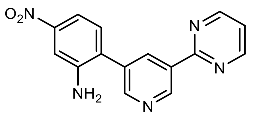

Following the general synthetic procedure 3, **1k** and **2i** were reacted for 16 h to provide **3ki** in 63% yield. ^{1}H NMR (500 MHz, DMSO-d_6): δ 9.53 (d, *J* = 2.0 Hz, 1H), 8.99 (d, *J* = 4.9 Hz, 2H), 8.78 (d, *J* = 2.3 Hz, 1H), 8.74 (t, *J* = 2.1 Hz, 1H), 7.67 (d, *J* = 2.3 Hz, 1H), 7.56 (t, *J* = 4.9 Hz, 1H), 7.47 (dd, *J* = 8.3, 2.4 Hz, 1H), 7.35 (d, *J* = 8.3 Hz, 1H), 5.76 (s, 2H); ^{13}C NMR (126 MHz, DMSO-d_6): δ 161.62, 158.02, 150.94, 148.28, 148.00, 147.35, 134.96, 133.58, 132.60, 131.55, 127.78, 120.73, 110.59, 108.93; LRMS (ESI+): *m/z* calcd for $C_{15}H_{12}N_5O_2^+$ $[M+H]^+$: 294.10; Found: 294.1.

Compound 3li: 4-Nitro-2-(5-(pyrimidin-2-yl)pyridin-3-yl)aniline

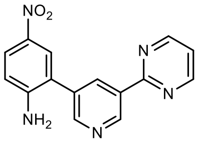

Following the general synthetic procedure 3, **1l** and **2i** were reacted for 16 h to provide **3li** in 80% yield. ^{1}H NMR (500 MHz, DMSO-d_6): δ 9.54 (d, *J* = 2.0 Hz, 1H), 8.99 (d, *J* = 4.9 Hz, 2H), 8.78 (d, *J* = 2.2 Hz, 1H), 8.69 (t, *J* = 2.2 Hz, 1H), 8.05 (dd, *J* = 9.1, 2.7 Hz, 1H), 7.95 (d, *J* = 2.7 Hz, 1H), 7.56 (t, *J* = 4.9 Hz, 1H), 6.86 (d, *J* = 9.1 Hz, 1H), 6.64 (s, 2H); ^{13}C NMR (126 MHz, DMSO-d_6): δ 161.65, 158.02, 151.27, 147.97, 136.35, 135.40, 133.11, 132.70, 127.09, 125.94, 120.72, 120.54, 114.23; HRMS (ESI+): *m/z* calcd for $C_{15}H_{12}N_5O_2^+$ $[M+H]^+$: 294.09855; Found: 294.0984, Δ = 0.6800 ppm.

Compound 3mi: 3-Nitro-2-(5-(pyrimidin-2-yl)pyridin-3-yl)aniline

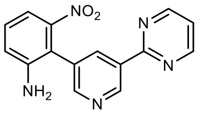

Following the general synthetic procedure 3, **1m** and **2i** were reacted for 16 h to provide **3mi** in 89% yield. ^{1}H NMR (500 MHz, DMSO-d_6): δ 9.53 (d, *J* = 2.1 Hz, 1H), 8.98 (d, *J* = 4.9 Hz, 2H), 8.53 (d, *J* = 2.2 Hz, 1H), 8.49 (t, *J* = 2.2 Hz, 1H), 7.55 (t, *J* = 4.8 Hz, 1H), 7.34 (t, *J* = 8.1 Hz, 1H), 7.17 (dd, *J* = 7.9, 1.1 Hz, 1H), 7.09 (dd, *J* = 8.2, 1.1 Hz, 1H), 5.42 (s, 2H); ^{13}C NMR (126 MHz, DMSO-d_6): δ 161.52, 158.02, 151.43, 148.31, 148.16, 135.93, 132.80, 130.24, 129.58, 120.72, 119.02, 114.05, 110.75; LRMS (ESI+): *m/z* calcd for $C_{15}H_{12}N_5O_2^+$ $[M+H]^+$: 294.10; Found: 294.1.

Compound 3ni: 2-Methoxy-6-(5-(pyrimidin-2-yl)pyridin-3-yl)aniline

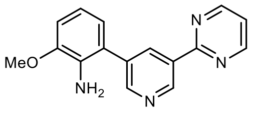

Following the general synthetic procedure 3, **1n** and **2i** were reacted for 16 h to provide **3ni** in 75% yield. ^{1}H NMR (500 MHz, DMSO-d_6): δ 9.46 (d, *J* = 2.0 Hz, 1H), 8.98 (d, *J* = 4.8 Hz, 2H), 8.75 (dd, *J* = 6.2, 2.2 Hz, 2H), 7.54 (t, *J* = 4.9 Hz, 1H), 6.93 (dd, *J* = 7.9, 1.5 Hz, 1H), 6.78 (dd, *J* = 7.8, 1.5 Hz, 1H), 6.72 (t, *J* = 7.8 Hz, 1H), 4.59 (s, 2H), 3.84 (s, 3H); ^{13}C NMR (126 MHz, DMSO-d_6): δ 161.85, 157.98, 151.22, 147.07, 146.90, 135.17, 135.00, 134.86, 132.47, 122.28, 121.95, 120.61, 116.85, 110.45, 55.70; HRMS (ESI+): *m/z* calcd for $C_{16}H_{15}N_4O^+$ $[M+H]^+$: 279.12404; Found: 279.1239, Δ = 0.3583 ppm.

Compound 3oi: 5-Methoxy-2-(5-(pyrimidin-2-yl)pyridin-3-yl)aniline

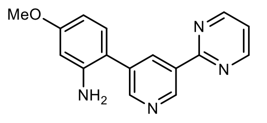

Following the general synthetic procedure 3, **1o** and **2i** were reacted for 16 h to provide **3oi** in 56% yield. ^{1}H NMR (500 MHz, DMSO-d_6): δ 9.41 (d, *J* = 2.0 Hz, 1H), 8.97 (d, *J* = 4.9 Hz, 2H), 8.70 (dd, *J* = 6.4, 2.2 Hz, 2H), 7.54 (t, *J* = 4.9 Hz, 1H), 7.02 (d, *J* = 8.3 Hz, 1H), 6.40 (d, *J* = 2.5 Hz, 1H), 6.30 (dd, *J* = 8.3, 2.5 Hz, 1H), 5.06 (s, 2H), 3.72 (s, 3H); ^{13}C NMR (126 MHz, DMSO-d_6): δ 161.96, 160.32, 157.95, 151.23, 147.03, 146.52, 135.29, 134.89, 132.40, 131.25, 120.55, 114.91, 103.22, 100.48, 54.80; LRMS (ESI+): *m/z* calcd for $C_{16}H_{15}N_4O^+$ $[M+H]^+$: 279.12; Found: 279.1.

Compound 3pi: 4-Methoxy-2-(5-(pyrimidin-2-yl)pyridin-3-yl)aniline

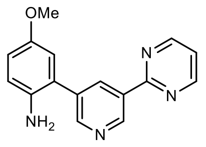

Following the general synthetic procedure 3, **1p** and **2i** were reacted for 16 h to provide **3pi** in 60% yield. ^{1}H NMR (500 MHz, DMSO-d_6): δ 9.45 (d, *J* = 2.0 Hz, 1H), 8.98 (d, *J* = 4.8 Hz, 2H), 8.76 (dd, *J* = 5.5, 2.2 Hz, 2H), 7.54 (t, *J* = 4.9 Hz, 1H), 6.78 (d, *J* = 1.9 Hz, 2H), 6.73 (t, *J* = 1.6 Hz, 1H), 4.58 (s, 2H), 3.69 (s, 3H); ^{13}C NMR (126 MHz, DMSO-d_6): δ 161.88, 157.98, 151.42, 151.27, 147.04, 139.45, 135.37, 135.02, 132.41, 122.62, 120.61, 117.07, 115.54, 115.04, 55.42; LRMS (ESI+): *m/z* calcd for $C_{16}H_{15}N_4O^+$ $[M+H]^+$: 279.12; Found: 279.1.

Compound 3qi: 3-Methoxy-2-(5-(pyrimidin-2-yl)pyridin-3-yl)aniline

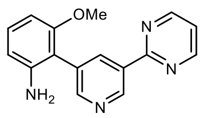

Following the general synthetic procedure 3, **1q** and **2i** were reacted for 16 h to provide **3qi** in 82% yield. ^{1}H NMR (500 MHz, DMSO-d_6): δ 9.43 (d, *J* = 2.1 Hz, 1H), 8.96 (d, *J* = 4.9 Hz, 2H), 8.51 (dd, *J* = 11.3, 2.1 Hz, 2H), 7.52 (t, *J* = 4.9 Hz, 1H), 7.06 (t, *J* = 8.2 Hz, 1H), 6.44 (d, *J* = 8.1 Hz, 1H), 6.34 (d, *J* = 8.2 Hz, 1H), 4.74 (s, 2H), 3.61 (s, 3H); ^{13}C NMR (126 MHz, DMSO-d_6): δ 161.96, 157.95, 157.26, 153.18, 147.04, 146.97, 137.16, 132.54, 131.26, 129.39, 120.50, 110.28, 108.47, 99.15, 55.35; LRMS (ESI+): *m/z* calcd for $C_{16}H_{15}N_4O^+$ $[M+H]^+$: 279.12; Found: 279.1.

Compound 3ri: *N*-(5'-(Pyrimidin-2-yl)-[3,3'-bipyridin]-2-yl)benzenesulfonamide

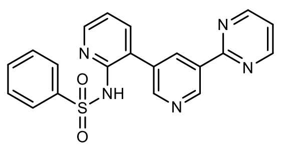

Following the general synthetic procedure 3, **1r** and **2i** were reacted for 16 h to provide **3ri** in 75% yield. ^{1}H NMR (500 MHz, $CDCl_3$): δ 12.78 (s, 1H), 9.60 (d, *J* = 2.0 Hz, 1H), 8.83 (t, *J* = 4.0 Hz, 3H), 8.77 (d, *J* = 2.3 Hz, 1H), 8.03 (d, *J* = 7.6 Hz, 2H), 7.75 (s, 1H), 7.69 (d, *J* = 7.2 Hz, 1H), 7.52 (t, *J* = 7.3 Hz, 1H), 7.47 (dd, *J* = 8.3, 6.8 Hz, 2H), 7.33–7.22 (m, 1H), 6.81 (s, 1H); ^{13}C NMR (151 MHz, $CDCl_3$): δ 162.71, 157.53, 151.34, 149.57, 136.24, 132.83, 132.22, 131.59, 128.85, 120.06; HRMS (ESI+): *m/z* calcd for $C_{20}H_{16}N_5O_2S^+$ $[M+H]^+$: 390.10193; Found: 390.1020, Δ = 0.2563 ppm.

Compound 3si (SB2032): *N*-Benzyl-5'-(pyrimidin-2-yl)-[3,3'-bipyridin]-2-amine

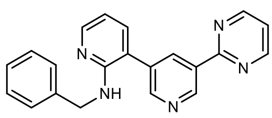

Following the general synthetic procedure 3, **1s** and **2i** were reacted for 16 h to provide **3si** in 83% yield. ^{1}H NMR (500 MHz, DMSO-d_6): δ 9.52 (d, *J* = 2.1 Hz, 1H), 8.99 (d, *J* = 4.9 Hz, 2H), 8.77 (d, *J* = 2.2 Hz, 1H), 8.73 (t, *J* = 2.2 Hz, 1H), 8.05 (dd, *J* = 5.0, 1.8 Hz, 1H), 7.55 (t, *J* = 4.9 Hz, 1H), 7.43 (dd, *J* = 7.2, 1.9 Hz, 1H), 7.33–7.25 (m, 4H), 7.20–7.15 (m, 1H), 6.71 (t, *J* = 6.0 Hz, 1H), 6.68 (dd, *J* = 7.2, 5.0 Hz, 1H), 4.53 (d, *J* = 5.9 Hz, 2H); ^{13}C NMR (126 MHz, DMSO-d_6): δ 161.73, 158.02, 155.45, 151.36, 147.67, 147.65, 141.15, 137.73, 135.38, 133.74, 132.70, 128.02, 126.99, 126.18, 120.68, 117.73, 112.34, 43.92; HRMS (ESI+): *m/z* calcd for $C_{21}H_{18}N_5^+$ [M+H]$^+$: 340.15568; Found: 340.1556, Δ = 0.2940 ppm.

Compound 3ti (SB2033): *N*-(4-Methoxybenzyl)-5'-(pyrimidin-2-yl)-[3,3'-bipyridin]-2-amine

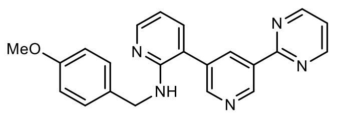

Following the general synthetic procedure 3, **1t** and **2i** were reacted for 16 h to provide **3ti** in 91% yield. ^{1}H NMR (500 MHz, DMSO-d_6): δ 9.51 (d, *J* = 2.1 Hz, 1H), 8.99 (d, *J* = 4.8 Hz, 2H), 8.81–8.66 (m, 2H), 8.06 (dd, *J* = 5.0, 1.9 Hz, 1H), 7.55 (t, *J* = 4.9 Hz, 1H), 7.42 (dd, *J* = 7.2, 1.9 Hz, 1H), 7.32–7.17 (m, 2H), 6.90–6.77 (m, 2H), 6.67 (dd, *J* = 7.2, 5.0 Hz, 1H), 6.62 (t, *J* = 6.0 Hz, 1H), 4.45 (d, *J* = 5.9 Hz, 2H), 3.70 (s, 3H); ^{13}C NMR (126 MHz, DMSO-d_6): δ 161.73, 158.01, 157.83, 155.44, 151.32, 147.68, 147.63, 137.69, 135.36, 133.76, 132.98, 132.70, 128.34, 120.68, 117.70, 113.44, 112.26, 54.98, 43.38; HRMS (ESI+): *m/z* calcd for $C_{22}H_{20}N_5O^+$ [M+H]$^+$: 370.16624; Found: 370.1662, Δ = 0 ppm.

Compound 3ui: *N*-(4-Nitrobenzyl)-5'-(pyrimidin-2-yl)-[3,3'-bipyridin]-2-amine

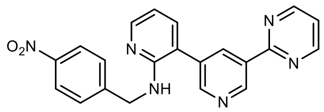

Following the general synthetic procedure 3, **1u** and **2i** were reacted for 16 h to provide **3ui** in 77% yield. ^{1}H NMR (500 MHz, DMSO-d_6): δ 9.54 (d, *J* = 2.0 Hz, 1H), 9.00 (d, *J* = 4.9 Hz, 2H), 8.82 (d, *J* = 2.2 Hz, 1H), 8.73 (t, *J* = 2.2 Hz, 1H), 8.22–8.13 (m, 2H), 8.03 (dd, *J* = 5.0, 1.8 Hz, 1H), 7.62–7.52 (m, 3H), 7.47 (dd, *J* = 7.2, 1.8 Hz, 1H), 6.92 (t, *J* = 5.9 Hz, 1H), 6.71 (dd, *J* = 7.2, 5.0 Hz, 1H), 4.62 (d, *J* = 5.9 Hz, 2H); ^{13}C NMR (126 MHz, DMSO-d_6): δ 161.72, 158.04, 155.11, 151.41, 149.81, 147.76, 147.65, 146.10, 137.92, 135.42, 133.58, 132.75, 127.95, 123.28, 120.72, 117.94, 112.78, 43.87; HRMS (ESI+): *m/z* calcd for $C_{21}H_{17}N_6O_2^+$ [M+H]$^+$: 385.14075; Found: 385.1408, Δ = 0 ppm.

Compound 3vi: *N*,5'-Di(pyrimidin-2-yl)-[3,3'-bipyridin]-2-amine

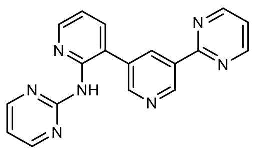

Following the general synthetic procedure 3, **1v** and **2i** were reacted for 16 h to provide **3vi** in 99% yield. ^{1}H NMR (500 MHz, DMSO-d_6): δ 9.68 (s, 1H), 9.33 (d, *J* = 2.0 Hz, 1H), 8.93 (d, *J* = 4.9 Hz, 2H), 8.74 (d, *J* = 2.2 Hz, 1H), 8.68 (t, *J* = 2.2 Hz, 1H), 8.49 (dd, *J* = 4.9, 1.8 Hz, 1H), 8.12 (d, *J* = 4.7 Hz, 2H), 7.95 (dd, *J* = 7.6, 1.9 Hz, 1H), 7.51

(t, J = 4.9 Hz, 1H), 7.39 (dd, J = 7.6, 4.8 Hz, 1H), 6.59 (t, J = 4.8 Hz, 1H); ^{13}C NMR (126 MHz, DMSO-d_6): δ 161.56, 159.97, 157.94, 157.59, 150.58, 150.40, 148.31, 147.15, 139.17, 134.68, 134.05, 131.97, 128.55, 121.22, 120.63, 112.48; HRMS (ESI+): *m/z* calcd for $C_{18}H_{14}N_7^+$ [M+H]$^+$: 328.13052; Found: 328.1306, Δ = 0.3048 ppm.

Compound 3wi: 2-(5-(Pyrimidin-2-yl)pyridin-3-yl)aniline

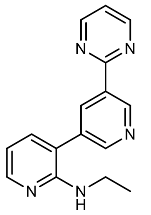

Following the general synthetic procedure 3, **1w** and **2i** were reacted for 16 h to provide **3wi** in 88% yield. ^{1}H NMR (400 MHz, DMSO-d_6): δ 9.50 (d, J = 2.03 Hz, 1H), 8.98 (d, J = 4.89 Hz, 2H), 8.72 (d, J = 2.16 Hz, 1H), 8.65 (t, J = 2.14 Hz, 1H), 8.10 (dd, J = 4.97, 1.82 Hz, 1H), 7.55 (t, J = 4.88 Hz, 1H), 7.38 (dd, J = 7.22, 1.88 Hz, 1H), 6.65 (dd, J = 7.25, 5.00 Hz, 1H), 6.06 (t, J = 5.63 Hz, 1H), 3.39 – 3.28 (m, 2H); ^{13}C NMR (101 MHz, DMSO-d_6): δ 161.73, 158.00, 155.68, 151.32, 147.74, 147.60, 137.61, 135.24, 133.84, 132.66, 120.65, 117.67, 111.85, 35.53, 14.85; HRMS (ESI+): *m/z* calcd for $C_{16}H_{16}N_5^+$ [M+H]$^+$: 278.14003; Found: 278.1401, Δ = 0.3595 ppm.

3.6. Synthetic procedures for synthetic applications (5a–5h & 6h)

Core remodeling of natural products (5a–5c)

To a mixture of 7*H*-[1,3]dioxolo[4,5-*j*]pyrrolo[3,2,1-*de*]phenanthridine-4-carbaldehyde (**4a**, 0.2 mmol), NH_4OAc (1.0 mmol. 5.0 equiv.), $Zn(OTf)_2$ (0.02 mmol, 10mol%) in EtOH (2.0 mL, 0.1 M) was added acetylene (**2c**, **2d**, or **2e**, 0.24 mmol, 1.2 equiv.). The reaction mixture was heated to 120 °C and stirred for the indicated time. The reaction progress was monitored by thin-layer chromatography and LC-MS. Upon completion, the reaction mixture was poured into brine, and the organic layer was extracted thrice with DCM. The collected organic layer was dried on anhydrous Na_2SO_4(s), filtered through cotton, and concentrated *in vacuo*. The crude mixture was purified by silica-gel flash column chromatography to afford the desired products (**5a**–**5c**).

Compound 5a: 4-(5-(Phenylsulfonyl)pyridin-3-yl)-[1,3]dioxolo[4,5-*j*]phenanthridine

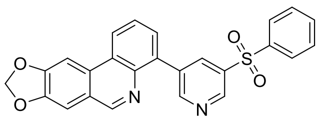

Following the synthetic procedure above, **4a** and **2c** were reacted for 16 h to provide **5a** in 43% yield. ^{1}H NMR (500 MHz, DMSO-d_6): δ 9.17 (d, J = 2.2 Hz, 1H), 9.14 (s, 1H), 9.13 (d, J = 2.1 Hz, 1H), 8.84 (dd, J = 8.6, 1.4 Hz, 1H), 8.65 (t, J = 2.2 Hz, 1H), 8.41 (s, 1H), 8.16–8.09 (m, 2H), 7.88 (dd, J = 7.2, 1.4 Hz, 1H), 7.79 (dd, J = 8.3, 7.2 Hz, 1H), 7.77–7.73 (m, 1H), 7.70–7.66 (m, 3H), 6.31 (s, 2H); ^{13}C NMR (126 MHz, DMSO-d_6): δ 155.08, 152.00, 151.80, 148.44, 146.10, 140.62, 140.58, 136.89, 136.81, 135.90, 134.72, 134.25, 129.98, 129.71, 129.35, 127.71, 126.60, 124.51, 124.40, 122.86, 105.28, 102.38, 100.59; HRMS (ESI+): *m/z* calcd for $C_{25}H_{17}N_2O_4S^+$ [M+H]$^+$: 441.09036; Found: 441.0907, Δ = 0.6801 ppm.

Compound 5b: 5-([1,3]Dioxolo[4,5-*j*]phenanthridin-4-yl)-*N*-(*tert*-butyl)pyridine-3-sulfonamide

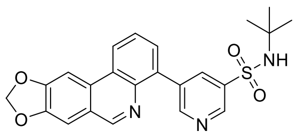

Following the synthetic procedure above, **4a** and **2d** were reacted for 16 h to provide **5b** in 47% yield. ^{1}H NMR (500 MHz, DMSO-d_6): δ 9.16 (s, 1H), 9.04 (d, J = 2.1 Hz, 1H), 8.99 (d, J = 2.3 Hz, 1H), 8.84 (dd, J = 8.4, 1.5 Hz, 1H), 8.53 (t, J = 2.2 Hz, 1H), 8.41 (s, 1H), 7.88–7.83 (m, 2H), 7.81 (t, J = 7.7 Hz, 1H), 7.70 (s, 1H), 6.31 (s, 2H), 1.21 (s, 9H); ^{13}C NMR (126 MHz, DMSO-d_6): δ 153.09, 151.86, 151.75, 148.42, 145.04, 140.69, 139.44, 136.10, 135.35, 135.28, 129.72, 129.07, 126.62, 124.55, 124.21, 122.85, 105.27, 102.35, 100.58, 53.78, 29.84; HRMS (ESI+): *m/z* calcd for $C_{23}H_{22}N_3O_4S^+$ [M+H]$^+$: 436.13256; Found: 436.1327, Δ = 0.2293 ppm.

Compound 5c: Ethyl (5-([1,3]dioxolo[4,5-*j*]phenanthridin-4-yl)pyridin-3-yl)(methyl)phosphinate

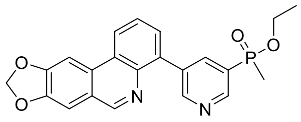

Following the synthetic procedure above, **4a** and **2e** were reacted for 16 h to provide **5c** in 48% yield. ^{1}H NMR (500 MHz, DMSO-d_6): δ 9.16 (s, 1H), 9.06 (t, J = 2.1 Hz, 1H), 8.91 (dd, J = 5.8, 1.9 Hz, 1H), 8.82 (dd, J = 8.4, 1.5 Hz, 1H), 8.41 (s, 1H), 8.38 (dt, J = 12.1, 2.1 Hz, 1H), 7.86 (dd, J = 7.2, 1.4 Hz, 1H), 7.82–7.77 (m, 1H), 7.68 (s, 1H), 6.31 (s, 2H), 4.13–3.82 (m, 2H), 1.80 (d, J = 14.8 Hz, 3H), 1.27 (t, J = 7.0 Hz, 3H); ^{13}C NMR (126 MHz, DMSO): δ 153.86, 151.82, 151.71, 149.66 (d, J = 12.0 Hz), 148.37, 140.75, 140.31 (d, J = 9.0 Hz), 135.98, 135.13 (d, J = 9.7 Hz), 129.71, 129.21, 127.21 (d, J = 122.5 Hz), 126.58, 124.51, 123.91, 122.81, 105.24, 102.34, 100.58, 60.38 (d, J = 6.0 Hz), 16.36 (d, J = 6.1 Hz), 15.22 (d, J = 101.5 Hz); HRMS (ESI+): *m/z* calcd for $C_{22}H_{20}N_2O_4P^+$ [M+H]$^+$: 407.11552; Found: 407.1157, Δ = 0.4913 ppm.

Synthesis of Ethyl 4-amino-3-(5-(3,4-dihydro-2*H*-pyrrol-5-yl)pyridin-3-yl)benzoate (5d) as drug-natural product conjugate[15]

To a mixture of ethyl 3-formyl-1*H*-indole-5-carboxylate (**4b**, 0.2 mmol), NH_4OAc (1.0 mmol. 5.0 equiv.), $Zn(OTf)_2$ (0.02 mmol, 10mol%) in EtOH (2.0 mL, 0.1 M) was added *tert*-butyl (4-oxohex-5-yn-1-yl)carbamate (**2o**, 0.24 mmol, 1.2 equiv.). The reaction mixture was heated to 120 °C and stirred for the indicated time. The reaction progress was monitored by thin-layer chromatography and LC-MS. Upon completion, the reaction mixture was poured into brine, and the organic layer was extracted thrice with DCM. The collected organic layer was dried on anhydrous Na_2SO_4(s), filtered through cotton, and concentrated *in vacuo*. The crude mixture was dissolved into 1 mL of DCM, and the solution was cooled to 0 °C. To the resulting solution was dropwisely added trifluoroacetic acid (TFA, 0.8 mL/mmol of SM). The reaction progress was monitored by thin-layer chromatography and LC-MS. After stirring at r.t. for 3 h, the reaction mixture was poured into a saturated aqueous sodium bicarbonate solution (sat. $NaHCO_{3(aq)}$), and the organic layer was extracted thrice with DCM. The collected organic layer was dried on anhydrous Na_2SO_4(s), filtered through cotton, and concentrated *in vacuo*. The crude mixture was purified by silica-gel flash column chromatography to afford the ethyl 4-amino-3-(5-(3,4-dihydro-2*H*-pyrrol-5-yl)pyridin-3-yl)benzoate (**5d**, 39%) as off-white solid.

Compound 5d: Ethyl 4-amino-3-(5-(3,4-dihydro-2*H*-pyrrol-5-yl)pyridin-3-yl)benzoate

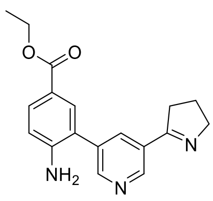

^{1}H NMR (400 MHz, $CDCl_3$): δ 8.97 (s, 1H), 8.81–8.66 (m, 1H), 8.26 (t, *J* = 2.1 Hz, 1H), 7.90–7.84 (m, 1H), 7.81 (d, *J* = 2.0 Hz, 1H), 6.76 (d, *J* = 8.5 Hz, 1H), 4.33 (dt, *J* = 14.3, 6.8 Hz, 4H), 4.08 (ddd, *J* = 7.5, 5.4, 2.2 Hz, 2H), 2.98 (ddt, *J* = 9.2, 4.2, 2.1 Hz, 2H), 2.14–2.02 (m, 2H), 1.36 (td, *J* = 7.0, 2.0 Hz, 3H); ^{13}C NMR (101 MHz, $CDCl_3$): δ 170.95, 166.49, 151.35, 148.31, 147.96, 135.30, 134.32, 132.57, 131.46, 130.36, 121.98, 120.38, 114.85, 61.73, 60.54, 34.99, 22.59, 14.46; HRMS (ESI+): *m/z* calcd for $C_{18}H_{20}N_3O_2^+$ $[M+H]^+$: 310.15501; Found: 310.1550, Δ = 0 ppm.

Synthesis of Nilotinib analogs (5e–5g)

To a mixture of 3-formyl-(aza)indole (**1a**, **4c**, **4d**, 0.2 mmol), NH_4OAc (1.0 mmol. 5.0 equiv.), $Zn(OTf)_2$ (0.02 mmol, 10mol%) in EtOH (2.0 mL, 0.1 M) was added 3-((4-ethynylpyrimidin-2-yl)amino)-4-methyl-*N*-(3-(4-methyl-1*H*-imidazol-1-yl)-5-(trifluoromethyl)phenyl)benzamide (**2p**, 0.4 mmol, 2.0 equiv.). The reaction mixture was heated to 120 °C and stirred for the indicated time. The reaction progress was monitored by thin-layer chromatography and LC-MS. Upon completion, the reaction mixture was poured into brine, and the organic layer was extracted thrice with DCM. The collected organic layer was dried on anhydrous Na_2SO_4(s), filtered through cotton, and concentrated *in vacuo*. The crude mixture was purified by high-performance liquid chromatography (HPLC) to afford the desired products (**5e**–**5g**).

Compound 5e: 3-((4-(2'-Amino-[3,3'-bipyridin]-5-yl)pyrimidin-2-yl)amino)-4-methyl-*N*-(3-(4-methyl-1*H*-imidazol-1-yl)-5-(trifluoromethyl)phenyl)benzamide

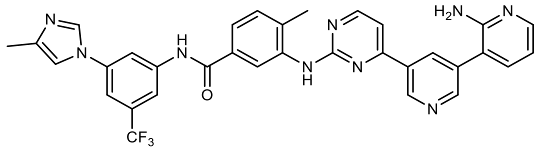

Following the synthetic procedure above, **1a** and **2p** were reacted for 16 h to provide **5e** in 45% yield. ^{1}H NMR (500 MHz, DMSO-d_6): δ 10.62 (s, 1H), 9.26 (d, *J* = 2.1 Hz, 1H), 9.19 (s, 1H), 8.71 (d, *J* = 2.1 Hz, 1H), 8.56 (d, *J* = 5.1 Hz, 1H), 8.50 (t, *J* = 2.2 Hz, 1H), 8.35–8.28 (m, 2H), 8.21 (d, *J* = 1.4 Hz, 1H), 8.17 (d, *J* = 1.7 Hz, 1H), 7.95 (dd, *J* = 4.9, 1.9 Hz, 1H), 7.76 (dd, *J* = 7.9, 1.9 Hz, 1H), 7.72 (d, *J* = 1.9 Hz, 1H), 7.56 (d, *J* = 5.2 Hz, 1H), 7.49 (d, *J* = 1.3 Hz, 1H), 7.45 (d, *J* = 8.0 Hz, 1H), 7.40 (dd, *J* = 7.4, 1.8 Hz, 1H), 6.60 (dd, *J* = 7.3, 4.9 Hz, 1H), 5.81 (s, 2H), 2.37 (s, 3H), 2.18 (s, 3H); ^{13}C NMR (126 MHz, DMSO-d_6): δ 165.64, 161.59, 161.03, 159.56, 156.86, 151.03, 147.94, 146.78, 141.41, 138.89, 138.20, 138.01, 137.91, 136.87, 134.97, 134.34, 134.13, 132.11, 131.77, 130.80 (q, *J* = 32.1 Hz), 130.42, 124.76, 124.35, 123.53, 122.59, 116.55, 114.93, 114.22, 112.88, 111.50, 108.18, 18.26, 13.56; HRMS (ESI+): *m/z* calcd for $C_{33}H_{27}F_3N_9O^+$ $[M+H]^+$: 622.22852; Found: 622.2284, Δ = 0.1607 ppm.

Compound 5f: 3-((4-(2'-Amino-5'-methyl-[3,3'-bipyridin]-5-yl)pyrimidin-2-yl)amino)-4-methyl-*N*-(3-(4-methyl-1*H*-imidazol-1-yl)-5-(trifluoromethyl)phenyl)benzamide

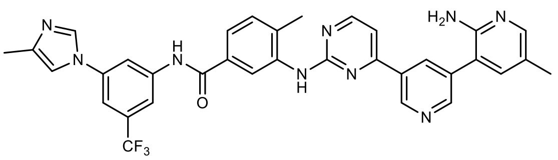

Following the synthetic procedure above, **4c** and **2p** were reacted for 16 h to provide **5f** in 72% yield. ^{1}H NMR (500 MHz, DMSO-d_6): δ 10.69–10.59 (m, 1H), 9.25 (d, *J* = 2.1 Hz, 1H), 9.19 (s, 1H), 8.71 (d, *J* = 2.1 Hz, 1H), 8.55 (d, *J* = 5.1 Hz, 1H), 8.49 (t, *J* = 2.2 Hz, 1H), 8.31–8.27 (m, 2H), 8.20 (d, *J* = 1.4 Hz, 1H), 8.16 (d, *J* = 1.7 Hz, 1H), 7.80 (d, *J* = 2.2 Hz, 1H), 7.77 (dd, *J* = 7.9, 1.9 Hz, 1H), 7.71 (d, *J* = 1.8 Hz, 1H), 7.56 (d, *J* = 5.2 Hz, 1H), 7.48 (s, 1H), 7.45 (d, *J* = 8.0 Hz, 1H), 7.26 (d, *J* = 2.4 Hz, 1H), 5.56 (s, 2H), 2.36 (s, 3H), 2.18 (s, 3H), 2.12 (s, 3H); ^{13}C NMR (126 MHz, DMSO-d_6): δ 165.64, 161.62, 161.04, 159.54, 154.85, 151.00, 147.53, 146.69, 141.44, 138.88, 138.82, 138.20, 137.88, 136.94, 134.95, 134.28, 134.19, 132.07, 131.80, 130.77 (d, *J* = 32.2 Hz), 130.42, 124.76, 124.41, 123.58, 122.59, 121.39, 116.27, 114.91, 114.21, 111.46, 108.17, 18.26, 16.74, 13.56; HRMS (ESI+): *m/z* calcd for $C_{34}H_{29}F_3N_9O^+$ [M+H]$^+$: 636.24417; Found: 636.2443, Δ = 0.1572 ppm.

Compound 5g: 3-((4-(5-(2-Hydroxyphenyl)pyridin-3-yl)pyrimidin-2-yl)amino)-4-methyl-*N*-(3-(4-methyl-1*H*-imidazol-1-yl)-5-(trifluoromethyl)phenyl)benzamide

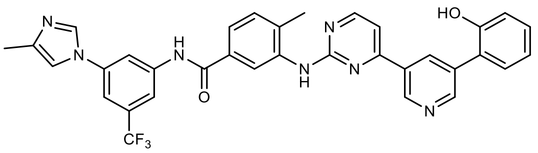

Following the synthetic procedure above, **4d** and **2p** were reacted for 16 h to provide **5g** in 32% yield. ^{1}H NMR (500 MHz, DMSO-d_6): δ 10.58 (s, 1H), 9.80 (s, 1H), 9.23 – 9.16 (m, 2H), 8.86 (d, *J* = 2.1 Hz, 1H), 8.58 – 8.52 (m, 2H), 8.32 (d, *J* = 1.9 Hz, 1H), 8.27 (d, *J* = 2.1 Hz, 1H), 8.19 (d, *J* = 1.4 Hz, 1H), 8.15 (s, 1H), 7.76 (dd, *J* = 7.9, 1.9 Hz, 1H), 7.71 (s, 1H), 7.55 (d, *J* = 5.2 Hz, 1H), 7.47 (s, 1H), 7.45 (d, *J* = 8.0 Hz, 1H), 7.34 (dd, *J* = 7.7, 1.7 Hz, 1H), 7.20 – 7.14 (m, 1H), 6.95 (d, *J* = 8.1 Hz, 1H), 6.84 (t, *J* = 7.5 Hz, 1H), 2.37 (s, 3H), 2.17 (s, 2H); ^{13}C NMR (126 MHz, DMSO-d_6): δ 165.63, 161.79, 161.05, 159.54, 154.71, 151.49, 145.91, 141.43, 138.88, 138.21, 137.88, 136.88, 134.95, 134.38, 134.21, 131.75, 131.63, 130.78 (q, *J* = 32.2 Hz), 130.43, 130.16, 129.52, 124.76, 124.30, 123.74, 123.57, 122.59, 119.10, 116.27, 114.93, 114.21, 111.48, 108.07, 18.26, 13.55; HRMS (ESI+): *m/z* calcd for $C_{34}H_{27}F_3N_7O_2^+$ [M+H]$^+$: 622.21729; Found: 622.2175, Δ = 0.3214 ppm.

Synthesis of 5-(2-Amino-6-fluoro-[1,2,4]triazolo[1,5-*a*]pyridin-8-yl)-*N*-(*tert*-butyl)pyridine-3-sulfonamide (6h)

Compound 5h: 2'-Amino-*N*-(*tert*-butyl)-5'-fluoro-[3,3'-bipyridine]-5-sulfonamide

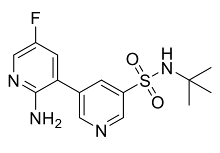

To a mixture of 5-fluoro-1*H*-pyrrolo[2,3-*b*]pyridine-3-carbaldehyde (**4e**, 0.2 mmol), NH_4OAc (1.0 mmol. 5.0 equiv.), $Zn(OTf)_2$ (0.02 mmol, 10mol%) in EtOH (2.0 mL, 0.1 M) was added *N*-(*tert*-butyl)ethynesulfonamide (**2d**, 0.24 mmol, 1.2 equiv.). The reaction mixture was heated to 120 °C, and stirred for indicated time. The reaction progress was

monitored by thin-layer chromatography and LC-MS. Upon completion, the reaction mixture was poured into brine, and the organic layer was extracted with DCM thrice. The collected organic layer was dried on anhydrous Na_2SO_4(s), filtered through cotton, and concentrated *in vacuo*. The crude mixture was purified by silica-gel flash column chromatography to afford the 2'-amino-*N*-(*tert*-butyl)-5'-fluoro-[3,3'-bipyridine]-5-sulfonamide (**5h**, 53%) as white solid.

^{1}H NMR (500 MHz, DMSO-d_6): δ 8.94 (d, J = 2.2 Hz, 1H), 8.85 (d, J = 2.1 Hz, 1H), 8.25 (t, J = 2.2 Hz, 1H), 8.04 (d, J = 3.0 Hz, 1H), 7.78 (s, 1H), 7.49 (dd, J = 8.8, 2.9 Hz, 1H), 5.83 (s, 2H), 1.15 (s, 9H); ^{13}C NMR (126 MHz, DMSO): δ 153.94, 152.15, 145.76, 140.33, 134.82, 133.89 (d, J = 187.9 Hz), 133.79, 125.90, 125.74, 116.41, 53.86, 29.84; HRMS (ESI+): *m/z* calcd for $C_{14}H_{18}FN_4O_2S^+$ $[M+H]^+$: 325.11291; Found: 325.1128, Δ = 0.3076 ppm.

Compound 6h: 5-(2-Amino-6-fluoro-[1,2,4]triazolo[1,5-*a*]pyridin-8-yl)-*N*-(*tert*-butyl)pyridine-3-sulfonamide

The cyclization of [1,2,4]triazolo[1,5-*a*]pyridine ring was done following the modified procedure from the literature.[16] 2'-Amino-*N*-(*tert*-butyl)-5'-fluoro-[3,3'-bipyridine]-5-sulfonamide (**5h**, 42 mg, 0.13 mmol) was suspended to 1 mL of ethyl acetate and cooled to 0 °C. Ethoxycarbonyl isothiocyanate (46 μL, 0.20 mmol, 1.5 equiv.) was added dropwise over 15 min. The reaction mixture was then warmed to 40 °C and stirred for 6 h. A further aliquot of ethoxycarbonyl isothiocyanate (46 μL, 0.20 mmol, 1.5 equiv.) was added, and the reaction mixture was stirred at 40 °C for 18 h. Then, the reaction mixture was cooled to 0 °C, and hexane was added. The resulting solid was filtered, washed with further hexane, and air-dried. To a suspension of hydroxylamine hydrochloride (45 mg, 0.65 mmol, 5.0 equiv.) in EtOH/MeOH (1:1, 1.3 mL) was added *N*,*N*-diisopropylethylamine (67 μL, 0.39 mmol, 3.0 equiv.), and the mixture was stirred at r.t. for 1 h. The air-dried intermediate was added to this solution. The reaction mixture was then slowly heated to reflux. After 24 h, the mixture was allowed to cool to r.t. The formed solid was filtered and purified through HPLC to provide 5-(2-amino-6-fluoro-[1,2,4]triazolo[1,5-*a*]pyridin-8-yl)-*N*-(*tert*-butyl)pyridine-3-sulfonamide (**6h**, 33%) as white solid.

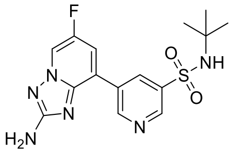

^{1}H NMR (500 MHz, DMSO-d_6): δ 9.48 (d, J = 2.1 Hz, 1H), 9.04 (dd, J = 3.8, 2.4 Hz, 1H), 9.02 (d, J = 2.2 Hz, 1H), 8.97 (t, J = 2.2 Hz, 1H), 8.10 (dd, J = 9.8, 2.4 Hz, 1H), 7.86 (s, 1H), 6.27 (s, 2H), 1.15 (s, 9H); ^{13}C NMR (126 MHz, DMSO): δ 166.95, 151.66, 151.54 (d, J = 230.8 Hz), 146.74, 146.54, 140.32, 133.32, 130.56, 119.13 (d, J = 8.8 Hz), 117.80 (d, J = 25.0 Hz), 116.33 (d, J = 40.7 Hz), 53.91, 29.82; HRMS (ESI+): *m/z* calcd for $C_{15}H_{18}FN_6O_2S^+$ $[M+H]^+$: 365.11905; Found: 365.1189, Δ = 0.5478 ppm.

Structure-activity relationship (SAR) library (SB2034–SB2040)

Compound SB2034: 6-Bromo-5'-(pyrimidin-2-yl)-[3,3'-bipyridin]-2-amine

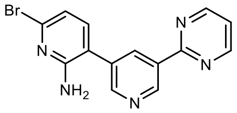

Following the general synthetic procedure 3, **4f** and **2i** were reacted for 16 h to provide **SB2034** in 39% yield after HPLC purification. ^{1}H NMR (500 MHz, DMSO-d_6): δ 9.49 (d, J = 2.1 Hz, 1H), 8.98 (d, J = 4.8 Hz, 2H), 8.72 (d, J = 2.2 Hz, 1H), 8.69 (t, J = 2.1 Hz, 1H), 7.55 (t, J = 4.9 Hz, 1H), 7.38 (d, J = 7.6 Hz, 1H), 6.85 (d, J = 7.6 Hz, 1H), 6.41 (s, 2H); ^{13}C NMR (126 MHz, DMSO-d_6): δ 161.67, 157.98, 157.35, 151.02, 147.72, 140.84, 139.26, 134.90, 132.86, 132.58, 120.68, 115.64, 115.30; HRMS (ESI+): *m/z* calcd for $C_{14}H_{11}BrN_5^+$ [M+H]$^+$: 328.01924; Found: 328.0193, Δ = 0.3049 ppm.

Compound SB2035: *N*-Benzyl-5-bromo-2-(5-(pyrimidin-2-yl)pyridin-3-yl)aniline

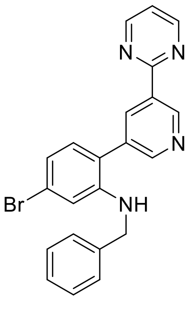

Following the general synthetic procedure 3, **4g** and **2i** were reacted for 16 h to provide **SB2035** in 53% yield after HPLC purification. ^{1}H NMR (500 MHz, DMSO-d_6): δ 9.52 (d, J = 2.1 Hz, 1H), 9.00 (d, J = 4.9 Hz, 2H), 8.75 (d, J = 2.2 Hz, 1H), 8.71 (t, J = 2.2 Hz, 1H), 7.55 (t, J = 4.9 Hz, 1H), 7.38–7.30 (m, 4H), 7.23 (td, J = 6.6, 3.2 Hz, 1H), 7.02 (d, J = 7.9 Hz, 1H), 6.82 (dd, J = 8.0, 1.9 Hz, 1H), 6.67 (d, J = 1.9 Hz, 1H), 6.11 (t, J = 6.0 Hz, 1H), 4.30 (d, J = 6.0 Hz, 2H); ^{13}C NMR (126 MHz, DMSO-d_6): δ 161.75, 158.01, 151.43, 147.59, 146.89, 139.52, 135.53, 134.16, 132.68, 132.02, 128.40, 126.80, 126.70, 122.71, 122.26, 120.66, 118.71, 113.25, 46.04; HRMS (ESI+): *m/z* calcd for $C_{22}H_{18}BrN_4^+$ [M+H]$^+$: Exact Mass: 417.07094; Found: 417.0715, Δ = 1.4386 ppm.

Compound SB2036: 5-Bromo-*N*-(4-methoxybenzyl)-2-(5-(pyrimidin-2-yl)pyridin-3-yl)aniline

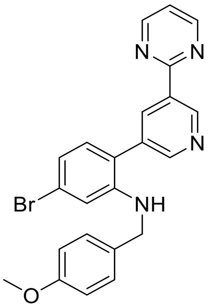

Following the general synthetic procedure 3, **4h** and **2i** were reacted for 16 h to provide **SB2036** in 61% yield after HPLC purification. ^{1}H NMR (500 MHz, DMSO-d_6): δ 9.52 (d, J = 2.0 Hz, 1H), 9.00 (d, J = 4.9 Hz, 2H), 8.74 (d, J = 2.2 Hz, 1H), 8.69 (t, J = 2.2 Hz, 1H), 7.56 (q, J = 5.6, 5.0 Hz, 1H), 7.27 (d, J = 8.3 Hz, 2H), 7.01 (d, J = 8.0 Hz, 1H), 6.93 – 6.86 (m, 2H), 6.82 (dd, J = 8.0, 1.9 Hz, 1H), 6.69 (d, J = 1.9 Hz, 1H), 6.06 (t, J = 6.0 Hz, 1H), 4.22 (d, J = 5.9 Hz, 2H), 3.72 (s, 3H); ^{13}C NMR (126 MHz, DMSO-d_6): δ 161.76, 158.10, 158.04, 151.43, 147.58, 146.89, 135.53, 134.20, 132.69, 132.01, 131.21, 128.05, 122.72, 122.24, 120.69, 118.64, 113.82, 113.33, 54.99, 45.45; HRMS (ESI+): *m/z* calcd for $C_{23}H_{20}BrN_4O^+$ [M+H]$^+$: 447.08150; Found: 447.0816, Δ = 0.2237 ppm.

Compound SB2037: 5-Fluoro-2-(5-(pyrimidin-2-yl)pyridin-3-yl)aniline

Following the general synthetic procedure 3, **4i** and **2i** were reacted for 16 h to provide **SB2037** in 43% yield after

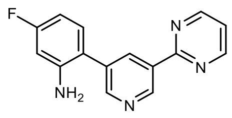

HPLC purification.^{1}H NMR (500 MHz, DMSO-d_6): δ 9.46 (d, *J* = 2.1 Hz, 1H), 8.98 (d, *J* = 4.9 Hz, 2H), 8.69 (dd, *J* = 11.9, 2.2 Hz, 2H), 7.54 (t, *J* = 4.8 Hz, 1H), 7.10 (dd, *J* = 8.4, 6.7 Hz, 1H), 6.58 (dd, *J* = 11.7, 2.6 Hz, 1H), 6.46 (td, *J* = 8.4, 2.6 Hz, 1H), 5.35 (s, 2H); ^{13}C NMR (126 MHz, DMSO): δ 163.11 (d, *J* = 241.4 Hz), 161.83, 157.97, 151.28, 147.95 (d, *J* = 11.8 Hz), 147.09, 135.15, 134.53, 132.49, 131.93 (d, *J* = 10.5 Hz), 120.61, 118.18 (d, *J* = 2.4 Hz), 103.13 (d, *J* = 21.8 Hz), 101.23 (d, *J* = 24.6 Hz); HRMS (ESI+) *m/z* calcd for $C_{15}H_{12}FN_4^+$ $[M+H]^+$: 267.10405; found: 267.1038, Δ = 1.1232 ppm.

Compound SB2038: 5-Chloro-2-(5-(pyrimidin-2-yl)pyridin-3-yl)aniline

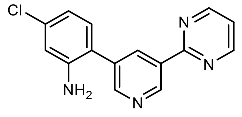

Following the general synthetic procedure 3, **4j** and **2i** were reacted for 16 h to provide **SB2038** in 47% yield after HPLC purification.^{1}H NMR (500 MHz, DMSO-d_6): δ 9.47 (d, *J* = 2.0 Hz, 1H), 8.98 (d, *J* = 4.9 Hz, 2H), 8.72 (d, *J* = 2.2 Hz, 1H), 8.69 (t, *J* = 2.1 Hz, 1H), 7.55 (t, *J* = 4.9 Hz, 1H), 7.09 (d, *J* = 8.1 Hz, 1H), 6.85 (d, *J* = 2.1 Hz, 1H), 6.68 (dd, *J* = 8.1, 2.1 Hz, 1H), 5.36 (s, 2H); ^{13}C NMR (126 MHz, DMSO-d_6): δ 161.78, 157.98, 151.15, 147.46, 147.28, 135.02, 134.31, 133.52, 132.51, 131.84, 120.64, 120.51, 116.15, 114.41; HRMS (ESI+): *m/z* calcd for $C_{15}H_{12}ClN_4^+$ $[M+H]^+$: 283.07450; Found: 283.0743, Δ = 0.7065 ppm.

Compound SB2039: 5-Methyl-2-(5-(pyrimidin-2-yl)pyridin-3-yl)aniline

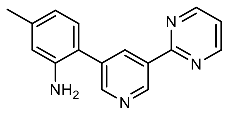

Following the general synthetic procedure 3, **4k** and **2i** were reacted for 16 h to provide **SB2039** in 51% yield after HPLC purification. ^{1}H NMR (500 MHz, DMSO-d_6): δ 9.43 (d, *J* = 2.0 Hz, 1H), 8.97 (d, *J* = 4.8 Hz, 2H), 8.71 (dd, *J* = 5.7, 2.1 Hz, 2H), 7.54 (t, *J* = 4.9 Hz, 1H), 6.98 (d, *J* = 7.6 Hz, 1H), 6.63 (d, *J* = 1.6 Hz, 1H), 6.52 (dd, *J* = 7.9, 1.7 Hz, 1H), 4.94 (s, 2H), 2.22 (s, 3H); ^{13}C NMR (126 MHz, DMSO-d_6): δ 161.92, 157.96, 151.20, 146.76, 145.56, 138.36, 135.44, 134.90, 132.42, 130.18, 120.57, 119.13, 117.96, 116.08, 20.98; HRMS (ESI+): *m/z* calcd for $C_{16}H_{15}N_4^+$ $[M+H]^+$: 263.12913; Found: 263.1288, Δ = 1.1401 ppm.

Compound SB2040: Methyl 3-amino-4-(5-(pyrimidin-2-yl)pyridin-3-yl)benzoate

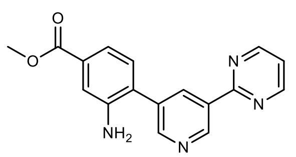

Following the general synthetic procedure 3, **4l** and **2i** were reacted for 16 h to provide **SB2040** in 63% yield after HPLC purification. ^{1}H NMR (500 MHz, DMSO-d_6): δ 9.50 (d, *J* = 2.0 Hz, 1H), 8.98 (d, *J* = 4.9 Hz, 2H), 8.77 (d, *J* = 2.2 Hz, 1H), 8.74 (t, *J* = 2.1 Hz, 1H), 7.55 (t, *J* = 4.9 Hz, 1H), 7.47 (d, *J* = 1.6 Hz, 1H), 7.31 – 7.18 (m, 2H), 5.37 (s, 2H), 3.85 (s, 3H); ^{13}C NMR (126 MHz, DMSO-d_6): δ 166.44, 161.73, 157.98, 150.99, 147.54, 146.23, 134.92, 134.47, 132.52, 130.67, 130.18, 125.99, 120.66, 117.18, 116.00, 52.01; HRMS (ESI+): *m/z* calcd for $C_{17}H_{15}N_4O_2^+$ $[M+H]^+$: 307.11896; Found: 307.1188, Δ = 0.6512 ppm.

4. Supplementary References

[1] Varun, B. V., Vaithegi, K., Yi, S. & Park, S. B. Nature-inspired remodeling of (aza)indoles to *meta*-aminoaryl nicotinates for late-stage conjugation of vitamin B_3 to (hetero)arylamines, *Nat. Commun.* **11**, 6308 (2020).

[2] Vaithegi, K., Yi, S., Lee, J. H., Varun, B. V. & Park, S. B. Synthesis of substituted pyridines with diverse functional groups via the remodeling of (Aza)indole/Benzofuran skeletons, *Commun. Chem.* **6**, 112 (2023).

[3] Hara, T., Durell, S. R., Myers, M. C. & Appella, D. H. Probing the structural requirements of peptoids that inhibit HDM2-p53 interactions. *J. Am. Chem. Soc.* **128**, 1995–2004 (2006).

[4] Li, L.-T., Huang, J., Li, H.-Y., Wen, L.-J., Wang, P. & Wang, B. *n*Bu_4NI-catalyzed C3-formylation of indoles with N-methylaniline. *Chem. Commun.* **48**, 5187–5189 (2012).

[5] Herck. N. V., Maes, D., Unal, K., Guerre, M., Winne, J. M. & Du Prez, F. E. Covalent adaptable networks with tunable exchange rates based on reversible thiol-yne cross-linking. *Angew. Chem. Int. Ed.* **59**, 3609–3617 (2020).

[6] Karaj, E. Sindi, S. H., Kuganesan, N. Perera, L., Taylor, W. & Tillekeratne, L. M. V. Tunable cysteine-targeting electrophilic heteroaromatic warheads induce ferroptosis. *J. Med. Chem.* **65**, 11788–11817 (2022).

[7] Majumder, B. & Pandey, G. Synthesis of 2-azabicyclo[*m*,*n*,0]-alkaloids and their application towards the synthesis of strychnos and stemona classes of alkaloids. *Eur. J. Org. Chem.* **2020**, 3883–3888 (2020).

[8] Lee, Y. S., Chung, K. H. & Kim, Y. H. New cyclization of *N*-hydroxyiminoyl chlorides with *N*-alkyl ethynesulfonamides: synthesis of 4-alkyl-3-aryl-4,5-dihydro-1,5,2,4-oxathiadiazepine-5,5-diones. *Heteroat. Chem.* **10**, 461–464 (1999).

[9] Aguiar, A. M. & Chattha, M. S. Covenient synthesis of 1-alkynylphosphonates. *J. Org. Chem.* **36**, 2719–2720 (1971).

[10] Hay, D. A. *et. al.* Design and synthesis of potent and selective inhibitors of BRD7 and BRD9 bromodomains. *Med. Chem. Commun.* **6**, 1381–1386 (2015).

[11] Yang, Z., Li, J., Yang, T. & Zhou, C. CuI assisted desulfurative sonogashira reaction of mercapto *N*-heterocyclic derivatives with alkynes. *RSC Adv.* **6**, 65775–65778 (2016).

[12] Xaio, X.-S., Zou, C., Guan, X., Yang, C., Lu, W. & Che, C.-M. Homoleptic gold(I) *N*-heterocyclic allenylidene complexes: excited-state properties and lyotropic chromonics. *Chem. Commun.* **52**, 4983–4986 (2016).

[13] Vu, H.-D., Renault, J., Roisnel, T., Gouault, N. & Uriac, P. Methanesulfonic acid-mediated cyclization and Meyer-Schuster rearrangement of γ-amino ynones. Access to enaiopure pyrrolidine exocyclic vinylogous amides. *Eur. J. Org. Chem.* **2014**, 4506‒4514 (2014).

[14] Ueda, S., Su, M. & Buchwald, S. L. Completely N^1-selective palladium-catalyzed arylation of unsymmetric imidazoles: application to the synthesis of Nilotinib. *J. Am. Chem. Soc.* **134**, 700–706 (2012).

[15] Guo, C., Sun, D.-W., Yang, S., Mao, S.-J., Xu, X.-H., Zhu, S.-F. & Zhou, Q.-L. Iridium-catalyzed asymmetric hydrogenation of 2-pyridyl cyclic imines: a highly enantioselective approach to nicotine derivatives. *J. Am. Chem. Soc.* **137**, 90–93 (2015).

[16] Bergamini, G. *et. al.* A selective inhibitor reveals PI3Kγ dependence of T_H17 cell differentiation. *Nat. Chem. Biol.* **8**, 576–582 (2012).
