# Supplementary material for: Unveiled reactivity of masked diformylmethane with enamines forming resonance-assisted hydrogen bonding leads to di-meta-substituted pyridines
Source: Commun Chem. 2024 Jun 28;7:146. doi: 10.1038/s42004-024-01228-w (PMC11213866; doi:10.1038/s42004-024-01228-w)
Supplement: Supplementary file 3 — Description of Additional Supplementary Files [file 42004_2024_1228_MOESM3_ESM.pdf]

Description of Additional Supplementary Files

File name: Supplementary Data 1

Description: 1H, 13C NMR spectra.

File name: Supplementary Data 2

Description: Bio Source Data.
