# Supplementary material for: Unveiled reactivity of masked diformylmethane with enamines forming resonance-assisted hydrogen bonding leads to di-meta-substituted pyridines
Source: Commun Chem. 2024 Jun 28;7:146. doi: 10.1038/s42004-024-01228-w (PMC11213866; doi:10.1038/s42004-024-01228-w)
Supplement: Supplementary file 4 — Supplementary Data 1 [file 42004_2024_1228_MOESM4_ESM.docx]

**^1^H & ^13^C NMR Spectra of All New Compounds**

**1. Starting materials**

**2. Acetylenes**

**3. Alkyne substrate scope**

**4. (Aza)indole substrate scope with 2e (3be–3we)**

**5. (Aza)indole substrate scope with 2i (3bi–3wi)**

**6. Synthetic applications (5a–5h, 6h)**

**4.7. Structure-activity relationship study (SB2034–SB2040)**
