# Supplementary material for: Unveiled reactivity of masked diformylmethane with enamines forming resonance-assisted hydrogen bonding leads to di-meta-substituted pyridines
Source: Commun Chem. 2024 Jun 28;7:146. doi: 10.1038/s42004-024-01228-w (PMC11213866; doi:10.1038/s42004-024-01228-w)
Supplement: Supplementary file 6 — Reporting Summary [file 42004_2024_1228_MOESM6_ESM.pdf]

nature portfolio

Corresponding author(s): Seung Bum Park

Last updated by author(s): Jun 9, 2024

Reporting Summary

Nature Portfolio wishes to improve the reproducibility of the work that we publish. This form provides structure for consistency and transparency in reporting. For further information on Nature Portfolio policies, see our Editorial Policies and the Editorial Policy Checklist.

Statistics

For all statistical analyses, confirm that the following items are present in the figure legend, table legend, main text, or Methods section.

n/a	Confirmed	
☐	☒	The exact sample size (*n*) for each experimental group/condition, given as a discrete number and unit of measurement
☐	☒	A statement on whether measurements were taken from distinct samples or whether the same sample was measured repeatedly
☐	☒	The statistical test(s) used AND whether they are one- or two-sided<br>*Only common tests should be described solely by name; describe more complex techniques in the Methods section.*
☒	☐	A description of all covariates tested
☐	☒	A description of any assumptions or corrections, such as tests of normality and adjustment for multiple comparisons
☐	☒	A full description of the statistical parameters including central tendency (e.g. means) or other basic estimates (e.g. regression coefficient) AND variation (e.g. standard deviation) or associated estimates of uncertainty (e.g. confidence intervals)
☐	☒	For null hypothesis testing, the test statistic (e.g. *F*, *t*, *r*) with confidence intervals, effect sizes, degrees of freedom and *P* value noted<br>*Give P values as exact values whenever suitable.*
☒	☐	For Bayesian analysis, information on the choice of priors and Markov chain Monte Carlo settings
☒	☐	For hierarchical and complex designs, identification of the appropriate level for tests and full reporting of outcomes
☐	☒	Estimates of effect sizes (e.g. Cohen's *d*, Pearson's *r*), indicating how they were calculated

Our web collection on statistics for biologists contains articles on many of the points above.

Software and code

Policy information about availability of computer code

Data collection	Absorbance measurements in 96-well plate for Griess, viability, ELISA and BCA assay were performed with BioTek Synergy HT microplate reader [Winooski, VT]. ChemiDocTM MP imaging system from Bio-Rad was used to analyze chemiluminescent signals for immunoblot assay. Chemiluminescence signals were quantified by ImageLab 6.0 program provided by Bio-Rad [Hercules, CA]. RNA quantification was done by Nanodrop from Thermofisher [Waltham, MA]. Quantitative real-time PCR was performed with StepOne Plus Real-Time PCR system from Applied Biosystems [Foster city, CA]. Delta Vision Elite imaging system from Cytiva [Marlborough, MA] was used to analyze immunofluorescence signals. For flow cytometry experiments, cells were analyzed using a CytoFLEX LX from Beckman Coulter [Brea, CA]. Data acquisition was performed with CytoExpert 2.4 software [Brea, CA].
Data analysis	Provided graphs were analyzed with GraphPad Prism 8 [La Jolla, CA]. Flow cytometry data was analyzed using CytoExpert 2.4 software or FlowJo V10 software from Tree Star [Ashland, OR]. Immunofluorescence images were analyzed and merged with SoftWorks deconvolution software from Cytiva. No custom code was used in this paper.

For manuscripts utilizing custom algorithms or software that are central to the research but not yet described in published literature, software must be made available to editors and reviewers. We strongly encourage code deposition in a community repository (e.g. GitHub). See the Nature Portfolio guidelines for submitting code & software for further information.

Data

Policy information about availability of data

All manuscripts must include a data availability statement. This statement should provide the following information, where applicable:

- Accession codes, unique identifiers, or web links for publicly available datasets
- A description of any restrictions on data availability
- For clinical datasets or third party data, please ensure that the statement adheres to our policy

All data generated or analyzed during this study are included in the article and its associated files. Source data are provided with this paper. All other data are available from the corresponding authors on request.

Human research participants

Policy information about studies involving human research participants and Sex and Gender in Research.

Reporting on sex and gender	N/A
Population characteristics	N/A
Recruitment	N/A
Ethics oversight	N/A

Note that full information on the approval of the study protocol must also be provided in the manuscript.

Field-specific reporting

Please select the one below that is the best fit for your research. If you are not sure, read the appropriate sections before making your selection.

☒ Life sciences ☐ Behavioural & social sciences ☐ Ecological, evolutionary & environmental sciences

For a reference copy of the document with all sections, see nature.com/documents/nr-reporting-summary-flat.pdf

Life sciences study design

All studies must disclose on these points even when the disclosure is negative.

Sample size	No any sample size calculation was performed. Sample size was chosen such that the statistical significance is confidently established.
Data exclusions	No any data were excluded.
Replication	All attempts at replication were successful.
Randomization	All samples and cells were randomized in this study.
Blinding	Investigators were blinded to group allocation during data collection and analysis.

Reporting for specific materials, systems and methods

We require information from authors about some types of materials, experimental systems and methods used in many studies. Here, indicate whether each material, system or method listed is relevant to your study. If you are not sure if a list item applies to your research, read the appropriate section before selecting a response.

Materials & experimental systems

n/a	Involved in the study	
☐	☒	Antibodies
☐	☒	Eukaryotic cell lines
☒	☐	Palaeontology and archaeology
☒	☐	Animals and other organisms
☒	☐	Clinical data
☒	☐	Dual use research of concern

Methods

n/a	Involved in the study	
☒	☐	ChIP-seq
☐	☒	Flow cytometry
☒	☐	MRI-based neuroimaging

Antibodies

Antibodies used

The following antibodies were purchased as primary antibodies for immunoblot studies: rabbit anti-β-tubulin polyclonal antibody (2146, Cell Signaling Technology); rabbit anti-GAPDH monoclonal antibody (2118, Cell Signaling Technology); rabbit anti-SAPK/JNK polyclonal antibody (Cell Signaling Technology, 9252); rabbit anti-phospho-SAPK/JNK (Thr183/Tyr185) polyclonal antibody (Cell Signaling Technology, 9251); rabbit anti-p44/42 MAPK (Erk1/2) monoclonal antibody (Cell Signaling Technology, 4695); rabbit anti-phospho-P44/42 MAPK (Erk1/2) (Thr202/Tyr204) polyclonal antibody (Cell Signaling Technology, 9101); rabbit anti-p38 MAPK polyclonal antibody (Cell Signaling Technology, 9212); rabbit anti-phospho-p38 MAPK (Thr180/Tyr182) polyclonal antibody (Cell Signaling Technology, 9211); rabbit anti-IL-1β monoclonal antibody (Cell Signaling Technology, 31202); rabbit anti-Cleaved-IL-1β monoclonal antibody (Cell Signaling Technology, 63124); rabbit anti-NF-κB p65 polyclonal antibody (Abcam, ab16502); rabbit anti-c-Jun polyclonal antibody (Abcam, ab31419); rabbit anti-Lamin B1 polyclonal antibody (Abcam, ab16048).
The following antibodies were purchased for immunofluorescence studies: mouse anti-p65 antibody (sc-8008, Santa Cruz); goat anti-mouse IgG (FITC) (ab6785, Abcam).

Validation

Each primary antibody was validated according to the manufacturer's website and by immunoblotting and immunofluorescence staining in this study. Specifically, antibodies from Abcam, Cell Signaling Technology, and Santa Cruz were validated by functional immunoblotting and immunofluorescence imaging.

Eukaryotic cell lines

Policy information about cell lines and Sex and Gender in Research

Cell line source(s)

RAW264.7 and J774A.1 mouse macrophage cell lines were obtained from American Type Culture Collection.

Authentication

All cells were used without modification once received from the supplier, and therefore were not authenticated.

Mycoplasma contamination

All cells were tested negative for mycoplasma contamination.

Commonly misidentified lines
(See ICLAC register)

No any commonly misidentified cell lines were used in the study.

Flow Cytometry

Plots

Confirm that:

☒ The axis labels state the marker and fluorochrome used (e.g. CD4-FITC).

☒ The axis scales are clearly visible. Include numbers along axes only for bottom left plot of group (a 'group' is an analysis of identical markers).

☒ All plots are contour plots with outliers or pseudocolor plots.

☒ A numerical value for number of cells or percentage (with statistics) is provided.

Methodology

Sample preparation

RAW264.7 cells were treated with SB2037 and LPS for 24 h at 37 °C in a 5% CO2 humidified incubator. For reactive oxygen species measurement assay, 10 µM of dichlorodihydrofluorescein diacetate (DCFH-DA, Sigma-Aldrich, 35845) was added to the cells for an additional 0.5 h. The resulting cells were subjected to flow cytometry analysis using CytoFLEX LX II [Beckman Coulter], installed at Danaher-SNU Discovery Center at Seoul National University. Data were analyzed to measure the fluorescence intensity of ROS using FlowJo V10 software. Mean values of FITC fluorescence intensity per cell were used for the analysis.

Instrument

CytoFLEX LX from Beckman Coulter

Software

CytoExpert 2.4 software was used for data acquisition, and Flowjo version 10 or CytoExpert 2.4 software was used for data analysis.

Cell population abundance	N/A
Gating strategy	To isolate single cells from non-singlet cells, gating of FSC-A vs FSC-H was done, followed by FSC-A vs SSC-A. Compared to WT RAW264.7 cells, FITC-positive cells were analyzed for ROS assessment via DCFH-DA.

☒ Tick this box to confirm that a figure exemplifying the gating strategy is provided in the Supplementary Information.
